# Dynamic and Catalytic Multiphase Coacervates

**DOI:** 10.1021/acs.biomac.5c02736

**Published:** 2026-07-09

**Authors:** Richard Booth, Jiaqi Pei, Jacob Shaffer, Christine D. Keating

**Affiliations:** Department of Chemistry, 311285 Pennsylvania State University, University Park, Pennsylvania 16802, United States

## Abstract

Catalytic reactions in multiphase coacervate systems are important for living cells, provide functionality to artificial systems, and may aid in understanding how metabolism first arose, but are not yet well understood. Here, we report an artificial multiphase system in which catalytically active droplets containing three phases undergo reorganization in response to pH, enabling control over chemical reactions. Catalysis is performed by an inner, polyhistidine-dense coacervate phase with inherent nonenzymatic activity, and the multiphase architecture stabilizes this otherwise aggregation-prone component against precipitation. We show that phase hierarchy provides catalytic design degrees of freedom fundamentally inaccessible in single-phase systems, including spatial decoupling of catalyst, substrate, and product that significantly enhances reaction rates, and that phase reorganization itself can be catalytically productive. By exploiting competitive interplay between intra- and intermolecular interactions between components, it was possible to (re)­organize and tune the reactivity of dynamic synthetic biological systems in ways unavailable to single-phase coacervates.

## Graphical abstract

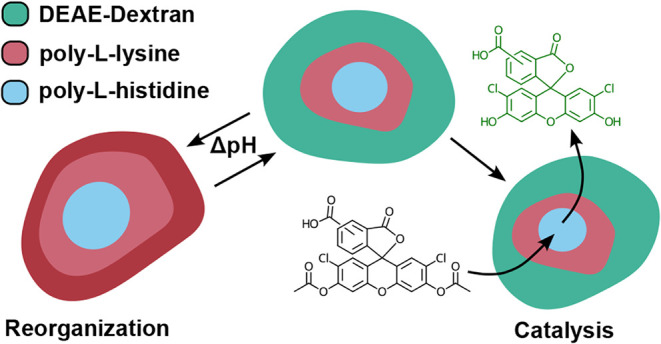

## 1 Introduction

Liquid–liquid phase separation (LLPS), and particularly associative LLPS or coacervation, has recently gained prominence both as a paradigm for understanding subcellular organization in biology and as a strategy for designing nonliving compartments with increasingly life-like properties. Coacervate droplets form through dynamic, multivalent interactions whose physicochemical nature can confer responsiveness to external stimuli including changes in pH,^1,2^ ionic strength,^3,4^ temperature,^5,6^ and illumination.^7,8^ The locally high concentrations of coacervate components generate distinct microenvironments that selectively accumulate molecular^9^ and macromolecular^10^ cargoes and can accelerate biochemical reactions.^11–13^

When multiple polyion components are combined, additional coexisting coacervate phases can emerge, with the number and arrangement of phases governed by the noncovalent interactions between polyions and their relative wetting properties.^14^ The organization of these coexisting phases is determined by their interfacial tensions, which are in turn ordered by the relative phase separation propensity of each polymer combination, reflecting factors such as salt stability and hydrophobicity.^14–17^ By rationally selecting polymer combinations based on the strength of their noncovalent interactions and relative hydrophobicity, it is therefore possible to engineer hierarchical multiphase droplet systems with defined internal architectures.^18^ To date, multiphase systems have been demonstrated that can reversibly form and dissolve in response to enzyme-mediated pH changes,^19,20^ possess selective RNA duplex thermodynamics,^21^ display additional phase behaviors when integrated with a chemical reaction cycle,^22^ and facilitate advanced biomolecular self-sorting into distinct groups when exposed to light or chemical stimuli.^23^ Desirable characteristics may also involve the selective uptake of reactants or the exclusion of products, which, when combined with a crowded hydrophobic interior, are known to accelerate a wide range of chemical reactions.^24^ The potential landscape for tuning properties to enhance chemical transformations expands with the introduction of each new phase in a multiphase system. However, effectively harnessing the increased complexity of noncovalent interactions in multiphase systems to catalyze and drive biochemical reactions remains challenging.

Complex coacervation typically involves two or more oppositely charged polymers that interact by ion pairing.^25^ Additional noncovalent interactions are often involved and can modulate LLPS conditions and coacervate properties including, hydrogen bonding, dipole–dipole, cation−π, π–π and hydrophobic interactions.^26^ Functional groups capable of engaging in noncovalent interactions beyond purely electrostatic charge–charge attraction play an important role in LLPS in biological systems. For example, arginine residues have been identified as key drivers of LLPS in proteins such as FUS, LAF1, and Ddx4.^27^ Substitution of arginine with lysine in these systems reduces phase-separation propensity despite maintaining net charge, highlighting that interaction type rather than charge alone governs LLPS behavior.^28^ This distinction arises from the planar, delocalized guanidinium group of arginine, which can simultaneously engage in cation−π, π–π, and bidentate hydrogen bonding interactions with a single acceptor. The primary ammonium group of lysine, in contrast, predominantly forms multiple monodentate hydrogen bonds and contributes hydrophobic interactions through its (CH_2_)_4_ alkyl linker. While it can still act as the cationic partner in cation−π interactions, it lacks the delocalized π system that enables guanidinum to engage in π–π stacking. Histidine is another amino acid capable of noncovalent interactions through both charge–charge interactions and aromaticity, but has not been studied as extensively as arginine. The near-neutral p*K*_a_ for histidine’s imidazole group coupled with its aromaticity makes this amino acid an interesting candidate to investigate the interplay between the noncovalent interactions that govern coacervation. In addition, histidine moieties are nearly ubiquitous in enzyme active sites with the histidine-serine-aspartate catalytic triad able to catalyze numerous reactions.^29^ The triad plays a prominent role in enzymes such as oxidoreductases,^30^ acyl transferases,^31^ esterases,^32^ and isomerases.^33^ Enzymes containing histidine-based active sites are prominent biocatalysts with numerous industrial applications such as in the production of biofuels^34^ and the cleaning of environmental contaminants such as plastics,^35^ pesticides^36^ and parabens.^37^ However, incorporating histidine moieties into de novo catalytic systems with comparable catalytic efficiencies to native systems remains elusive.

Incorporating polyhistidine (pHis) into complex coacervates offers the possibility to combine the inherent catalytic activity of histidine moieties with the ability of coacervate droplets to accumulate biomolecules and control local microenvironments, potentially leading to de novo soft matter catalytic systems performing with kinetics analogous to native enzymes.^38^ To date, studies of histidine-based coacervates have largely focused on His/Tyr-rich squid beak peptide-inspired sequences, (*GHGXY*)*n*, where *X* is variable,^39^ emphasizing their mechanical properties,^39,40^ drug-delivery potential,^41^ and pH-responsive binding to lipid membranes.^42^ In contrast to these sequence-tunable peptides, polyhistidine lacks compositional complexity but offers a simpler model for exploring histidine-driven catalysis; however, its strong tendency to aggregate and precipitate from solution above its p*K*_a_ presents a significant practical challenge.^43,44^ The hydrophobicity and potential for strong π–π stacking and cation−π interactions of imidazole moieties, while favorable for mechanical stiffness, can work against reactivity by driving aggregation of pHis peptides when their imidazole groups are predominantly in their catalytically active, uncharged form. Solubility can be increased by lowering the solution pH to protonate imidazole side chains, which enhances positive charge and hydrophilicity. However, the pH required to achieve this can fall far below biological levels, especially when pHis interacts with other biomacromolecules.^43^ Moreover, acidification negatively impacts function since only the neutral form of the imidazole moiety is catalytically active. We therefore wondered whether incorporating pHis within droplets comprising one or more coacervate phases might provide sufficient tunability of the microenvironment to prevent its aggregation while maintaining catalytic activity.

Herein, we show how pHis can be incorporated into stimuli-responsive, catalytically active multiphase coacervate droplets. Surrounding the pHis-rich inner phase with additional coacervate phases altered the molecular composition of the inner phase, increased stability against pH-induced aggregation, and enhanced catalytic activity. The phase organization was fully reconfigurable through changes in pH, induced either directly or through the activity of incorporated urease and glucose oxidase enzymes. We show that pH-sensitive changes in pHis self-association, governed by the interplay between electrostatic repulsion and imidazole π-stacking, drive phase reorganization and modulate catalytic rate. This process is further tunable through the choice of polyanion: ATP, which engages in cation−π and π–π interactions with the pHis imidazole, disrupts these conformational changes and suppresses catalytic activity in a manner that can be partially deconvoluted using phosphorylated adenosine analogues. The presence of additional coacervate phases enhances the esterase-like catalytic activity of the pHis phase by acting as a kinetic sink for the reaction product, relieving product inhibition. Finally, we demonstrate enzymatic control of reaction rates through pH modulation. Overall, this work shows that relatively hydrophobic single-phase coacervates can be stabilized and their catalytic activity enhanced through multicompartmentalization, paving the way for complex multicompartment systems capable of advanced chemical processing and programmable biocatalysis.

## 2 Experimental Section

### 2.1 Formation of Coacervates

All polyelectrolyte stock solutions were prepared to a final concentration of 50 mM monomer concentration in Milli-Q water. pHis was dissolved by adjusting the pH to 4.5–4.8 using 1 mM HCl (measured with Mettler Toledo pH sensor InLab Ultra Micro-ISM pH probe). Multiphase coacervates were generated by first preparing single-phase coacervates at a final monomer charge concentration of 10 mM for each polyelectrolyte and a molecular concentration of 10 mM for ATP. “Monomer charge concentration” refers to the molar concentration of ionic charges along the polymer backbone (e.g., pAsp contributes one negative charge per monomer unit, so 10 mM monomer charge concentration corresponds to 10 mM in monomer units). In contrast, for ATP, the concentration is expressed per molecule, irrespective of its multiple charges. The resulting single-phase coacervates were then combined to produce multiphase droplets. The three single-phase coacervates were made in the following order: pHis/ATP, pLys/pAsp, and DEAE-dextran/CM-dextran. pHis/ATP was prepared in acetate buffer (10 mM, pH 4.5), pLys/pAsp and DEAE-dextran/CM-dextran were both prepared in MES buffer (10 mM, pH 6). When mixed together, this combination gave a final pH of 5.7. The different pH buffers were required to increase solubility of pHis, while allowing a final pH after mixing of approximately pH 5.7. To achieve a final pH of 4.7, the three single-phase coacervates were all prepared in acetate buffer (10 mM, pH 4.5) before mixing. When making the single-phase coacervates, the cation was always added last to avoid the polycation irreversibly sticking to the surface of the plastic microcentrifuge tubes. Subsequent experiments on single-phase and multiphase systems were carried out within 1 h on coacervates prepared in this manner without noticeable degradation. The pH values of 4.7 and 5.7 were measured for the base coacervate system and are expected to apply to subsequent experiments, although minor variations may occur between preparations.

### 2.2 Raman Spectra and Raman Microscopy

Raman spectra were collected using a Horiba LabRam HR Evolution spectrometer equipped with a 532 nm laser line and a Freespace Olympus BX51 upright confocal microscope. Multiphase coacervates were prepared as described in the Formation of Coacervates section above at pH 5.7. For imaging with the upright microscope, samples were prepared by suspending coacervate droplets from the upper interior surface of silanized capillaries, which were loaded by horizontally dipping the tip of the capillary into the coacervate suspension. The capillaries were mounted onto supporting coverslips and sealed at both ends with Scotch tape. Droplets were allowed to equilibrate for at least 30 min before imaging. Imaging was performed using a Zeiss 100X oil immersion lens with the 532 nm laser operating at 100% power. To determine the optimal focal plane for droplet microRaman imaging, a Z-depth profile was acquired in 1 μm steps with 2 s of accumulation per step. The focal plane was selected based on the Z-depth that yielded the greatest integrated intensity in the C–H stretching region (2800–3182 cm^–1^). After selecting the appropriate focal plane, a Swift map was acquired with 0.99 s of accumulation per pixel to generate Raman images of the droplets.

### 2.3 Change in pH Using Glucose Oxidase and Urease Enzymes

Multiphase coacervates were prepared as described in the Formation of Coacervates section above with the extra addition of 0.2 mg·mL^–1^ of urease (when increasing the pH) or glucose oxidase (when decreasing the pH). The desired enzyme was added to the final solution while keeping the concentrations of the other components constant. The reaction was initiated by the addition of 5 mM urea (urease) or 15 mM glucose (glucose oxidase) which were both dissolved in Milli-Q water. The change in pH was measured using a pH Probe, taking a data point either every 30 s (urease) or 120 s (glucose oxidase) until the pH stabilized.

### 2.4 Imaging Change in pH Using Confocal Microscopy

Multiphase coacervates were prepared and doped with the relevant fluorescently labeled polyelectrolyte as described previously, with the addition of either glucose oxidase or urease enzymes (0.2 mg·mL^–1^). Labeled versions of the enzymes were not used in these experiments to preserve the stability and the kinetics of the enzymes. The coacervate solution was added to a PEGylated glass coverslip, the pH change reaction was initiated by addition of the relevant substrate (5 mM urea or 15 mM glucose) to the outer edge of the coacervate solution. Images were taken at a frequency of either every 30 s for experiments lasting 15 min or every 2 min for experiments lasting 60 min.

### 2.5 Histidine-Mediated Hydrolysis of CDFDA in Bulk Solution

Hydrolysis kinetics were collected using a Horiba FluoroLog-3 spectrofluorometer equipped with a Peltier temperature controller which was kept at 25 °C for all experiments. Multiphase coacervates were prepared using the same procedure as described above in the Formation of Coacervates section. 5(6)-Carboxy-2′,7′-dichlorofluorescein diacetate (CDFDA) was dissolved in DMSO to a concentration of 2 mM; this was subsequently diluted to a final concentration of 25 μM in Milli-Q water. This solution remained stable for approximately 5 h before precipitating, likely due to its tendency to aggregate in Milli-Q water. To initiate the reaction, first the multiphase coacervate was added to a cuvette and left to equilibrate to 25 °C for 2 min. Afterward, CDFDA (25 μM) was added to a final dye concentration of 5 μM. A data point was recorded every 2 s for a total of 900 s, using a λ_ex_ of 492 nm and a λ_em_ of 517 nm. The experiments were repeated in triplicate and averages and standard deviations were taken. The different pH values were obtained in the same manner as described in the coacervate preparation methodology. Single-phase coacervates were recorded by not adding the other two single phases and instead adding an equal volume of buffer. For the pH 5.7 system, a 3:1 ratio of MES buffer (10 mM, pH 6) to acetate buffer (10 mM, pH 4.5) was used. For the pH 4.7 system, only acetate buffer (10 mM, pH 4.5) was used. pH change experiments were carried out by adding urease (0.2 mg·mL^–1^) to the coacervates; the reaction was initiated using a solution of urea (25 mM) mixed with CDFDA (25 μM) to final urea and CDFDA concentrations of 5 mM and 5 μM, respectively.

### 2.6 Small-Volume Histidine-Mediated Hydrolysis of CDFDA

Coacervates were prepared as described in the Formation of Coacervates section above. For the multiphase system, only pHis was fluorescently labeled by doping with Alexa Fluor 633-labeled pHis20-C peptide, to avoid spectral overlap with the excitation and emission of CDFDA. The reaction was initiated by adding CDFDA to a final concentration of 20 μM. The hydrolysis was followed using the 488 nm laser, taking images every 30 s for 15 min. Different pH values were obtained by mixing acetate and MES buffer when preparing the coacervates, as described previously. pH change experiments were carried out by adding urease (0.2 mg·mL^–1^) to the coacervates; the reaction was initiated using a mixture of urea (25 mM) and CDFDA (25 μM) to final urea and CDFDA concentrations of 5 mM and 5 μM, respectively, and the reaction was imaged every 30 s for 1 h.

## 3 Results and Discussion

### 3.1 Phase Behavior of Histidine-Rich Multiphase Coacervates

In preparing pHis-based complex coacervates, we sought to identify a pH at which pHis would retain partial solubility while maintaining sufficient imidazole deprotonation for catalytic activity. The p*K*_a_ for free histidine is 6.0,^45^ though local microenvironments can shift this value substantially; p*K*_a_ values ranging from 5.4 to 7.6 have been reported for different histidine residues within a single protein.^46,47^ We therefore chose an initial bulk pH of ∼6, where pHis should be partially protonated and carry a net positive charge. Throughout this study, bulk pH values were measured using a calibrated electrode; however, coacervate phases can exhibit altered proton activity and apparent p*K*_a_ shifts relative to the surrounding solution due to local microenvironment effects.^48^ Accordingly, all pH values reported here refer to bulk measurements, with the recognition that the effective protonation state of pHis within the coacervate phase may differ.

We began by mixing pHis (polydisperse, 5–25 kDa), with the negatively charged polyion pAsp (poly-l-aspartic acid, PDI = 1.00–1.20, ∼11.5 kDa) as a partner to generate complex coacervate droplets. Optical microscopy revealed the formation of small (<5 μm) structures that appeared to be small droplets and gel-like aggregates (Supplementary Figure 1a). We hypothesized that the combination of high charge densities and high total charges of both pHis and pAsp contributed to the formation of aggregates. To reduce aggregation, we substituted pAsp with adenosine triphosphate (ATP disodium salt, 551.14 Da), a small, biologically relevant multivalent anion. The resulting droplets appeared larger and more spherical with reduced aggregation compared to the pAsp system (Supplementary Figure 1b).

We therefore chose ATP as the anion to pHis in constructing our initial multiphase droplets. These pHis/ATP coacervates were combined with additional coacervate phases, starting with pLys/pAsp (poly-l-lysine, PDI = 1.00–1.20, ∼16 kDa). To prevent aggregation of pHis upon contact with pAsp, multiphase coacervates were prepared from preformed single-phase coacervates rather than by mixing individual polymers together in a single step. Mixing the preformed pHis/ATP coacervates with pLys/pAsp coacervates resulted in two-phase droplets with an inner pHis-rich core and an outer pLys-rich shell (Supplementary Figure 2). Importantly, the pHis/pAsp single-phase coacervates were first formed at ∼pH 4.7, where histidine residues are more protonated and pHis remains more soluble and less prone to aggregation. These were then combined with additional coacervate phases prepared at ∼pH 6, resulting in a final multiphase system at ∼pH 5.7. This stepwise assembly, involving preformed coacervates prepared under controlled pH conditions, enabled the buildup of one coacervate phase on top of another without inducing nonspecific aggregation (Figure 1). The dilute phases were not removed prior to mixing, meaning that oppositely charged species from different coacervate systems are present in the same solution and may interact to some extent. Preformation of each single-phase coacervate prior to mixing was intended to minimize uncontrolled cross-system interactions; the well-defined phase segregation observed is consistent with this strategy effectively limiting, though not eliminating, such interactions. Further details of the mixing protocol and its rationale are provided in the Supplementary Methods.

**Figure 1:**
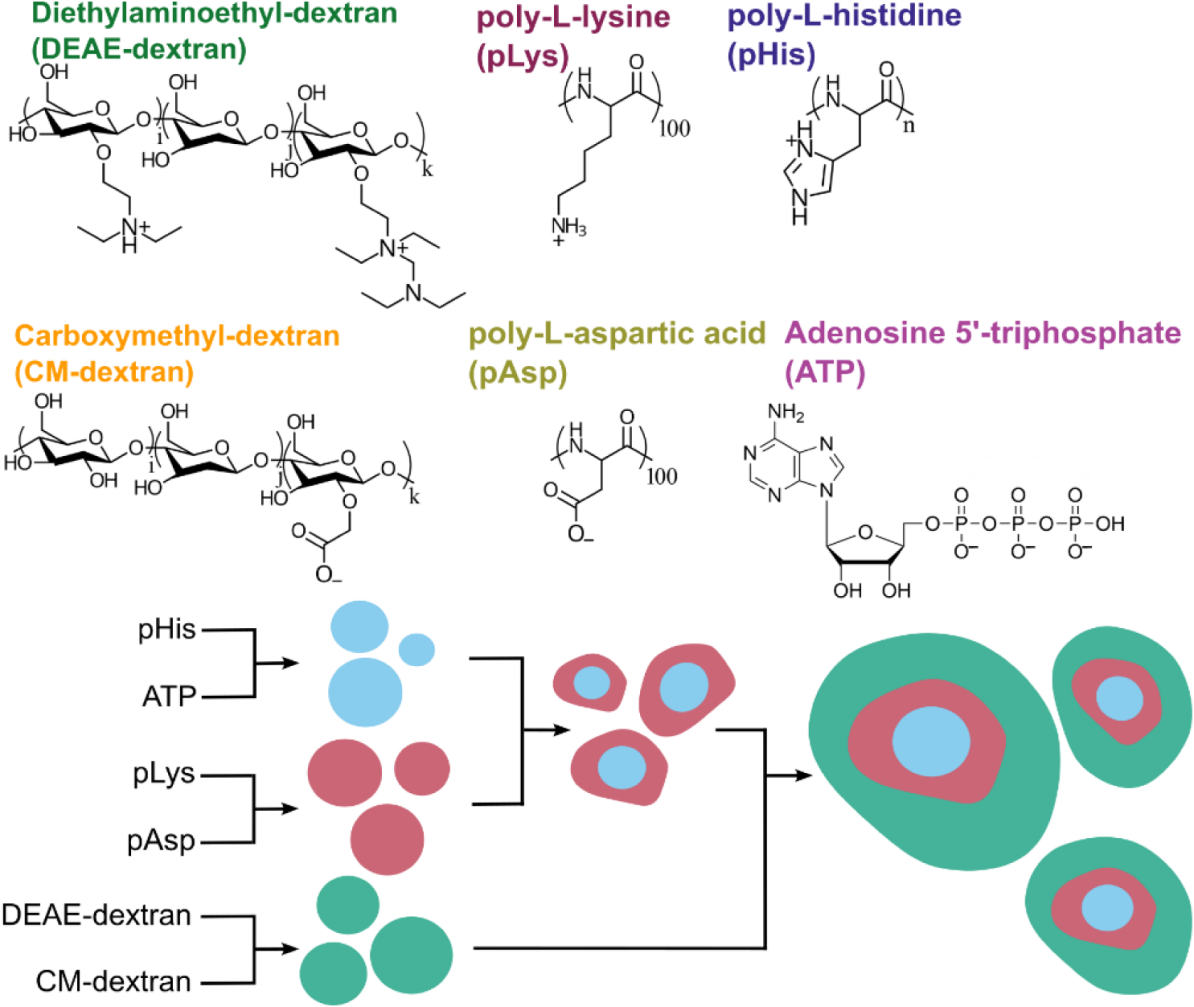
Schematic showing the molecules used in forming the individual single-phase coacervates and how these are combined to form the three-phase multiphase coacervate droplets.

Addition of a third coacervate system, composed of DEAE-dextran (average M.W. 40 kDa, positively charged) and CM-dextran (polydisperse, 40–60 kDa, negatively charged), generated three-phase droplets. Confocal fluorescence microscopy using labeled polycations revealed a three-layer architecture: an inner pHis-rich core, a surrounding pLys-rich middle phase, and an outer DEAE-dextran-rich layer (Figure 2a). Fluorescence intensity profiles confirmed that the three polycations were well segregated with minimal overlap between adjacent phases (Figure 2b). A separate experiment using labeled polyanions showed that both pAsp and CM-dextran were primarily distributed in the outer and middle coacervate phases, with less pronounced segregation compared to the cationic polymers (Supplementary Figure 3).

**Figure 2:**
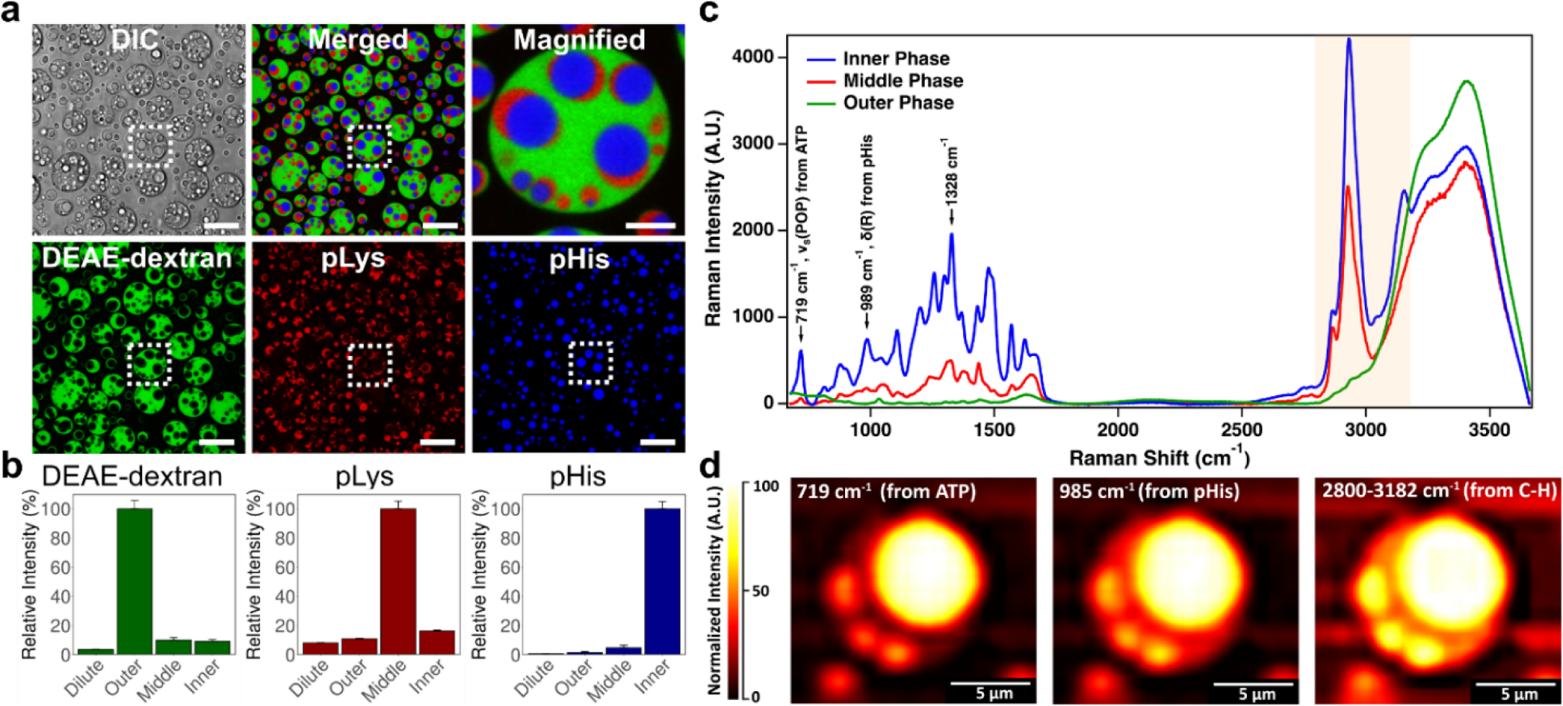
Morphology and molecular distribution in multiphase coacervates. **a)** Confocal fluorescence microscopy images of multiphase coacervates prepared with DEAE-dextran/CM-dextran, pLys/pAsp, and pHis/ATP at pH 5.7. **b)** Comparisons of relative fluorescence intensity for each of the fluorescently labeled cations in the multiphase system (L to R): DEAE-dextran-FITC (green), pLys-Rhodamine (red), and pHis-Alexa Fluor 633 (introduced via a fluorescently tagged 20His-C peptide, blue). Fluorescence intensities are normalized to the maximum signal for each dye, such that the phase with the highest intensity is set to 100%. Intensities in the other phases are expressed as a percentage relative to this maximum. Error bars represent standard deviations of a minimum of 20 droplets. **c)** Raman spectrum of pHis and ATP Raman stretches of multiphase coacervate droplets at pH 6. **d)** MicroRaman maps plotted by characteristic peak: (left panel) ATP distribution represented by phosphate stretching, νs (P–O–P), at 719 cm^–1^;^49,50^ (middle panel) pHis distribution represented by imidazole ring C–C rocking or scissoring δ (R) at 989 cm^–1^;^51,52^ (right panel) overall organic content, represented by C–H stretching from 2800 to 3182 cm^–1^. All maps have been normalized based on the area under their corresponding peaks. Scale bars are 10 μm unless otherwise noted.

ATP was not fluorescently labeled due to its small size and lack of suitable functional groups; instead, we used confocal microRaman microscopy to probe its distribution. Both pHis and ATP have characteristic strong Raman-active vibrational modes (Supplementary Table 1 and Supplementary Figure 4). microRaman data indicated that ATP was predominantly sequestered in the inner histidine-rich phase, consistent with π–π stacking between the nucleobase and imidazole rings (Figure 2c,d). These measurements also confirmed the location of pHis as mainly in the inner coacervate phase, consistent with fluorescence intensity data taken via confocal microscopy (Figure 2a,b). An optical image of a multiphase droplet shows good agreement between the microRaman map and the observed droplet structure (Supplementary Figure 5). MicroRaman spectroscopy could not clearly distinguish between the outer coacervate phase and the surrounding dilute phase, likely due to low concentrations of Raman-active functional groups in these phases.

### 3.2 pH Sensitivity of Histidine-Rich Multiphase Coacervates

Since pHis is more soluble at low pH where it is more fully protonated, and tends to aggregate above its p*K*_a_ (p*K*_a_ ≈ 6–6.5),^43,44,53^ its LLPS behavior should be inherently pH-dependent. We therefore sought to test the pH sensitivity of the pHis-containing multiphase coacervates. Lowering the pH from ∼5.7 to ∼4.7, thereby increasing the positive charge density on pHis, produced little change in the morphology of the multiphase coacervates (DEAE-dextran/CM-dextran, pLys/pAsp, pHis/ATP; Supplementary Figures 6–8), hereafter referred to as the ATP multiphase. The distribution of labeled cationic polymers across phases was similarly preserved between pH 5.7 and pH 4.7 (Supplementary Figures 6 and 8). Analysis of the relative intensities of anionic polymers revealed a comparable picture; however, a noticeable increase in CM-dextran was observed in the middle phase at pH 4.7 (Supplementary Figures 7 and 8), consistent with the increased cationic character of pHis at lower pH driving greater association with negatively charged components in that compartment. To assess the upper pH limit of multiphase stability, we prepared ATP multiphase coacervates at pH ∼8.5, well above the p*K*_a_ of histidine. pHis-rich coacervate droplets were still present under these conditions, demonstrating that the multiphase architecture can be maintained even at pH values where pHis is almost fully deprotonated. However, some differences were apparent compared to pH 6.5: the pHis-rich droplets were somewhat smaller at the higher pH, with a median diameter of 1.74 μm compared to 2.35 μm at pH 6.5, representing a ∼25% reduction in diameter (Supplementary Figure 9). Additionally, the pHis-rich droplets underwent a wetting transition at the higher pH, sitting at the interface of the pLys-rich phase rather than being fully engulfed within it as observed at pH 6.5. This change in wetting behavior is consistent with a pH-dependent shift in the relative interfacial tensions between phases, likely reflecting the altered interaction landscape of fully deprotonated pHis imidazoles at pH 8.5 and their reduced affinity for the other coacervate components under these conditions.

The relative insensitivity of the ATP multiphase coacervates to differences in pH can be rationalized by a coacervate-induced p*K*_a_ shift of the pHis imidazole groups, in which ion pairing with polyanions stabilizes the protonated state to higher pH than observed for free pHis.^48,54^ However, it could also indicate that pHis-ATP association is dominated by π–π and cation−π interactions between the adenine base and the imidazole ring, which are less sensitive to changes in pH than ion pairing alone. We reasoned that if these π-associated interactions were responsible for the observed pH insensitivity, substituting ATP with a polyanion incapable of π-stacking with pHis imidazoles should increase the pH sensitivity of the multiphase system. To test this, we replaced ATP with pAsp at the same monomer concentration, while leaving the other components of the multiphase system unchanged; this system is hereafter referred to as the pAsp multiphase. This ATP-free multiphase system indeed showed a strong dependence on pH, with a notable difference in phase morphology and polyion distributions for different pH values. At ∼pH 5.7, the pAsp multiphase system resembled the ATP multiphase system (compare Figure 3a,b with Figure 2a,b). However, when the pH of the pAsp multiphase system was lowered to 4.7, the distinct outer DEAE-dextran-rich coacervate phase was no longer visually distinguishable, and an additional pLys-rich phase emerged, surrounding the inner pHis-rich phase (Figure 3c). This does not reflect the removal of DEAE-dextran from the system, but rather a change in its partitioning behavior. At lower pH, DEAE-dextran no longer forms a sufficiently concentrated coacervate phase under these conditions and instead distributes more evenly between the dilute and coacervate phases, and therefore does not appear as a distinct outer layer.

**Figure 3:**
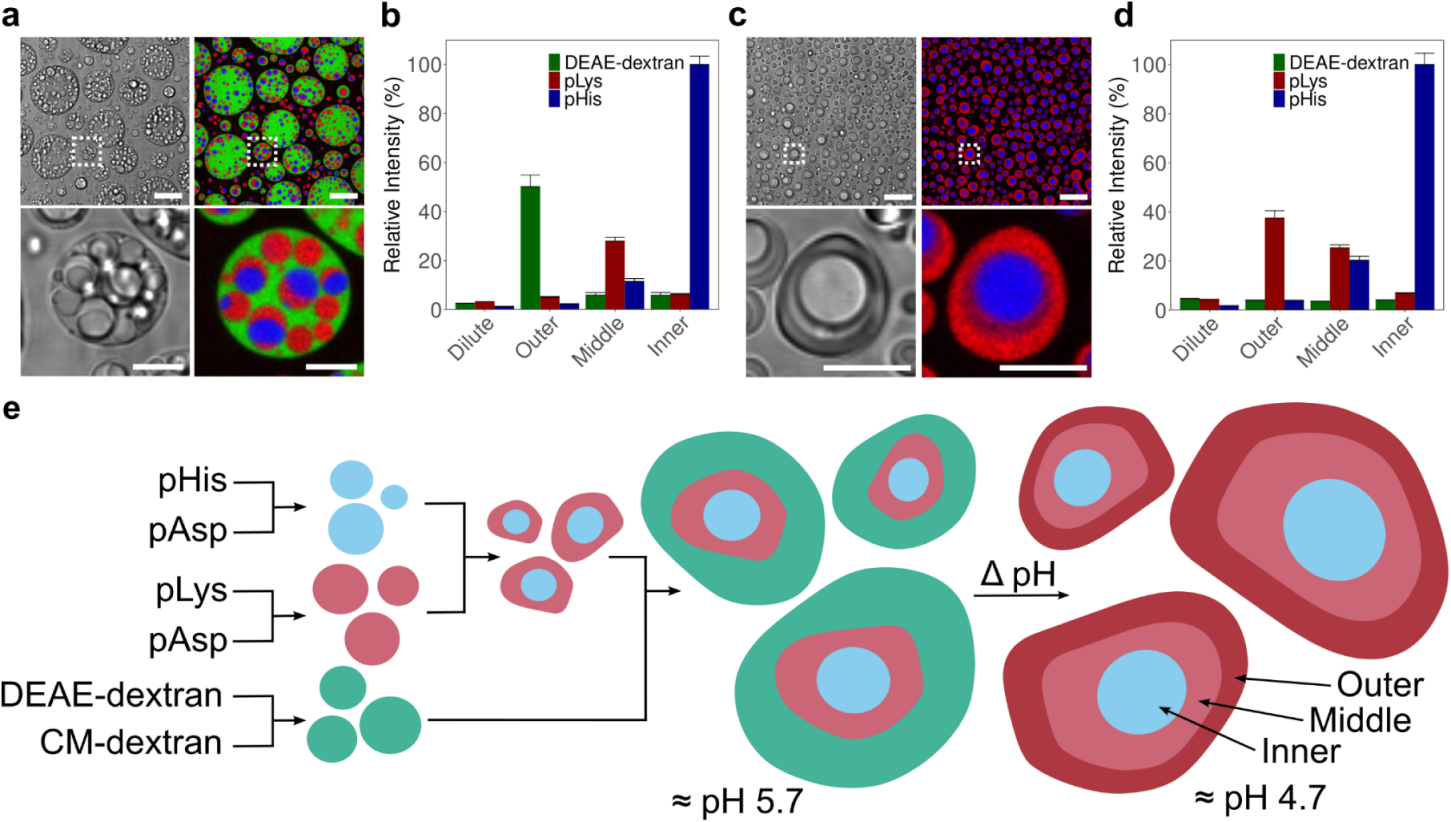
Multiphase coacervate pH sensitivity. **a,c**) Confocal fluorescence microscopy images of pAsp multiphase coacervates at ∼pH 5.7 **(a)** and at pH 4.7 **(c)**. **b,d)** Corresponding bar plots of the relative intensity of each of the fluorescently tagged cations DEAE-dextran-FITC (green), pLys-Rhodamine (red), and pHis-Alexa Fluor 633 (blue) in the coacervate multiphase at ∼pH 5.7 **(b)** and ∼pH 4.7 **(d).** Intensities are normalized such that 100% corresponds to the maximum intensity across all dyes and all phases; other dyes and phases are scaled relative to this value. A minimum of 20 measurements were collected per phase. Error bars represent standard deviations. Scale bars are 10 μm (full image) and 2.5 μm for the magnified image that represents the area of the dashed white line box. Error bars in **b,d** represent standard deviations of a minimum of 20 droplets. **e)** Schematic representation of the pH-mediated rearrangement of polyions in the multiphase.

The disappearance of a concentrated DEAE-dextran-rich phase also cannot be attributed solely to pH-dependent changes in fluorescein intensity, since in the ATP-containing multiphase system the fluorescein-tagged dextran remains clearly visible at the same lower pH. Although FITC exhibits increased fluorescence at higher pH, phase assignment here is based not only on intensity changes but also on relative partitioning of multiple labeled polymers and morphological characteristics. Fluorescence imaging with labeled polycations revealed similar pLys fluorescence intensities in both lysine-rich phases (outer and middle coacervate phases) at ∼pH 4.7, while the middle coacervate phase also contained pHis in addition to pLys and the inner phase was predominantly composed of pHis with minimal signal from other cationic components (Figure 3d). MicroRaman data were broadly consistent with the confocal microscopy results at both pH values, while also revealing an unexpected degree of pH-dependence in the partitioning of ATP between phases. Quantitative analysis of characteristic Raman peaks confirmed that both ATP and pHis were significantly enriched in the inner phase relative to the middle and outer coacervate phases at both pH values, while pLys was most abundant in the outer coacervate phase (Supplementary Figures 10 and 11). Normalizing each component’s characteristic peak intensity to the overall organic C–H stretching signal further showed that the ATP-to-organic and pHis-to-organic ratios within the inner phase increased with increasing pH, while the pLys-to-organic ratio remained unchanged, indicating that pHis and ATP partitioning is pH-sensitive whereas pLys partitioning is not (Supplementary Figure 12). Comparison of the Raman spectra at pH 4.5 and pH 6.0 also revealed greater hydroxyl stretching intensity (3200–3550 cm^–1^) in the middle and inner phases at pH 4.5 (compare Figure 2c with Supplementary Figure 13), consistent with higher water content and a less tightly condensed inner phase when pHis is more fully protonated. pH-dependent shifts in the pHis imidazole ring vibrational modes at 1203 and 1568 cm^–1^ within the inner phase further confirmed that the protonation state of pHis changes with bulk pH inside the condensed phase (Supplementary Figure 14). Overall, the ATP-free pAsp multiphase system resembled the ATP-containing multiphase system at ∼pH 5.7 but, at lower pH (∼4.7), underwent a reorganization characterized by loss of a discrete DEAE-dextran-rich outer phase and formation of an additional pLys-rich phase (Figure 3e).

We hypothesized that the pH-responsiveness of the pAsp multiphase system arises from changes in the self-association behavior of pHis above and below its apparent p*K*_a_ (∼5.0–5.5 in the pAsp system), driven by variations in π–π and cation−π interactions between neighboring imidazole groups as the pH shifts. At ∼pH 4.7, a greater proportion of pHis imidazole groups are protonated, increasing electrostatic repulsion between chains and promoting a more expanded conformation. This expanded state would be expected to enhance heterotypic interactions between pHis and the other coacervate components (pAsp, pLys, CM-dextran, and DEAE-dextran) through hydrogen bonding and electrostatics. In contrast, at ∼pH 6, a greater proportion of imidazoles are deprotonated, enabling π–π stacking between neutral imidazole rings and cation−π contacts between the remaining protonated and newly neutral imidazoles; in coacervate systems, such π-associated interactions and electrostatic interactions are competitive rather than additive, such that reduced charge density favors their formation.^55^ Together these interactions would promote tighter intra- and intermolecular packing of pHis chains, reducing their availability for heterotypic interactions with other coacervate components.

To examine this behavior directly, we recorded circular dichroism (CD) spectra and dynamic light scattering (DLS) measurements for pHis in solution at pH 4.5 and 6.0, either alone or in the presence of each polyanion (Supplementary Figures 15–17).

pHis alone undergoes a transition from isolated, random coils to assembled β-sheet-like structures as the pH is increased from 4.5 to 6.0,^56^ consistent with reduced electrostatic repulsion and increased associative interactions due to greater side-chain deprotonation near the p*K*_a_ (Supplementary Figure 15). Addition of pAsp to pHis causes only a slight perturbation of the CD spectrum, even at elevated concentrations, whereas ATP addition results in a pronounced reduction of the CD signal at either pH, even at lower concentration (Supplementary Figure 16). This loss of CD signal with ATP reflects disruption of pHis secondary structure, likely due to some combination of π–π, cation−π, and hydrogen bonding interactions between ATP and pHis, dependent on histidine protonation state, that interfere with imidazole–imidazole contacts stabilizing the secondary structure, even at low pH where ATP is less effective at promoting assembly.^46^ The presence of π–π and cation−π interactions, possible with ATP but not pAsp, may underlie the resistance of the ATP-containing multiphase system to strong pH-driven reorganization.

Given that the β-sheet-like state of pHis is associated with self-assembly, these polyanion-induced changes in secondary structure should also be reflected in the size distribution of pHis assemblies, which we probed by DLS. Addition of a small amount of ATP or pAsp to pHis (1:100 by monomer) drives self-assembly of pHis; however, the pHis/ATP system is pH-dependent, showing larger assemblies only at higher pH, whereas the pHis/pAsp system forms larger assemblies at both pH values tested (Supplementary Figure 17 and Supplementary Table 2). Size-binned analysis indicates that the larger assemblies (100–300 nm) form narrow distributions (PDI 0.14–0.18) with pAsp regardless of pH, but with ATP only at elevated pH. The elevated cumulant PDIs for pHis alone at pH 6.0 and pHis/ATP at pH 4.5 (both ∼0.57) instead likely arise from bimodality of the size distribution, with free or weakly associated pHis coexisting alongside the larger assemblies. The greater propensity for pHis/pAsp association is consistent with the larger size and higher (more negative) net charge of pAsp compared with ATP.^57^ We argue that ATP more poorly drives pHis assembly at either pH and that the trend in pH dependency observed via DLS reflects that of free pHis; thus, pH-dependent changes in assembly are not causative of the pH-dependent phase behavior.

From this data, we hypothesize that at low pH, ion pairing and hydrogen bonding dominate, thereby allowing both polyanions to drive LLPS at sufficient concentrations. At high pH, however, three factors lower pHis affinity for pAsp: increased imidazole–imidazole π interactions, reduced net positive charge on pHis, and reduced potential for imidazole–carboxylate hydrogen bonds. As a result, pAsp accumulates in other phases, driving pH responsiveness. Disruption of pHis π interactions by ATP at higher pH suggests that the loss of ion pairing occurs concurrently with an increase in π interactions, leading to a similar distribution of ATP at either pH.

To further explore the role of CM-dextran and pAsp in the pH-responsive pAsp multiphase, we used fluorescently tagged pAsp and CM-dextran to observe their spatial distributions as the pH changes. At ∼pH 4.5, pAsp is predominantly distributed between the middle and outer phases, with approximately 2× higher apparent concentration in the middle phase than in the inner phase and ∼1.5× higher in the outer phase than in the inner (Supplementary Figure 18). This likely reflects the dominance of imidazole–imidazole and imidazole–pLys interactions at low pH, which reduce pHis availability for interaction with pAsp. CM-dextran is primarily located in the outer phase at ∼pH 4.5, where its concentration is ∼5× greater than in the inner phase (Supplementary Figure 19), consistent with its weaker hydrogen-bonding capacity with pHis compared to pLys or pAsp.

At ∼pH 6, both pAsp and CM-dextran showed higher partitioning into the inner and outer phases relative to the pLys-rich middle phase, with normalized intensities approximately 2× and 5× higher than in the middle phase, respectively. We note that this apparent enrichment of polyanions in the inner phase at pH 6 should be interpreted with caution, as the aromatic fluorophores covalently attached to pAsp and CM-dextran may themselves engage in π–π stacking with neutral pHis imidazoles at higher pH, artificially enriching labeled polymer in the inner phase independently of the unlabeled polymer distribution. This interpretation is supported by the behavior of the ATP multiphase system, where the aromatic adenine base of ATP is preferentially enriched in the inner pHis-rich phase through π–π stacking interactions, demonstrating that aromatic groups in general partition favorably to this phase; the fluorophores attached to pAsp and CM-dextran could behave analogously. Additionally, neutral imidazole rings can act as both hydrogen bond donors and acceptors at higher pH, which may also contribute to some retention of pAsp and CM-dextran in the inner phase. Taken together, these findings indicate that the preferred interaction partners of pHis shift substantially with pH, with a range of heterotypic interactions between pHis and the other coacervate constituents contributing to the system’s pH responsiveness.

### 3.3 Enzyme-Mediated Change in pH and Morphological Changes in Multiphase Coacervates

To explore the morphological changes in the multiphase system that occur with a change in pH, we used two complementary enzyme systems: urease, which raises pH by generating ammonia from urea, and glucose oxidase (GOx), which lowers pH by converting glucose to gluconic acid.^19,20^ Together these allowed us to span a target pH range from ∼pH 4.5 to ∼pH 6 and vice versa, bracketing the p*K*_a_ of pHis in both directions and transitioning between a system lacking a DEAE-dextran-rich outer phase at ∼pH 4.7 and one possessing it at ∼pH 6 (Figure 4a, Supplementary Figure 20). We first prepared fluorescently labeled GOx and urease to assess their sequestration properties and stability within the multiphase. Confocal microscopy showed that both enzymes were preferentially sequestered in the inner pHis-rich phase, with an intensity approximately 2× higher than in the middle and outer phases (Supplementary Figure 21), consistent with the well-documented tendency of coacervate droplets to sequester proteins and enzymes.^24^ The urease reaction was initiated by adding 5 mM urea, and the resulting changes in droplet structure were monitored via confocal microscopy. This revealed that the initial two pLys-rich phases merged into one, followed by the emergence of a DEAE-dextran-rich outer phase (Figure 4b,c, Supplementary Figure 22 and Supplementary Video 1). As the pH increased, the number of coacervate droplets also increased, likely due to the increased hydrophobicity of deprotonated pHis imidazoles near and above the p*K*_a_, promoting droplet budding (Supplementary Figure 23a and Supplementary Video 1). Droplet settling is unlikely to account for this increase given the duration of the experiment (1 h) and the high initial droplet density which accelerates the rate at which droplets settle on the coverslip.

**Figure 4:**
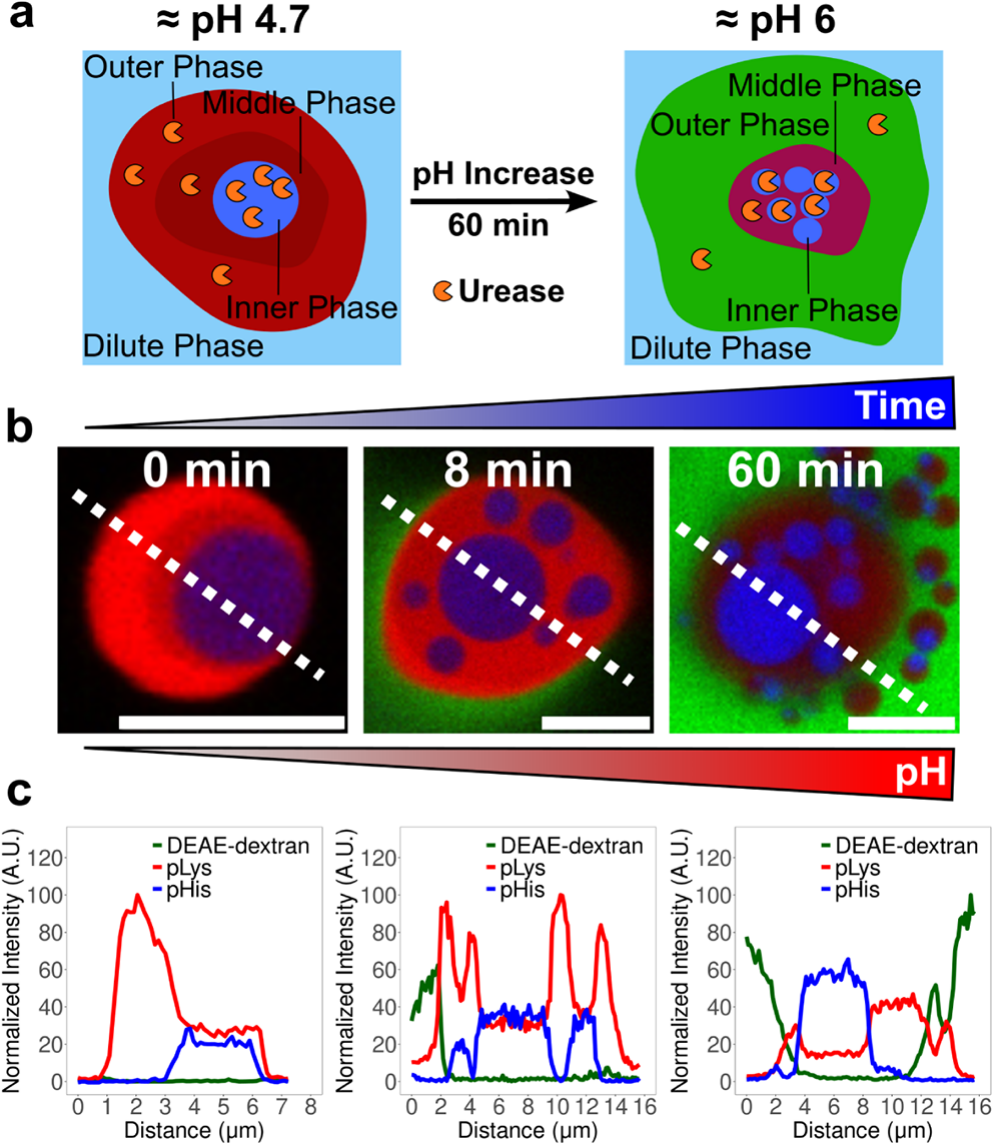
Enzyme-mediated phase reorganization in multiphase coacervate droplets. **a)** Schematic illustrating the morphological differences between the pAsp multiphase system at ∼pH 4.5 and ∼pH 6, showing the approximate locations of polyelectrolytes and enzymes at each pH extreme. **b)** Confocal microscopy images of the pAsp multiphase system over time as pH increases due to urease-mediated hydrolysis of urea (5 mM), producing ammonia and driving a pH increase from ∼pH 4.5 to ∼pH 6. Dashed lines indicate the positions of the corresponding line profiles shown in **c**. **c)** Line profiles showing the redistribution of fluorescently labeled components and the change in phase morphology over the course of the reaction (profiles correspond to dashed lines in **b**. The pH of the coacervate mixture was measured using a pH probe before and after the reaction to determine the extent of the pH change. Scale bars: 10 μm.

To change the pH in the opposite direction and decrease the pH from ∼5.7 to ∼4.5, 15 mM glucose was added to GOx-containing multiphase coacervates. As the pH decreased, the DEAE-dextran-rich phase slowly dissipated and a second pLys-rich phase emerged, as confirmed by confocal microscopy and line profile analysis (Supplementary Figure 24a and Supplementary Video 2). It is also notable that the mean diameter of the pHis-rich droplets increased due to coalescence as the pH decreased, consistent with a decrease in coacervate viscosity as pHis becomes more protonated and imidazole–imidazole π interactions are disrupted. This increase in diameter occurred over a period of approximately 5 min, beginning around 20 min after the start of the reaction (Supplementary Figure 23b,c). The 20 min time point corresponds to a pH of ∼5.6 (Supplementary Figure 20), close to the p*K*_a_ of pHis, consistent with protonation-driven changes in imidazole interactions reducing condensate viscosity and promoting droplet coalescence at this pH.

We further investigated this system by repeating the pH increase reaction using labeled CM-dextran. Initial images at ∼pH 4.5 confirmed that CM-dextran was primarily present in the outer pLys-rich phase (Supplementary Figure 24b and Supplementary Video 3), consistent with our earlier partitioning data (Supplementary Figure 19). As the pH increased toward ∼pH 6, the second pLys-rich phase dissipated and was progressively replaced by a distinct CM-dextran-rich outer phase, with CM-dextran visibly redistributing between phases during the transition. Given the dynamic redistribution of CM-dextran observed here, and the broader role of polyanion-pHis interactions in driving pH-dependent phase reorganization, we also monitored the movement of pAsp during the pH increase reaction. Both CM-dextran and pAsp showed clear redistribution between phases as the pH changed (Supplementary Video 4), consistent with our earlier static partitioning data at pH 4.5 and 6 (Supplementary Figures 18 and 19), and confirming that pH-dependent changes in polyanion-pHis interactions play a prominent role in the reorganization of the multiphase system.

### 3.4 Esterase-Like Activity in Multiphase Coacervates

Given the reported inherent catalytic activity of the histidine-rich polymer,^58^ we explored whether our multiphase coacervates could hydrolyze ester-based substrates and act as an artificial enzyme (Figure 5a). We selected 5(6)-carboxy-2′,7′-dichlorofluorescein diacetate (CDFDA), which is initially nonfluorescent but converts to the fluorescent product 5(6)-carboxy-2′,7′-dichlorofluorescein (CDF) upon hydrolysis of the acetate esters (λ_ex_ 492 nm, λ_em_ 517 nm).^59^ We chose this substrate because its fluorescence quantum yield increases substantially above pH ∼5, making it well-suited for measurements across our experimental pH range of 4.7 to 5.7, though this pH sensitivity of the fluorescence signal also necessitated a correction factor for quantitative comparisons between pH values, as described below.^60^ We note that only bulk pH was monitored in these experiments and we cannot be certain of the local proton activity within individual phases; coacervate phases can exhibit shifted apparent p*K*_a_ values due to local dielectric properties and ion pairing.^47^ First, we tested the sequestration properties of the CDF product by confocal microscopy (Figure 5b). The fluorescent CDF is predominantly localized in the inner histidine-rich phase, showing a relative intensity ∼2× greater than in the middle and outer phases at both pH ∼4.5 and ∼5.7 (Figure 5c). Based on the sequestration behavior of CDF within the multiphase system, we expected the nonfluorescent CDFDA substrate to behave similarly and localize in the catalytically active inner phase. Upon adding CDFDA at ∼pH 5.7, confocal microscopy confirmed that the substrate was hydrolyzed by the pHis-rich inner phase, with strong green fluorescence developing there over 900 s before diffusing outward into the surrounding phases (Figure 5d,e, Supplementary Figure 25, Supplementary Video 5). Although equilibrium partitioning favors the inner pHis-rich phase, we hypothesized that at the onset of the reaction the product concentration in the outer phases would be negligible, establishing a concentration gradient that drives net diffusion of CDF outward. Under this scenario, the outer phases would act as a kinetic sink during the early reaction period, relieving product inhibition within the pHis-rich phase. This kinetic effect would be most pronounced at early time points, providing a testable prediction that we examine below.

**Figure 5:**
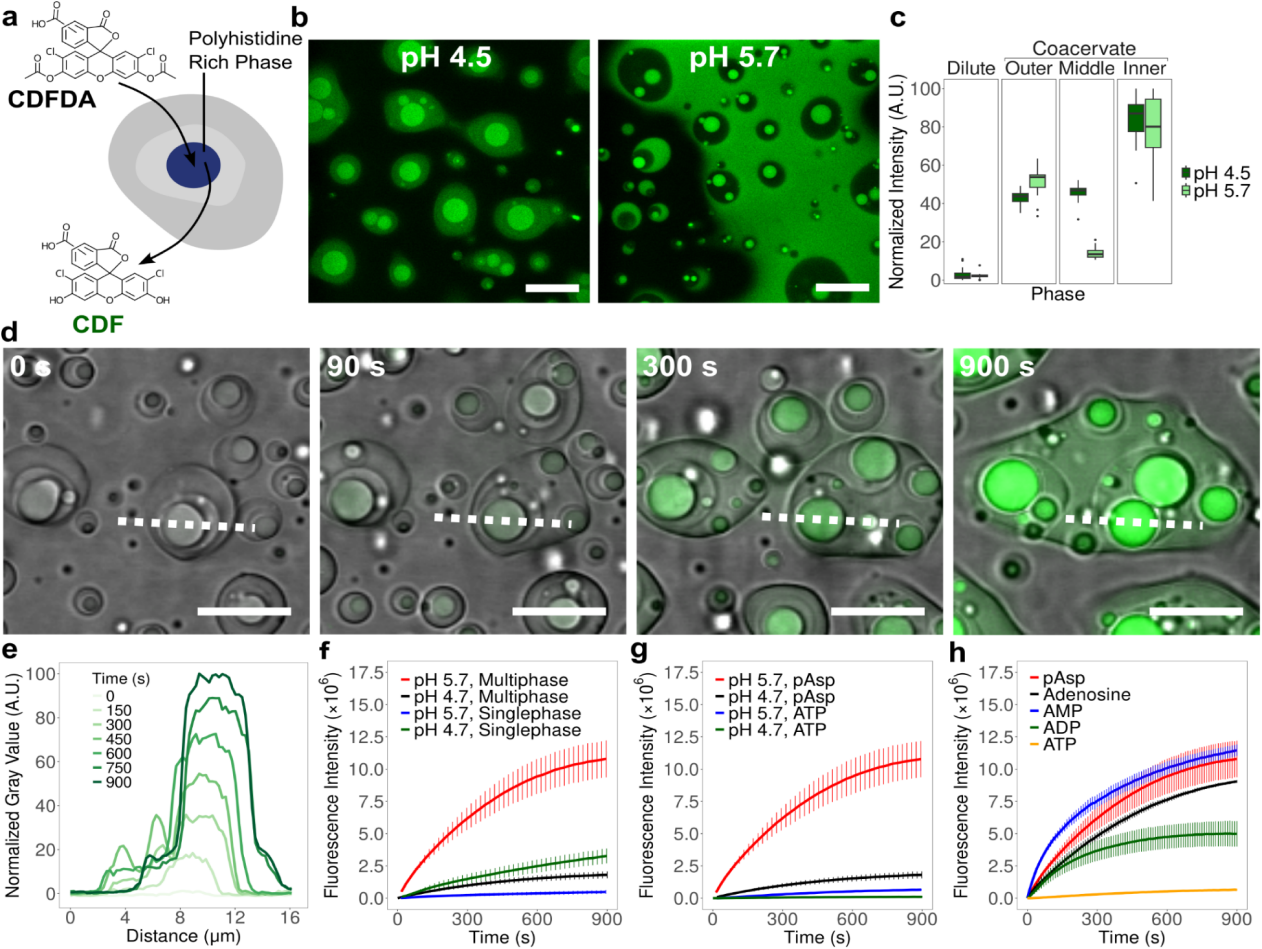
Esterase-like hydrolysis in multiphase coacervates. **a)** Schematic showing the addition of the ester-containing substrate CDFDA and its subsequent hydrolysis by the inner pHis-rich phase of the multiphase system to yield the fluorescent product CDF. **b)** Merged confocal microscopy images (DIC and green fluorescence channel) showing CDF sequestration in the pAsp multiphase system at pH 4.5 and 5.7. Note that the outer phase has coalesced to form a near-continuous layer at pH 5.7. **c)** Box plots showing the normalized fluorescence intensity (min-max) of CDF in each phase of the multiphase system at pH 4.5 and pH 5.7. Each pH condition was normalized independently; a minimum of 20 intensity values were collected per phase. **d)** Merged confocal microscopy images (DIC and green fluorescence channel) showing the formation of CDF over 900 s in the CM-dextran/DEAE-dextran, pLys/pAsp, and pHis/pAsp multiphase system. **e)** Line profiles showing the increase in fluorescence intensity across the multiphase over the course of the 900 s reaction (profiles correspond to dashed lines in **d**. **f-h)** Fluorescence spectroscopy kinetics of CDFDA hydrolysis (λ_ex_ 492 nm, λ_em_ 517 nm; arbitrary units) showing: **f)** the multiphase and single-phase pHis/pAsp coacervates at pH 4.7 and 5.7; **g)** the multiphase system in the presence and absence of ATP at pH 4.7 and 5.7; and **h)** the multiphase system in the presence of pAsp, adenosine, AMP, ADP, and ATP at pH 5.7. Error bars in **f-h** represent standard deviations from a minimum of 3 independent measurements per condition. Scale bars: 10 μm.

In order to get an accurate measure of the kinetics of the reaction by fluorescence spectroscopy, we first tested the pH sensitivity of free CDF product by taking fluorescence spectra of free dye at pH 4.7 and 5.7 to calculate a correction factor for pH-dependent fluorescence (Supplementary Methods and Supplementary Figure 26). Comparison of the rate of hydrolysis in the multiphase at pH 5.7 and 4.7 by fluorescence spectroscopy revealed that the rate of hydrolysis was ∼7× greater at the higher pH (Figure 5f and Supplementary Table 3). This is consistent with a greater proportion of pHis imidazoles being deprotonated at pH 5.7, freeing the lone pair on the unprotonated imidazole nitrogen for nucleophilic catalysis.

The rate of reaction was also considerably faster in the multiphase system than in the single-phase pHis/pAsp coacervates, with an apparent average increase in rate of ∼6× across both pH values (Figure 5f and Supplementary Table 3). These data support our hypothesis that the outer phases act as a kinetic sink at the onset of the reaction, driving net diffusion of product away from the pHis-rich phase and transiently relieving product inhibition. This spatial decoupling of catalyst and product is a direct consequence of multicompartmentalization and cannot be replicated by adjusting pHis concentration or macromolecular crowding within a single-phase system.

A further notable observation is that within the single-phase coacervates, the hydrolysis rate was faster at pH 4.7 than at pH 5.7, which is the opposite trend to that seen in the multiphase system (Figure 5f and Supplementary Table 3). This counterintuitive result can be understood in terms of the competing effects of pH on catalytic competency and chain mobility. At pH 5.7, deprotonation of pHis imidazoles increases lone pair availability for nucleophilic catalysis, but simultaneously promotes strong π–π and cation−π self-association between imidazole rings, increasing coacervate viscosity and reducing substrate accessibility and chain mobility, consistent with the CD-observed structural transitions described above (Supplementary Figures 15 and 17) and supported by interaction energy calculations^61^ and condensate simulation data.^55^ A possible explanation for this is that without the multicompartment kinetic sink to relieve product inhibition, these mobility penalties would outweigh the catalytic benefit of greater lone pair availability, resulting in the slowest observed rate in the single-phase system at pH 5.7. At pH 4.7, pHis is more protonated and therefore more mobile and less self-associated, partially offsetting the reduced catalytic activity and producing an intermediate rate. In the multiphase system at pH 5.7, the catalytic benefit of deprotonation is preserved while the mobility penalties are mitigated by two complementary mechanisms. First, continuous product removal by the outer phases relieves product inhibition. Second, the complex multicomponent environment of the inner phase, which includes pLys, CM-dextran, DEAE-dextran, and pAsp in addition to pHis, provides competing heterotypic interactions that distribute the interaction network across many different partners, reducing the extent of homotypic imidazole–imidazole π-stacking that drives up viscosity in the single-phase system.^55^ Multicompartmentalization therefore modulates not only the kinetics of product removal but also the physical properties of the catalytic phase itself, explaining why the multiphase system at pH 5.7 achieves the highest observed rate.

Control experiments without pHis showed negligible background hydrolysis, even when multiphase coacervate droplets (DEAE-dextran/CM-dextran, pLys/pAsp) were present to enhance the solubility of CDFDA (Supplementary Figure 27 and Supplementary Table 3), confirming that the observed catalytic activity is attributable specifically to the pHis-rich phase.

The spectra of free dye at the two pH values also enabled us to calculate how close to completion the coacervate systems are by comparing the fluorescence intensity of the reaction mixture to that of the free dye (Supplementary Table 3). The multiphase system at ∼pH 5.7 reached approximately 20% completion after 900 s, which was about six times higher than the ∼3% completion observed at ∼pH 4.7. However, when comparing the multiphase system to free pHis polymer in homogeneous solution, the free polymer reached approximately 40% conversion, roughly twice that observed for the multiphase droplets (Supplementary Table 3 and Supplementary Figure 28). This difference likely reflects the increased viscosity within the coacervate environment, which reduces molecular mobility and mass transport and can partially offset concentration effects. Our goal was to understand how compartmentalization and phase hierarchy modulate catalytic behavior within complex coacervate architectures. In this context, the key comparison is between single-phase and multiphase coacervate systems under identical conditions, where the introduction of additional phases enables measurable modulation and enhancement of catalytic activity within a compartmentalized environment. While free pHis displays higher apparent catalytic efficiency in homogeneous solution, it lacks structural organization, environmental responsiveness, and phase-dependent regulation. In contrast, the multiphase coacervate architecture enables spatial segregation, dynamic reorganization in response to pH, and controlled modulation of catalytic behavior, features that are not accessible in solution-phase polymer systems.

Comparison of the pAsp system and the ATP system showed hydrolysis rates decreased by ∼50× when ATP was present in the system (Figure 5g). We hypothesized that this was due to ATP’s strong interactions with the pHis imidazole groups through both the adenine group π–π and cation−π interactions with the imidazole ring and the negative charge of the phosphate electrostatically bonding to the positively charged histidine residues. The imidazole ring of histidine is capable of engaging in π–π stacking interactions with aromatic systems such as the adenine base of ATP with energies of −3.0 to −4.0 kcal/mol, and in cation−π interactions when protonated, with energies up to −13.6 kcal/mol depending on the aromatic partner.^61^ Direct stacking of an aromatic group onto a catalytic histidine imidazole is sufficient to substantially reduce hydrolytic activity, as demonstrated in enzyme systems where introduction of competing aromatic contacts in the vicinity of the catalytic histidine produces large decreases in rate.^62,63^ While a coacervate lacks the ordered active site geometry of an enzyme, an analogous effect is expected here: π-stacking of the adenine base onto pHis imidazole groups would sterically shield the lone pair required for nucleophilic catalysis, reducing the fraction of imidazoles available to interact with the substrate. To further investigate the effect of ATP on the catalytic activity, we compared the effect of ATP with analogues having fewer phosphate groups: ADP, AMP, and adenosine. Fluorescence spectroscopy showed an increase in rate as the phosphates are removed with ATP exhibiting the slowest kinetics and AMP the fastest (Figure 5h). The AMP system even outperformed the ATP-free pAsp system in the initial time points exhibiting kinetics that were ∼2× faster (Figure 5h and Supplementary Figure 29). Removing all phosphates from ATP did not produce further rate enhancement: adenosine kinetics fell between those of AMP and ADP rather than exceeding AMP as a charge-only model would predict (Figure 5h). This result indicates that the adenine base itself contributes to catalytic inhibition, likely through π-associated interactions with the pHis imidazole, independently of phosphate charge or droplet morphology. Other factors such as solubility, hydrogen bonding of phosphate oxygens, and effects on phase properties may also contribute to the more complex trend. Taken together, these results indicate that coacervation is primarily driven by electrostatic complexation between phosphate and pHis, while nucleotide identity modulates catalytic activity through additional π-associated interactions between the adenine base and pHis imidazole groups.

To further investigate how pAsp and ATP influence the catalytic activity of pHis, we performed fluorescence recovery after photobleaching (FRAP) on fluorescently labeled pHis in both systems. In the single-phase coacervates, FRAP revealed slower recovery in the ATP-containing system compared to the pAsp-containing system, indicating that pHis is more mobile in the presence of pAsp. This increased mobility suggests greater accessibility of catalytically active imidazole groups to interact with the substrate (Supplementary Figures 30 and 32a). FRAP for the ATP multiphase and pAsp multiphase coacervates also showed ATP coacervates had slower recovery, potentially indicating that the ATP system is more viscous, and that this increased viscosity could be contributing to the slower kinetics when ATP is present (Supplementary Figures 31 and 32b). A smaller difference in the rate of recovery between pAsp and ATP was observed in the multiphase system compared with the single-phase system, indicating that the presence of the multiphase reduced the disparity in recovery rates by enhancing recovery in the ATP system but not in the pAsp system. The slow recovery times observed across both systems are consistent with the known tendency of aromatic residues capable of π–π and cation−π interactions to produce highly viscous condensates with reduced internal mobility. Arginine-rich condensates, which share with histidine the capacity for π-stacking and cation−π interactions, exhibit approximately 100-fold greater viscosity than comparable lysine-rich condensates,^18^ and analogous behavior is expected for pHis given the self-associative tendency of neutral imidazole groups near and above the p*K*_a_. The even slower recovery in the ATP-containing system is consistent with additional π-stacking between the adenine group and pHis imidazole rings further restricting chain mobility.

### 3.5 Kinetics of Esterase-Like Catalysis in a Changing pH Environment Driven by Urease

Given the strong dependence of reaction rate on pH, we wondered if an enzyme-mediated pH change could act as a switch for the hydrolysis reaction. We incorporated urease to increase pH (Figure 6a). Indeed, imaging during this reaction revealed an increase in product (CDF) fluorescence as well as the characteristic change in coacervate morphology that accompanies the pH increase (Figure 6b). As in the enzyme-free reaction at a static pH (Figure 5), the fluorescent product first appeared in the inner pHis-rich phase before diffusing to the surrounding phases of the multiphase droplets (Figure 6b,c and Supplementary Figure 33). When the reaction rate was monitored by bulk fluorescence spectroscopy of multiphase droplet suspensions, we found that the urease-containing system (initial pH = 4.7, increasing due to urease activity) exhibited kinetics comparable to the static system at pH 5.7 and ultimately reached the fastest rate of all (Figure 6d). Possible explanations for this enhanced rate include locally higher pH at early time points due to compartmentalization of urease within the pHis-rich inner phase, enhanced mixing arising from ammonia production during the urease reaction,^64,65^ and pH-driven multiphase reorganization that redistributes pHis interaction partners and reduces homotypic imidazole–imidazole π-stacking, as discussed above.

**Figure 6:**
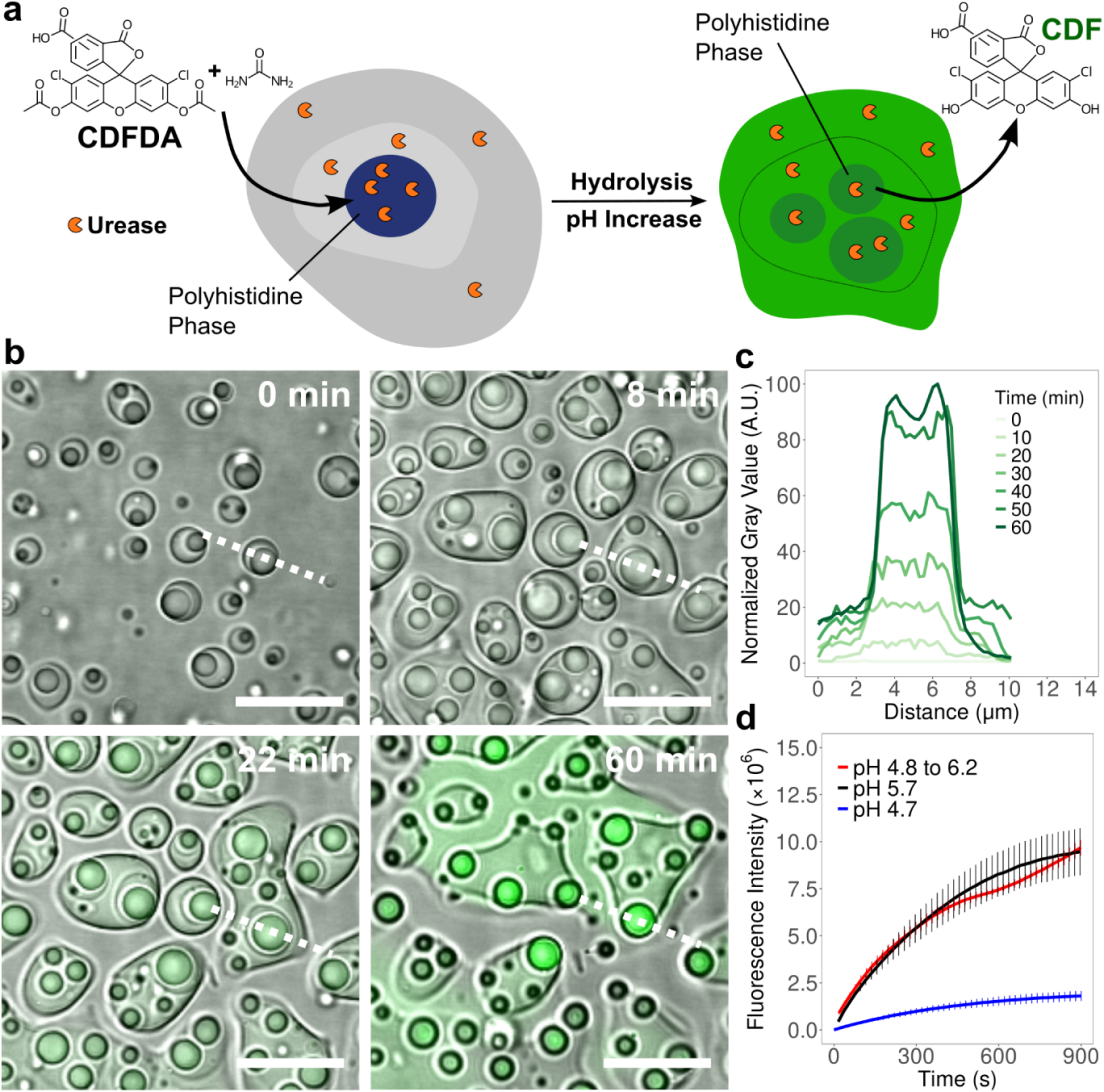
Kinetics of esterase-like catalysis in a changing pH environment. **a)** Schematic showing morphological changes and distribution of fluorescent product as the pH increases during the pH change and CDFDA hydrolysis in the pAsp multiphase. **b)** Merged confocal microscopy images of the transmitted light (DIC, grayscale) and CDF fluorescence (shown in green) channels showing structural changes in the coacervates as the pH increases and the hydrolysis of CDFDA into the green fluorescent dye (CDF) over a period of 60 min for the pAsp multiphase. **c)** Line profile plots showing the increase in fluorescence intensity across the multiphase over the course of the 60 min reaction (plots correspond to dashed lines in **b**. **d)** Fluorescence spectroscopy kinetics showing the hydrolysis of CDFDA in the pAsp multiphase at static pH values of 4.7 and 5.7 and in a pH-changing system from pH 4.8 to 6.2 over the course of 15 min. Error bars in **d** represent standard deviations of a minimum of 3 experiments under each set of conditions. Scale bars are 10 μm.

The reaction rate at a static pH of 4.7 (blue line) was found to be approximately 7× slower than both the enzyme-free system at pH 5.7 (black line) and the urease-mediated system, which reached a rate approximately 1.2× faster than the static system at pH 5.7 (red line, Figure 6d). Interestingly, the hydrolysis rate transiently decreased at approximately pH 5.8 before increasing further, producing an S-shaped kinetic profile (Figure 6d, Supplementary Figure 34). This pH corresponds approximately to the point at which the DEAE-dextran-rich phase appears when the pH is increased from 4.7 to 6 in earlier experiments (Figure 4 and Supplementary Video 1). As discussed above, ion pairing between pHis and polyanions within the coacervate environment can shift the apparent p*K*_a_ of pHis imidazoles relative to bulk solution,^54,57^ and the transient rate decrease at pH 5.8 likely reflects a reorganization of these interactions as the system crosses the effective p*K*_a_ of pHis within this multicomponent environment. The concurrent phase reorganization, which redistributes pHis interaction partners and transiently alters the accessibility of imidazole groups, may also contribute to the observed dip in rate before the system reaches its new equilibrium at higher pH.

While the esterase-like activity of the pHis-rich phase is clearly measurable and tunable via phase organization and pH, the catalytic efficiency remains modest compared to natural enzymatic systems. The substrate used here represents a model ester substrate, and the reaction is not optimized for maximal turnover number or substrate specificity. Rather than aiming to replicate enzymatic performance, the objective of this study is to demonstrate how multiphase coacervate architectures can modulate catalytic behavior through compartmentalization, phase hierarchy, and environmentally responsive reorganization. These findings illustrate principles of programmable microreactor design rather than maximal catalytic efficiency.

## 4 Conclusions

This work demonstrated that aggregation-prone hydrophobic polymers such as pHis can be stabilized against aggregation by incorporating them into a multiphase coacervate system. This is especially important as catalytic residues such as histidine typically function within buried, hydrophobic active sites that are shielded from the bulk aqueous environment by the surrounding protein scaffold, a property that is critical for their catalytic activity. In native enzymes, the active site is stabilized in solution by the surrounding protein structure. In our system, stabilization is instead achieved through multiple disordered coacervate phases, potentially offering a new strategy for supporting hydrophobic catalytic sites. Additionally, by integrating a pH-sensitive polymer like pHis we achieved pH-dependent reorganization of phases within the coacervate droplets near biological pH, with morphological and microenvironment changes influenced by the secondary structure of pHis and tunable through the choice of polyanion. Mechanistic analysis revealed that pH-dependent changes in pHis secondary structure, driven by the interplay between electrostatic repulsion and imidazole π-stacking, directly couple coacervate structure to catalytic activity. Counterintuitively, the multicompartment architecture enhanced the catalytic rate not only by providing a kinetic sink for product removal but also by suppressing homotypic imidazole self-association through competing heterotypic interactions, demonstrating that phase complexity can modulate the physical properties of the catalytic environment itself. Comparison of adenosine and its phosphorylated derivatives further showed that catalytic inhibition arises from cooperative electrostatic and π-associated contributions that can be partially deconvoluted, with the adenine base contributing independently of the phosphate groups through π-stacking interactions with pHis imidazoles. The pH of the system was controllable using urease or GOx enzymes, and the resulting morphological changes were fully reversible. Our findings build on prior work showing that reaction rates in aqueous two-phase systems can be enhanced by separating products and starting materials into different phases,^66,67^ but extend this principle by demonstrating dynamic modulation of polymer arrangement and phase composition through external stimuli.

Recent studies have shown that short peptides can form catalytic coacervates either by maintaining nonequilibrium droplet states through internal reactions, such as an aldol condensation reaction catalyzed by β-alanine amines,^68^ or by organizing flexible peptides into catalytically competent folded domains within self-coacervated droplets.^69^ These systems demonstrate that single-component peptide coacervates can couple compartmentalization and catalysis, but rely on homogeneous droplets with limited capacity to reorganize their internal structure in response to external cues. In contrast, our work demonstrates that the phase architecture itself actively modulates catalytic performance through two distinct mechanisms: the outer phases act as a kinetic sink that relieves product inhibition, while the multicomponent inner phase environment suppresses homotypic imidazole self-association, maintaining chain mobility and substrate accessibility under conditions where a single-phase system fails. Together, these features enable externally programmable, stimulus-responsive catalysis that cannot be replicated by adjusting composition or concentration within a single-phase system.

In addition, a key feature of our system is its use of histidine, a residue frequently found in enzymatic active sites including many that contain catalytic triads, where each amino acid is positioned with nanometer-level precision. Our research suggests that clustering catalytic groups at high, localized concentrations in a disordered system can still result in effective catalytic activity. A deeper understanding of the interplay between cation−π, π–π stacking, and electrostatic interactions within these systems will be critical for their further development. Substitution of ATP with pyrimidine-based nucleotides such as UTP or CTP, which retain triphosphate charge density but exhibit reduced π-stacking propensity relative to purines, represents a promising avenue for further disentangling these contributions and informing the rational design of future catalytic soft matter systems. Given the abundance of ester groups in synthetic polymer environmental pollutants,^70^ histidine-based multiphase systems could be used to degrade such chemicals in an eco-friendly way. In the long term, it may be possible to expand on the concept of histidine-based catalytic coacervates by incorporating additional reactive functional groups to further enhance catalytic efficiency or adjust substrate specificity, enabling the targeted breakdown of other compounds such as peptides, ethers, or phosphate-based nerve agents.

## Supplementary Material















## Notes

Detailed experimental methods including polyelectrolyte and enzyme fluorescent labeling, PEGylation of coverslips and capillaries, confocal microscopy and image analysis, and quantification procedures for fluorescence and Raman intensities; supplementary figures showing confocal microscopy, microRaman, circular dichroism, dynamic light scattering, and FRAP characterization of single-phase and multiphase coacervate systems, including pH-dependent phase behavior, component partitioning, enzyme-induced pH switching, and CDFDA hydrolysis kinetics; supplementary tables of Raman peak assignments, dynamic light scattering polydispersity indices, and CDFDA hydrolysis rate data (PDF)
Confocal microscopy video of DEAE-dextran (green channel)/CM-dextran, pLys (red channel)/pAsp, and pHis (blue channel)/pAsp multiphase coacervates undergoing an enzyme-induced pH change using urease enzyme (0.2 mg·mL^–1^) and urea (5 mM) as substrate, with a starting pH of around 4.7 and a final pH around 6. The arrows indicate the presence of the two separate pLys-rich phases (red) and the first appearance of the DEAE-dextran-rich phase (green) after approximately 3 s of the movie (6 min in real time). Total duration of the movie was 60 min (in real time), and the movie is played at 1 frame per second (AVI)
Confocal microscopy video of DEAE-dextran/CM-dextran (green channel), pLys (red channel)/pAsp, and pHis (blue channel)/pAsp multiphase coacervates undergoing an enzyme-induced pH change using GOx enzyme (0.2 mg·mL^–1^) and glucose (15 mM) as substrate, with a starting pH of around 5.7 and a final pH around 4.5. The arrows indicate the presence of DEAE-dextran-rich phase (green) at the start of the reaction, followed by its disappearance after approximately 12 s of the movie (24 min in real time) and the merging of small pHis-rich (blue) droplets as the pH decreases. Total duration of the movie was 60 min (in real time), and the movie is played at 1 frame per second (AVI)
Confocal microscopy video of DEAE-dextran/CM-dextran (green channel), pLys (red channel)/pAsp, and pHis (blue channel)/pAsp multiphase coacervates undergoing an enzyme induced pH change using urease enzyme (0.2 mg·mL^–1^) and urea (5 mM) as substrate, with a starting pH of around 4.7 and a final pH around 6. The arrows indicate the presence of a yellow phase that is rich in both CM-dextran (green) and pLys (red), this yellow phase is replaced by a green CM-dextran-rich phase after 2 s of the movie (4 min in real time), this green phase decreases in brightness (after 8 s of the movie, 16 min in real time) and finally coalesces to form a continuous phase after 23 s of the movie (46 min in real time). Total duration of the movie was 60 min (in real time) and the movie is played at 1 frame per second (AVI)
Confocal microscopy video of DEAE-dextran/CM-dextran (red channel), pLys/pAsp (green channel), and pHis (blue channel)/pAsp multiphase coacervates undergoing an enzyme induced pH change using urease enzyme (0.2 mg·mL^–1^) and urea (5 mM) as substrate, with a starting pH of around 4.7 and a final pH around 6. The arrows at the start of the movie indicate the pAsp-rich phase (green) and the pAsp and CM-dextran-rich phase (yellow). After 7 s of the movie (14 min in real time) the pAsp (green) and CM-dextran (red) separate into distinct phases, these phases disappear and reappear as a dilute mixture of pAsp (green) and CM-dextran (red) after 19 s of the movie (38 min in real time). Total duration of the movie was 60 min (in real time) and the movie is played at 1 frame per second (AVI)
Confocal microscopy video of DEAE-dextran/CM-dextran, pLys/pAsp, and pHis/pAsp multiphase coacervates catalyzing the hydrolysis of CDFDA (5 μM) substrate into the green fluorescent CDF product. The movie consists of an overlay of DIC and green channels. The arrows indicate the appearance of the green fluorescent CDF product in the center pHis-rich coacervate after 2 s of the movie (60 s in real time) followed by the later diffusion of the green product into the surrounding phases. Total duration of the movie was 15 min (in real time), and the movie is played at 1 frame per second (AVI)
Confocal microscopy video of DEAE-dextran/CM-dextran, pLys/pAsp, and pHis/pAsp multiphase coacervates simultaneously undergoing an enzyme induced pH change using urease (0.2 mg·mL^–1^) and urea (5 mM) and catalyzing the hydrolysis of CDFDA (20 μM) substrate into the green fluorescent CDF product. The movie consists of an overlay of the DIC and green channels and shows the formation of the green fluorescent CDF product and the change in morphology of the droplets as the pH changes. Arrows indicate the location of multiple phases in the DIC and the initial formation of green fluorescent CDF product in the center pHis-rich coacervate. product Total duration of the movie was 60 min (in real time) and the movie is played at 1 frame per second (AVI)
