# Supplementary material for: Dynamic and Catalytic Multiphase Coacervates
Source: Biomacromolecules. 2026 Jul 9;27(8):5090–104. doi: 10.1021/acs.biomac.5c02736 (PMC13463552; doi:10.1021/acs.biomac.5c02736)
Supplement: Supplementary file 1 [file bm5c02736_si_001.pdf]

Supplementary Information

Dynamic and Catalytic Multiphase Coacervates

Richard Booth*, Jiaqi Pei†, Jacob Shaffer† and Christine D. Keating*

Department of Chemistry, Pennsylvania State University, University Park, PA 16802.

† These authors contributed equally

*Address correspondence to: boothr@union.edu, keating@chem.psu.edu

Supplementary Methods 2

Materials and Methods 2

Fluorescent Labeling of Polyelectrolytes and Enzymes 2

Molecular Weights of Polyelectrolytes Used to Calculate Concentrations 3

PEGylation of Coverslips and Capillaries 3

Confocal Microscopy and Image Analysis 3

Quantification of Relative Fluorescent Intensities 4

Quantification of Relative Raman Intensities 4

Circular Dichroism Spectroscopy 4

Dynamic Light Scattering 5

Fluorescence Recovery After Photobleaching (FRAP) 5

Supplementary Figures 6

Supplementary Table 1. 9

Supplementary Table 2. 22

Supplementary Table 3. 27

Supplementary References 33

Supplementary Methods
Materials and Methods
All materials were used as received. Poly-L-histidine (pHis, average molecular weight 5,000-25,000 Da), diethylaminoethyl-dextran-fluorescein isothiocyanate (DEAE-dextran-FITC, average molecular weight 40,000 Da), sodium hydroxide, adenosine 5'-triphosphate disodium salt hydrate (ATP), adenosine 5'-diphosphate sodium salt (ADP), adenosine monophosphate (AMP), adenosine, 4-morpholineethanesulfonic acid (MES), acetic acid, sodium acetate, glucose, urea, dimethyl sulfoxide, *N*-(3-Dimethylaminopropyl)-*N′*-ethylcarbodiimide hydrochloride (EDC), 5(6)-Carboxy-2′,7′-dichlorofluorescein diacetate (CDFDA), 5(6)-Carboxy-2′,7′-dichlorofluorescein (CDF), sodium cyanoborohydride, glucose oxidase (≥ 100,000 units/g) and urease (50,000-100,000 units/g) were purchased from Sigma-Aldrich. Poly-L-lysine hydrochloride, (pLys, PDI = 1.00-1.20, average molecular weight 16,000 Da) and poly-L-aspartic acid (pAsp, 1.00-1.20, average molecular weight 11,500 Da) were purchased from Alamanda Polymers. Alexa Fluor 633 maleimide, Alexa Fluor 488 hydrazide, rhodamine NHS, silicon spacers (Press-to-Seal Silicone Isolator, with 20 mm internal diameter and 0.5 mm depth) and Pierce™ Protein Concentrators 3K and 5K molecular weight cut-offs (MWCO) (both ≤ 0.5 mL volume), Slide-A-Lyzer dialysis cassettes (3.5 kDa MWCO) and Nalgene Rapid-Flow PES filter (0.22 μm) were purchased from ThermoFisher Scientific. Carboxymethyl-dextran (CM-dextran) was purchased from TdB Labs (average molecular weight 40,000-60,000 Da). A custom peptide consisting of 20 histidine residues terminated by a cysteine residue on the C-terminus was purchased from GenScript (pHis20-C). Hydrochloric acid, ethanol, toluene, and micro cover glass slides for confocal experiments (no. 1.5, 24 × 30 mm) were purchased from VWR. *N*-(triethoxysilylpropyl)-o-polyethylene oxide urethane was purchased from Gelest. Bio-Gel P-6 fine polyacrylamide beads for size exclusion chromatography (100g, 45-90 μm, 1,000-6,000 MW fractionation range) were purchased from Bio-Rad Laboratories, Inc. Cover slides for microRaman (beveled edge, clipped corners, 25×75×1 mm, frosted 1 end, 1 side) were purchased from Globe Scientific Inc. Glass capillaries (Rectangle Boro Tubing 3520-050 Material / Length: Borosilicate / 050 mm) were purchased from VitroCom. All solutions were prepared using Milli-Q ultrapure water (18.2 MΩ·cm resistivity at 25 °C).

Fluorescent Labeling of Polyelectrolytes and Enzymes
Fluorescent variants of polyelectrolytes were synthesized using commercially available dyes via conjugation chemistry.[1] Labeled pLys was prepared using NHS ester chemistry: 1.2 mol equiv of dye per polymer molecule was added to a 5 mg·mL^{-1} solution of pLys in HEPES buffer (20 mM, pH 7.4). The reaction was stirred overnight and purified using a size-exclusion column and concentrated with a centrifugal filter (MWCO 5 kDa). pAsp and CM-dextran were labeled using hydrazide dyes. A 25× molar excess of EDC per polymer molecule was added to a 5 mg·mL^{-1} polymer solution in MilliQ water (adjusted to pH 5 with 1 mM HCl). After ~15 min, 1.2 equiv of hydrazide dye (for pAsp) or 5 equiv (for CM-dextran) were added, and the reaction was stirred overnight. The next morning, 5 equiv of sodium cyanoborohydride were added to stabilize the

hydrazone linkage. The labeled polymers were purified by size-exclusion chromatography and concentrated using centrifugal filters (MWCO 5 kDa). Instead of directly labeling pHis, a custom peptide consisting of 20 histidine residues with a C-terminal cysteine (pHis20-C) was used. Standard maleimide chemistry was used to label the Cys-terminated peptide. A 1.2 molar equivalent of maleimide dye was added to a 5 mg·mL^{-1} solution of the peptide in HEPES buffer (20 mM, pH 7.4). The labeled peptide was purified by dialysis (MWCO 3.5 kDa) and concentrated using centrifugal filters (MWCO 3 kDa). Urease and glucose oxidase were also labeled using the same NHS ester chemistry described above for pLys. All molecules were lyophilized prior to use and were stable for ~1 month at 4 °C, or were stored at –22 °C for long-term storage.

Molecular Weights of Polyelectrolytes Used to Calculate Concentrations

The molecular weight of the monomer was used when calculating the molecular weights of each polymer. The molecular weight of peptides was calculated using the amino acid molecular weight less one water as an approximation for the charge concentration (pHis = 137.15 Da, pAsp = 115.09 Da, pLys = 128.18 Da, all molecular weights are per monomer). The molecular weight used when making stock solutions for coacervation was always the monomer molecular weight and not the molecular weight of the full polymer. Glucose was used as the monomer molecular weight of DEAE-dextran and CM-dextran (180.16 Da) and the full molecular weight of ATP disodium salt hydrate was used (551.14 Da) for ATP.

PEGylation of Coverslips and Capillaries

Glass coverslips or capillaries were soaked for 30 min in a solution of ~1 M KOH in isopropanol, after which they were washed thoroughly with ethanol followed by Milli-Q water and dried in the oven at 60 °C overnight. Approximately 900 mg of *N*-(triethoxysilylpropyl)-o-polyethylene oxide urethane was dissolved in 300 mL of toluene. The washed glass was submersed in the toluene solution and left to react for 4 hr. The toluene solution was removed, and the glass was washed thoroughly with ethanol and then MilliQ water before being left in the oven to dry overnight at 60 °C before use.[2]

Confocal Microscopy and Image Analysis

Confocal images were acquired using a Leica TCS SP5 laser scanning confocal inverted microscope. Samples were prepared as described in the *Formation of Coacervates* section, with the addition of a small amount of fluorescently labeled polymer or its equivalent (pHis20-C) prior to the final mixing step to form the multiphase. The amount of labeled fluorophore added was adjusted for each system to ensure sufficient signal intensity while minimizing any potential effects on multiphase stability. Samples were imaged on custom-made poly(ethylene glycol)-functionalized coverslips to prevent coacervate wetting on the glass surface. All samples were prepared and imaged promptly after preparation. Fluorescence intensity values were obtained from raw images in Fiji following background correction. Droplets were counted using the Particle Analysis plugin in Fiji, with a circularity threshold of 0.5.

Quantification of Relative Fluorescent Intensities

Two normalization strategies were applied depending on the type of plot. For bar plots displaying a single fluorophore (one graph per dye), intensities were normalized to the maximum average intensity observed across all phases for that dye; this maximum was set to 100%, and all other values were scaled relative to it. This approach emphasizes the relative distribution of each fluorophore across coacervate phases. For plots combining all fluorophores and phases on a single graph, intensities were normalized to the maximum observed intensity across all fluorophores and phases (typically Alexa Fluor 633). This allowed direct comparison of fluorophore enrichment across phases, although weaker fluorophores may appear underrepresented when compared to the brightest dye. In all cases, normalization was based on ≥20 intensity measurements per phase, and error bars represent standard deviations. As fluorescence is influenced by the physicochemical properties of the local coacervate phase, we avoided using environment-sensitive rotor dyes;[3] however, phase-dependent intensity effects cannot be fully excluded.

Quantification of Relative Raman Intensities

To analyze ATP partitioning, we quantified the 719 cm^{-1} Raman band corresponding to phosphate stretching vibrations.[4-6] MicroRaman maps were converted to grayscale and analyzed in Fiji. For each droplet, a line was drawn across the image from the inner coacervate phase to the surrounding dilute phase, and average signal intensities were extracted for each phase along the line scan. Intensities were then normalized by setting the sum of signals from all phases within the droplet to 100%, and expressing the signal from each phase as a fraction of this total. This procedure was repeated for all imaged droplets (n > 10).

Circular Dichroism Spectroscopy

Poly-L histidine (50 mM monomer concentration) was dissolved in Milli-Q water and pH adjusted to ~pH 4 (verified Supelco pH-indicator strips pH 2.0-9.0) with 2 M HCl to fully solubilize. Stocks were then pH adjusted to 4.5 and 6.0 with NaOH for analysis (measured by pH probe). CD spectra were collected using a Jasco J-1500 CD Spectrophotometer, scanning at 50 nm/min with a 4-second data integration time in a 0.1mm pathlength cuvette. Samples were collected in triplicate with 3 acquisitions per sample. Samples were prepared by diluting pHis at the appropriate pH in Milli-Q water. pH test strips were used to verify pH after dilution. For samples containing ATP/pAsp, ATP and pAsp stocks were individually pH adjusted to 4.5 and 6.0 prior to use. pHis was diluted in the appropriate amount of water followed by addition of ATP/pAsp at the correct pH.

Data was normalized to the mean residue ellipticity (MRE) by the formula:

$$MRE = \frac{MRW \times \theta}{10 \times d \times c}$$

Where MRW = mean residue weight = 137.146 Da = MW of histidine – 18.0 Da (water) because only histidine residues are present, θ = observed ellipticity (milidegrees), d = pathlength (cm), c = concentration (mg.mL^{-1}) to give the final units of deg × cm^2 × dmol^{-1}.

Dynamic Light Scattering

Dynamic light scattering (DLS) data were collected on a Wyatt Dynapro Nanostar DLS detector. Mili-Q water, NaOH, and HCl used for stock and sample preparation were filtered through a 0.22 μm filter (Thermo Scientific Nalgene Rapid-Flow PES filter). pHis stocks were prepared in effort to avoid pH dependent pHis aggregation; pHis was dissolved in filtered Mili-Q water and pH adjusted to pH 4.0-4.5, verified with pH test strips. pHis, ATP, and pAsp stocks were filtered with 0.22μm syringe filters (Millipore) and the pH of each was adjusted to pH 4.5 or 6.0, verified via pH probe. Sample components were added in fixed order; pHis was diluted with an appropriate volume of water, followed by the addition of pAsp or ATP (except in no polyanion trials). Samples were gently mixed and immediately transferred to cuvettes for measurement. The polydispersity index (PDI) was calculated via two methods. The cumulant fit from the autocorrelation function was calculated by the instrument at data acquisition and provides the overall PDI of each system. Each distribution was also individually fit to a Gaussian function and the PDI is reported as σ^2/r^2 where σ is the standard deviation and r is the mean radius of each distribution.

Fluorescence Recovery After Photobleaching (FRAP)

FRAP experiments were performed on pHis-rich phases doped with fluorescently labeled pHis20-C peptide conjugated to Alexa Fluor 633. We used the same image settings as for the previous experiments. A 10-frame pre-bleach sequence was followed by a 10-frame bleach at 100% 633 nm, 100% 543 nm, and 10% 488 nm laser powers. Regions of interest were 1 μm in diameter, and droplets were imaged with a 1400 Hz scanning speed. For accurate comparison across droplets, we selected coacervates that were approximately the same size and, where possible, ensured that the droplet diameter was larger than the bleached region; however, this was challenging due to the small size of many multiphase coacervates. FRAP curves were fitted to a single exponential recovery function in R software based on the following equation:[7]

$$N(t) = A\,(1 - \exp\,(-t\,/\tau)) + C$$

Where A and C are constants, and τ is the fluorescence recovery time. The halftime of recovery, $t_{1/2}$, is calculated using $\tau_{1/2} = \tau \ln(2)$.

Supplementary Figures

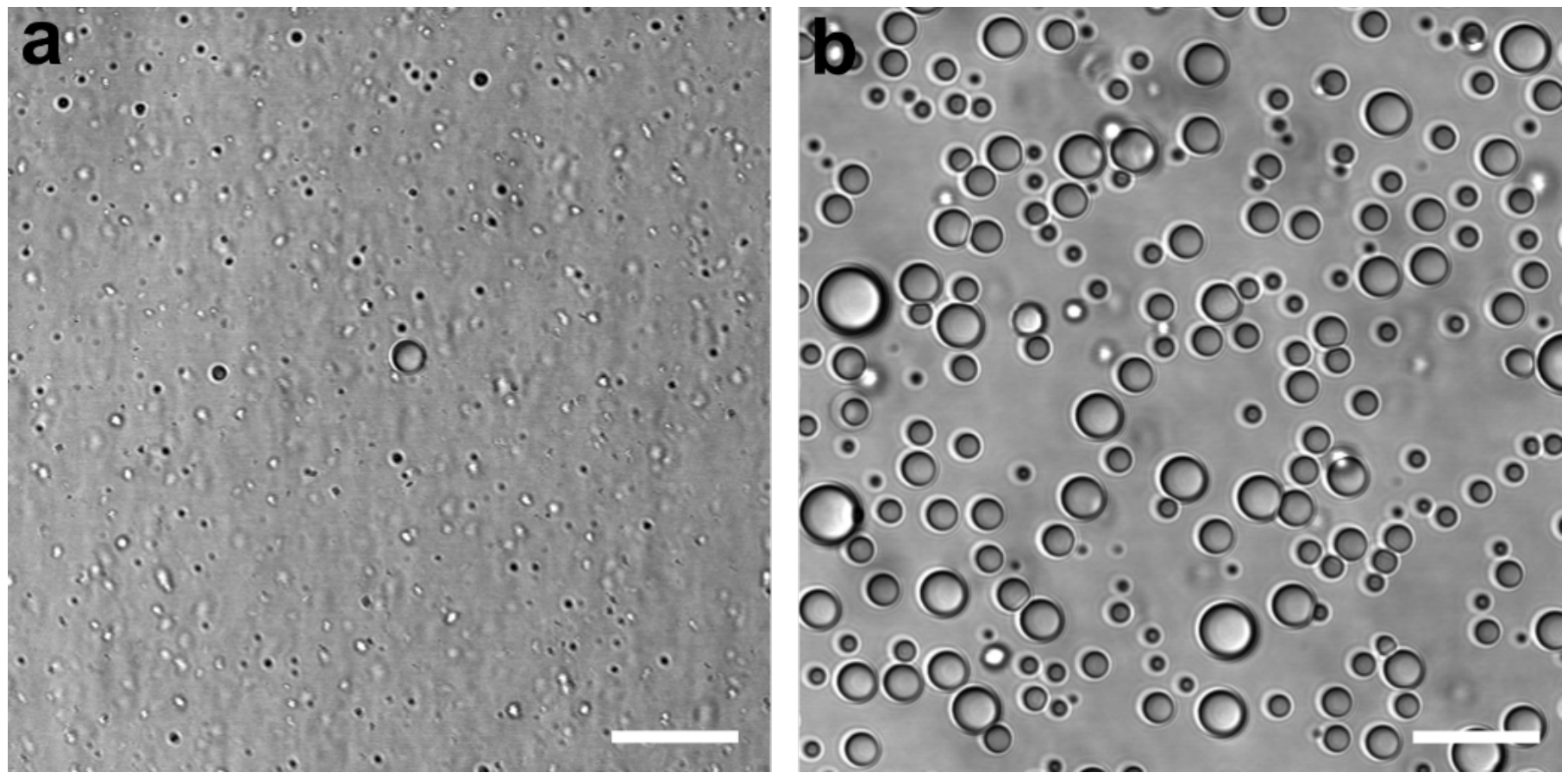

Supplementary Figure 1. (a) Brightfield images of pHis coacervates showing changes in droplet diameter at pH 6, when paired with pAsp **(a)** or ATP **(b)**. All polymers were used at a final monomer charge concentration of 10 mM; ATP was also used at a final molecular concentration of 10 mM. Scale bar: **(a)** = 20 µm, **(b)** = 10 µm.

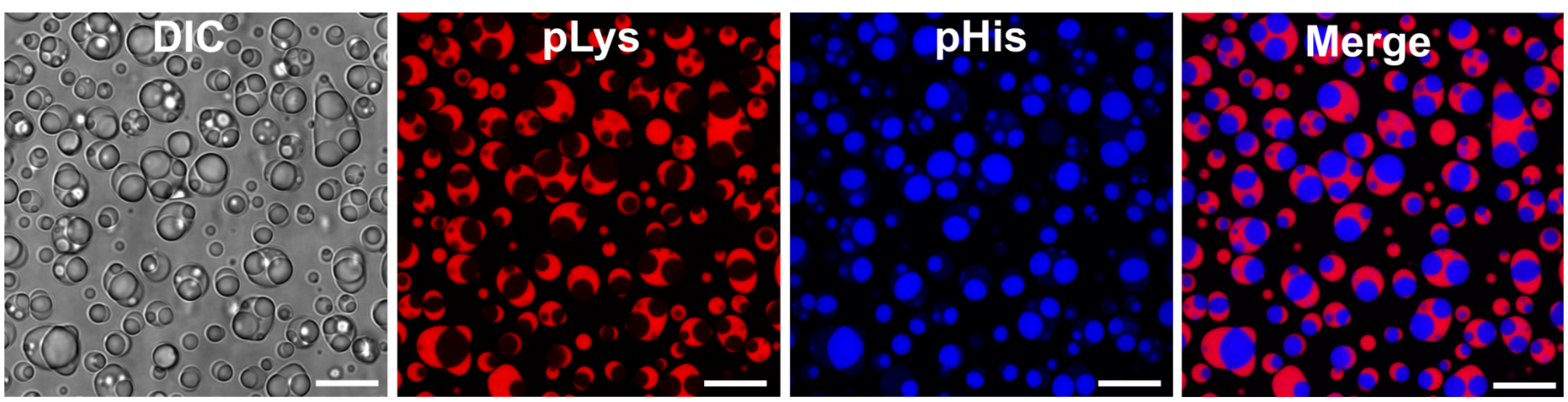

Supplementary Figure 2. Fluorescence confocal microscopy images of pHis/ATP and pLys/pAsp multiphase coacervates at pH 5.7. Single-phase coacervates were prepared separately before mixing together. Separate channels were false-colored for clarity. All scale bars = 10 µm.

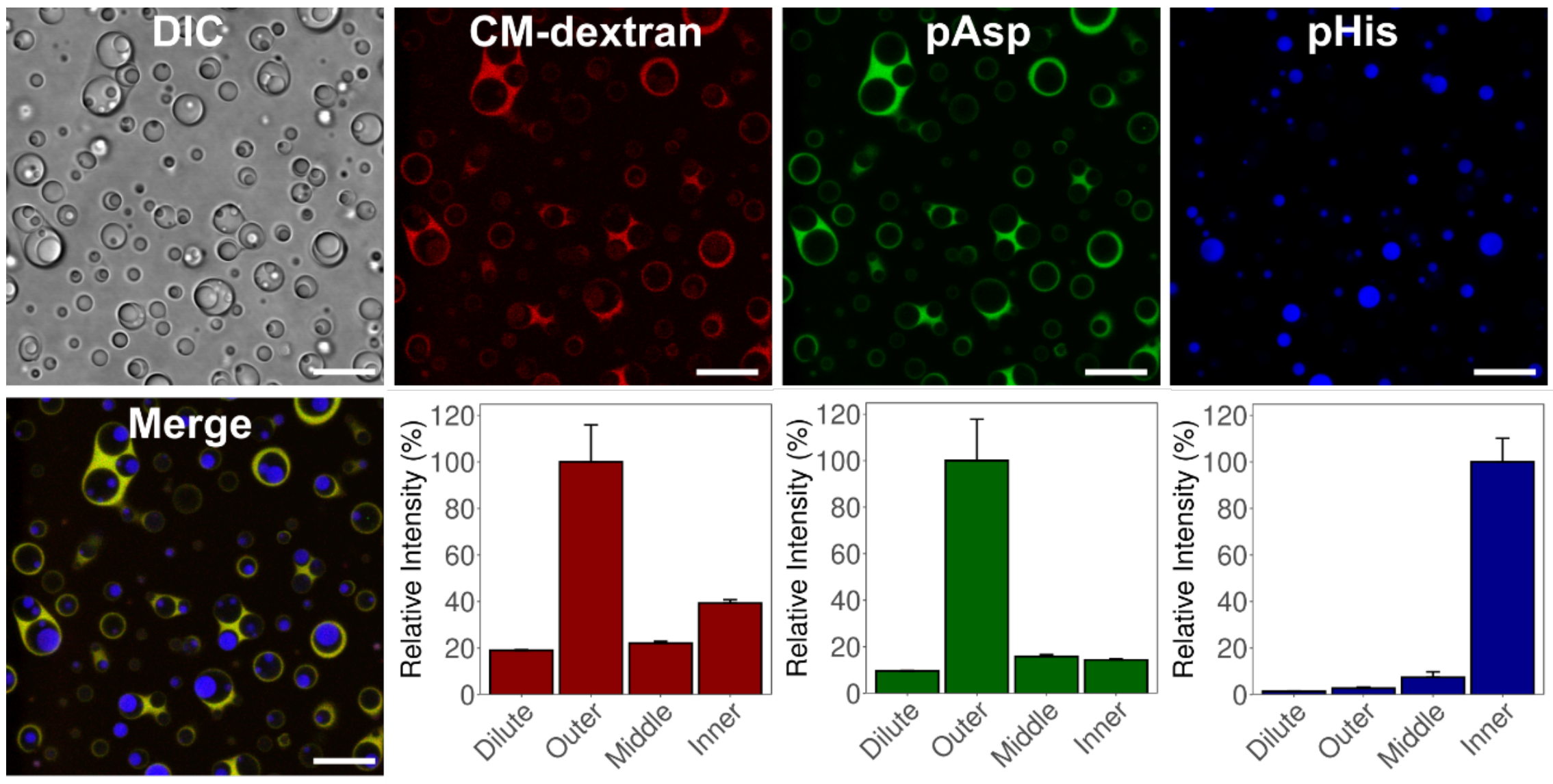

Supplementary Figure 3. Confocal microscopy images and corresponding relative intensity bar graphs showing the distribution of labeled polymers in ATP multiphase coacervates, highlighting the relative localization of polyanions and pHis at pH 5.7. The multiphase system was prepared by separately forming DEAE-dextran/CM-dextran, pLys/pAsp, and pHis/ATP coacervates prior to mixing them together. Labels in the figure correspond to the fluorescently labeled phases: CM-dextran was labeled with rhodamine, pAsp with Alexa Fluor 488, and pHis with Alexa Fluor 633. Bar plots display the relative fluorescence intensity in each phase, where the fluorescence intensities are normalized to the maximum signal for each dye, such that the phase with the highest intensity is set to 100%. Intensities in the other phases are expressed as a percentage relative to this maximum. Error bars represent standard deviations of a minimum of 20 droplets. Separate fluorescence channels were false-colored for clarity. Scale bars: 10 μm.

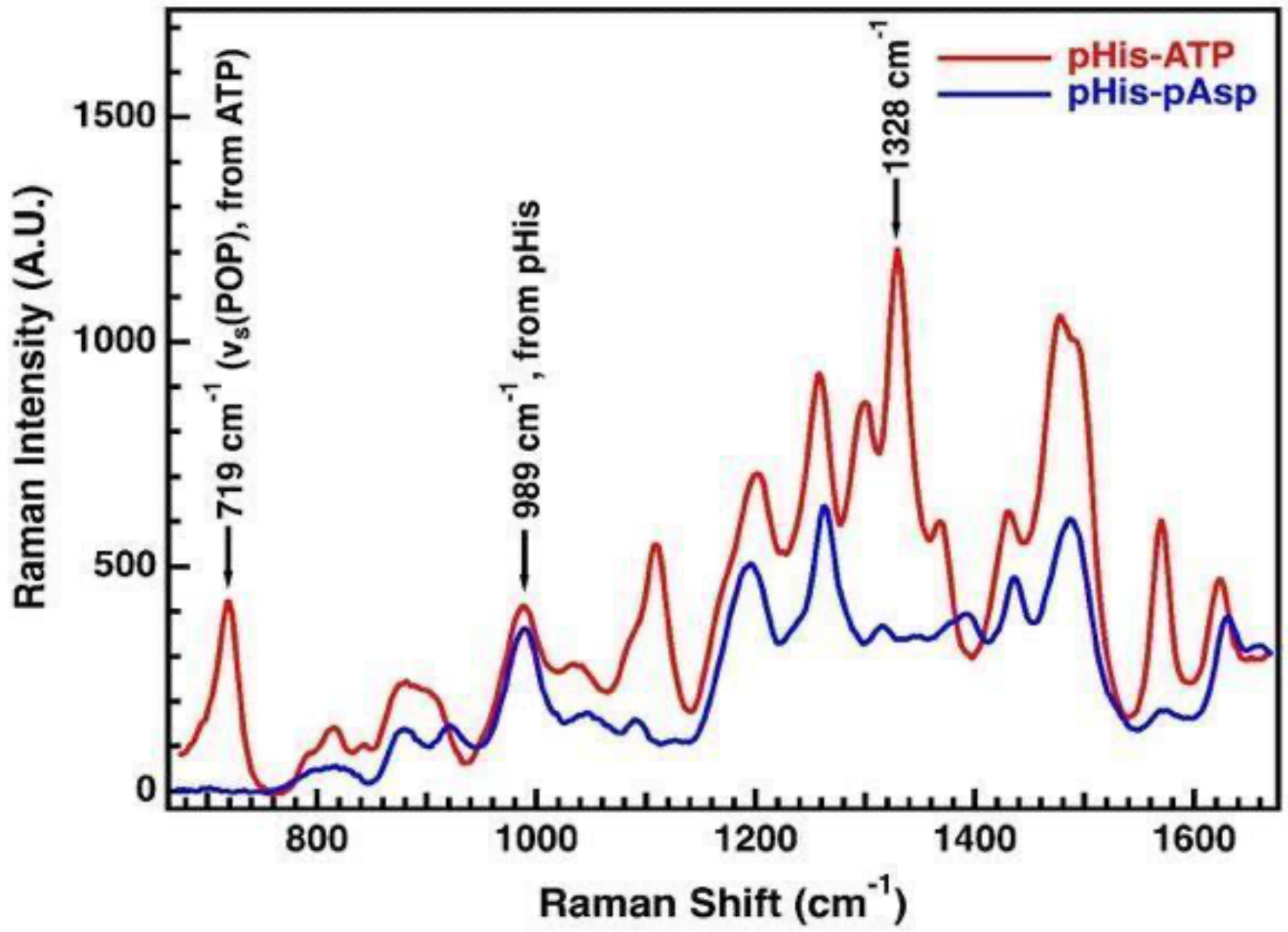

Supplementary Figure 4. Raman spectra of pHis/ATP (red) and pHis/pAsp (blue) single-phase coacervates, collected in MES buffer at pH 6. Peaks correspond to vibrational modes of the respective components in the condensed phase. For detailed peak assignments and experimental conditions, see *Supplementary Table 1* and the *Materials and Methods* section.

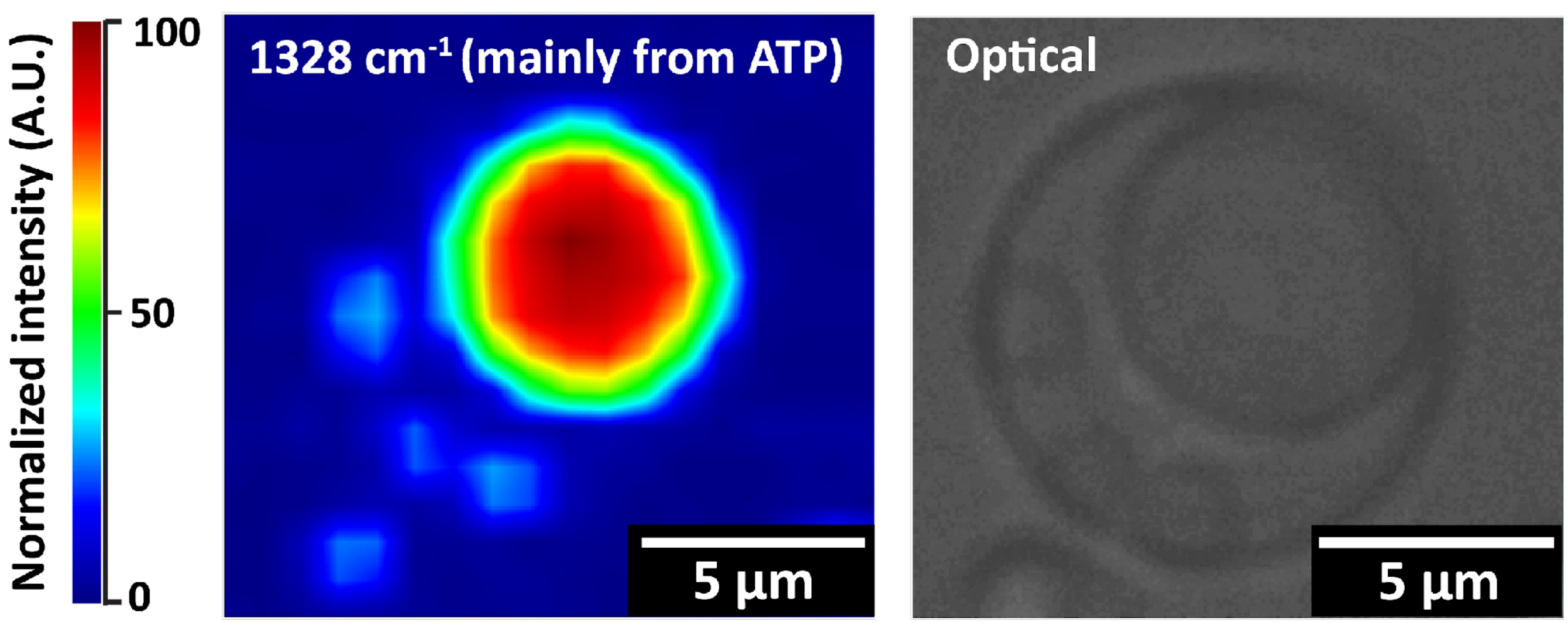

Supplementary Figure 5. microRaman map and corresponding optical image of a DEAE-dextran/CM-dextran, pLys/pAsp, and pHis/ATP multiphase coacervate droplet in MES buffer at pH 6. Raman intensities from the 719 cm^{-1} phosphate stretching band of ATP were analyzed in Fiji and normalized by setting the total signal across all phases to 100%. For detailed experimental conditions, see the *Materials and Methods* section.

Peak (cm^{-1})	Assignment
719	ATP: v_s(POP) triphosphate backbone stretching[4-6]
809	pHis pH>6.5: out of plane bending of C-H, C-C in imidazole[8] and ATP: v_{as}(POP) triphosphate backbone stretching[9]
848	ATP: adenine (skeletal mode, in-plane)[10]
878	pHis pH>6.5 (minor) and ATP: (minor): δ (N-9-H) out-of-plane[8-10]
989	pHis: imidazole ring C-C rocking or scissoring: δ (R)[8]
1043	pHis: side-chain skeletal stretching[11,12]
1108	ATP (major) and pHis pH>2.58 (minor): v_e (PO_3) O=P=O stretching, C-H in-plane bend[11]
1203	pHis pH=2.58 (minor) and pHis>6.5: NCN sym. str. + N-H in-plane bend[11,13]
1257	pHis: ring breath[11,13]
1299	ATP and pHis pH>6.5: ring breath[11]
1328	ATP (major): v(C-N), v(C=N); pHis pH>6.5 (weak): δ (R)[10,14]
1368	ATP: δ (C-8-H), δ (C-2-H) out-of-plane,[8] and pHis ring breathing[8,13]
1434	pHis methylene scissoring: δ (CH_2)[12]
1474	pHis pH=2.58 (minor) and ATP (major)[6,15]: N-H in-plane bend[8]
1494	pHis pH=2.58 (major) and ATP (minor)[6,15]: N-H in-plane bend[8]
1568	pHis pH=2.58 (minor) and ATP (major)[6,15]: C=C stretching[8]
1623	pHis pH=2.58: C=C stretching[11]
1664	pHis pH>6.5 (major) and pHis pH=2.58 (minor), amide I stretching[8,11]

Supplementary Table 1.

Observed peaks and their respective assignments from microRaman microscopy experiments.

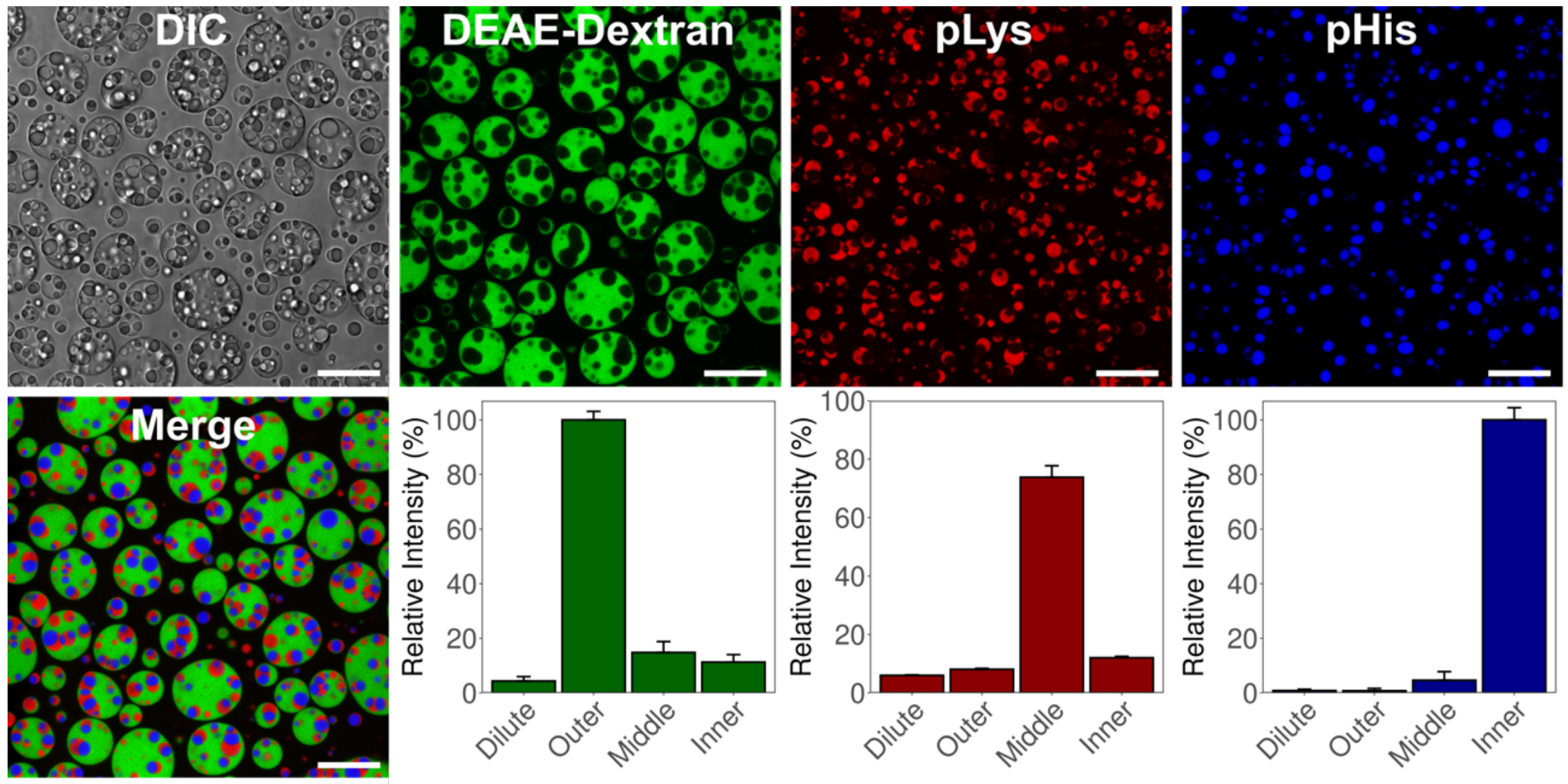

Supplementary Figure 6. Confocal microscopy images and corresponding relative intensity bar graphs showing the distribution of labeled polymers in multiphase coacervates, highlighting the relative localization of polycations and pHis at pH 4.7. The multiphase system was prepared by separately forming DEAE-dextran/CM-dextran, pLys/pAsp, and pHis/ATP coacervates prior to mixing them together. Labels in the figure correspond to the fluorescently labeled phases: DEAE-dextran was labeled with fluorescein, pLys with rhodamine, and pHis with Alexa Fluor 633. Bar plots display the relative fluorescence intensity in each phase, where the fluorescence intensities are normalized to the maximum signal for each dye, such that the phase with the highest intensity is set to 100%. Intensities in the other phases are expressed as a percentage relative to this maximum. Error bars represent standard deviations of a minimum of 20 droplets. Separate fluorescence channels were false-colored for clarity. Scale bars: 10 µm.

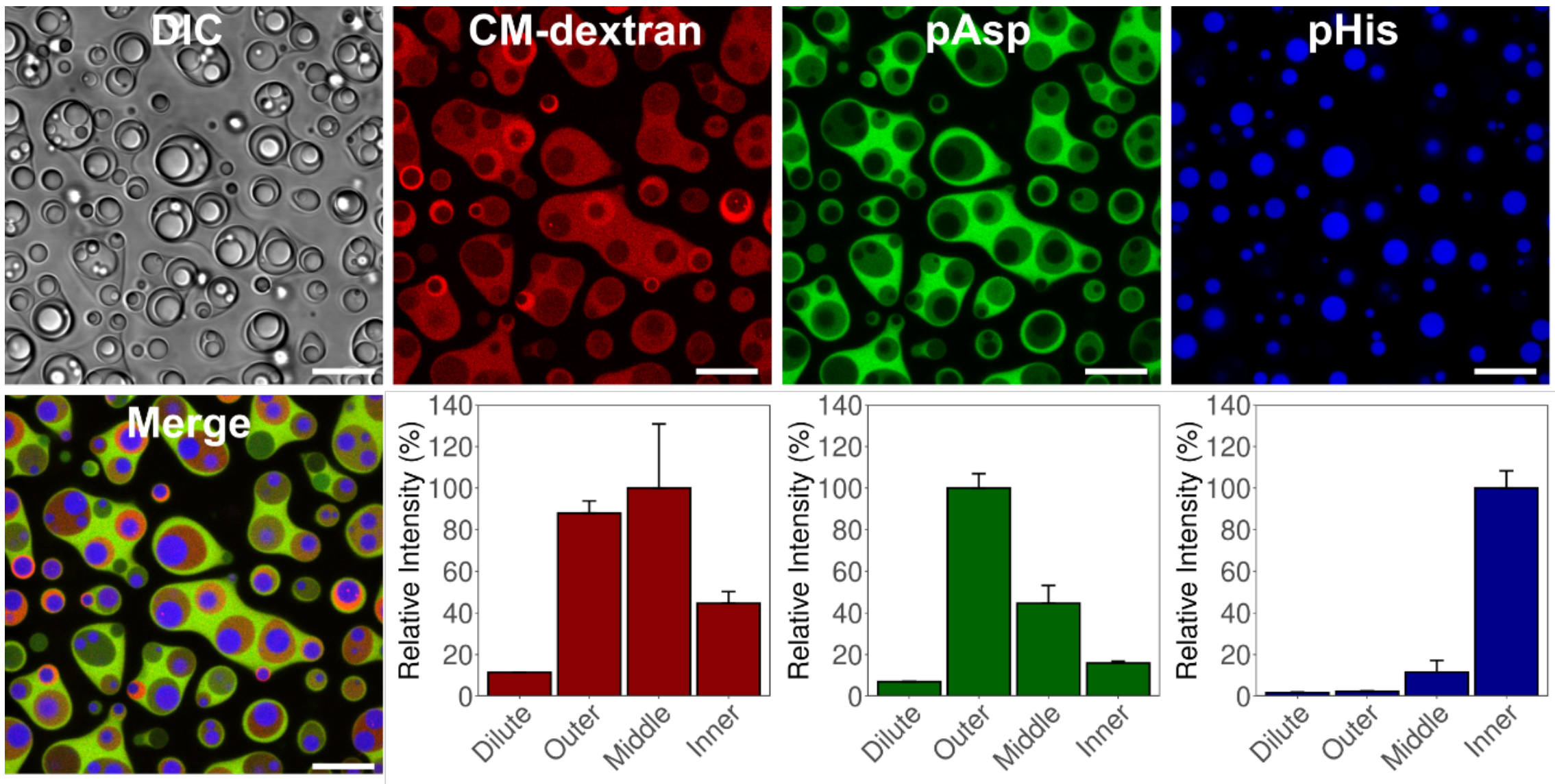

Supplementary Figure 7. Confocal microscopy images and corresponding relative intensity bar graphs showing the distribution of labeled polymers in multiphase coacervates, highlighting the relative localization of polyanions and pHis at pH 4.5 in the presence of ATP. The multiphase system was prepared by separately forming DEAE-dextran/CM-dextran, pLys/pAsp, and pHis/ATP coacervates prior to mixing them together. Labels in the figure correspond to the fluorescently labeled phases: CM-dextran was labeled with rhodamine, pAsp with Alexa Fluor 488, and pHis with Alexa Fluor 633. Bar plots display the relative fluorescence intensity in each phase, where the fluorescence intensities are normalized to the maximum signal for each dye, such that the phase with the highest intensity is set to 100%. Intensities in the other phases are expressed as a percentage relative to this maximum. Error bars represent standard deviations. Separate fluorescence channels were false-colored for clarity. Scale bars: 10 µm.

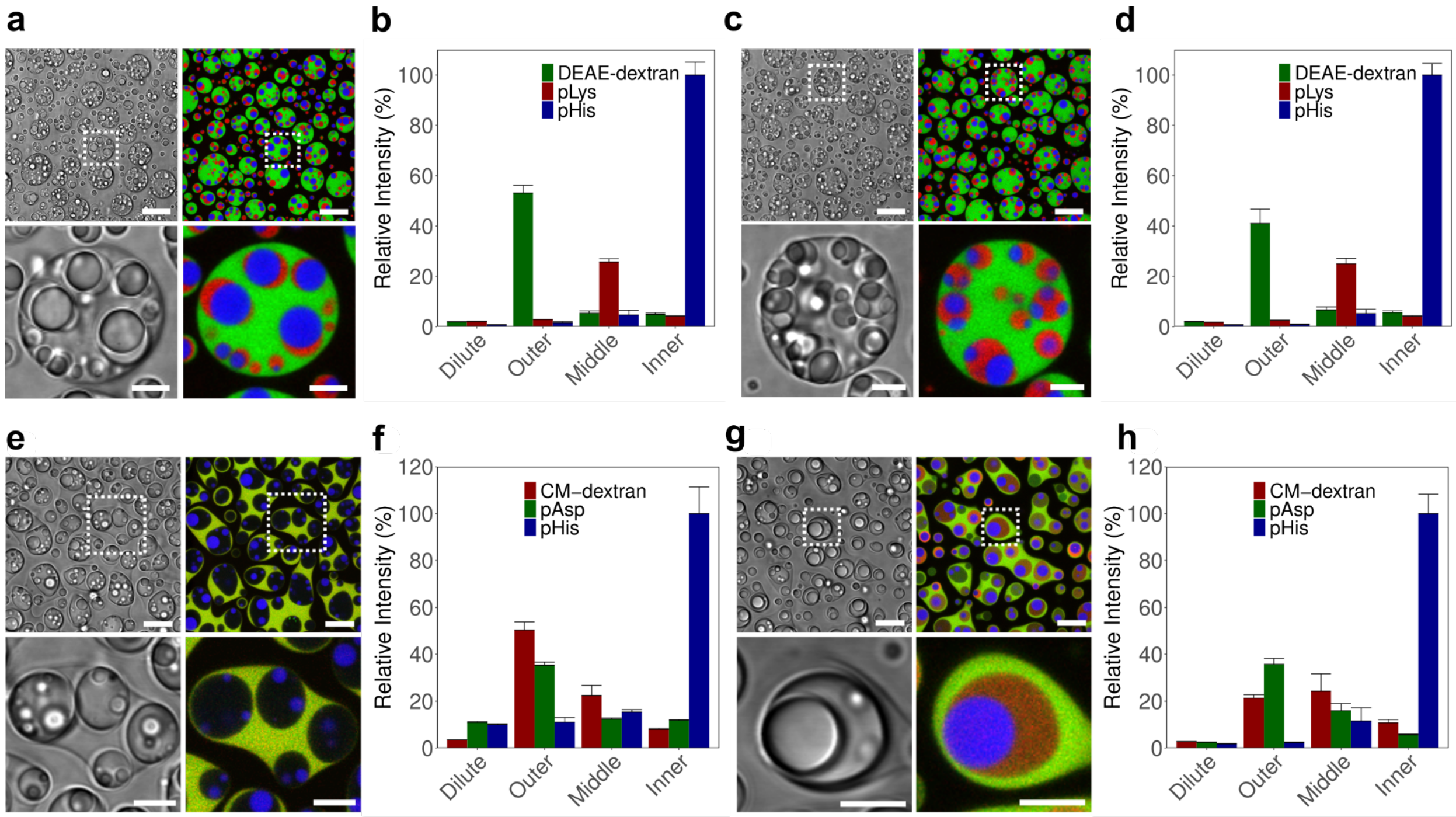

Supplementary Figure 8. Polycation and polyanion distribution in the ATP multiphase system at pH 5.7 and pH 4.7. (**a**, **c**) Confocal fluorescence microscopy images showing the distribution of labeled polycations in DEAE-dextran/CM-dextran, pLys/pAsp, pHis/ATP multiphase coacervates at pH 5.7 (**a**) and pH 4.7 (**c**). (**b**, **d**) Corresponding bar plots showing the relative fluorescence intensities of DEAE-dextran-FITC (green), pLys-rhodamine (red), and pHis-Alexa Fluor 633 (blue) across phases at pH 5.7 (**b**) and pH 4.7 (**d**). (**e**, **g**) Confocal fluorescence microscopy images showing the distribution of labeled polyanions in the same multiphase system at pH 5.7 (**e**) and pH 4.7 (**g**). (**f**, **h**) Corresponding bar plots showing the relative fluorescence intensities of the labeled polyanions across phases at pH 5.7 (**f**) and pH 4.7 (**h**). Intensities are normalized such that 100% corresponds to the maximum intensity across all dyes and all phases, with all other values scaled relative to this maximum. A minimum of 20 measurements were collected per phase. Error bars represent standard deviations. Scale bars: 10 µm (main images), 5 µm (magnified images).

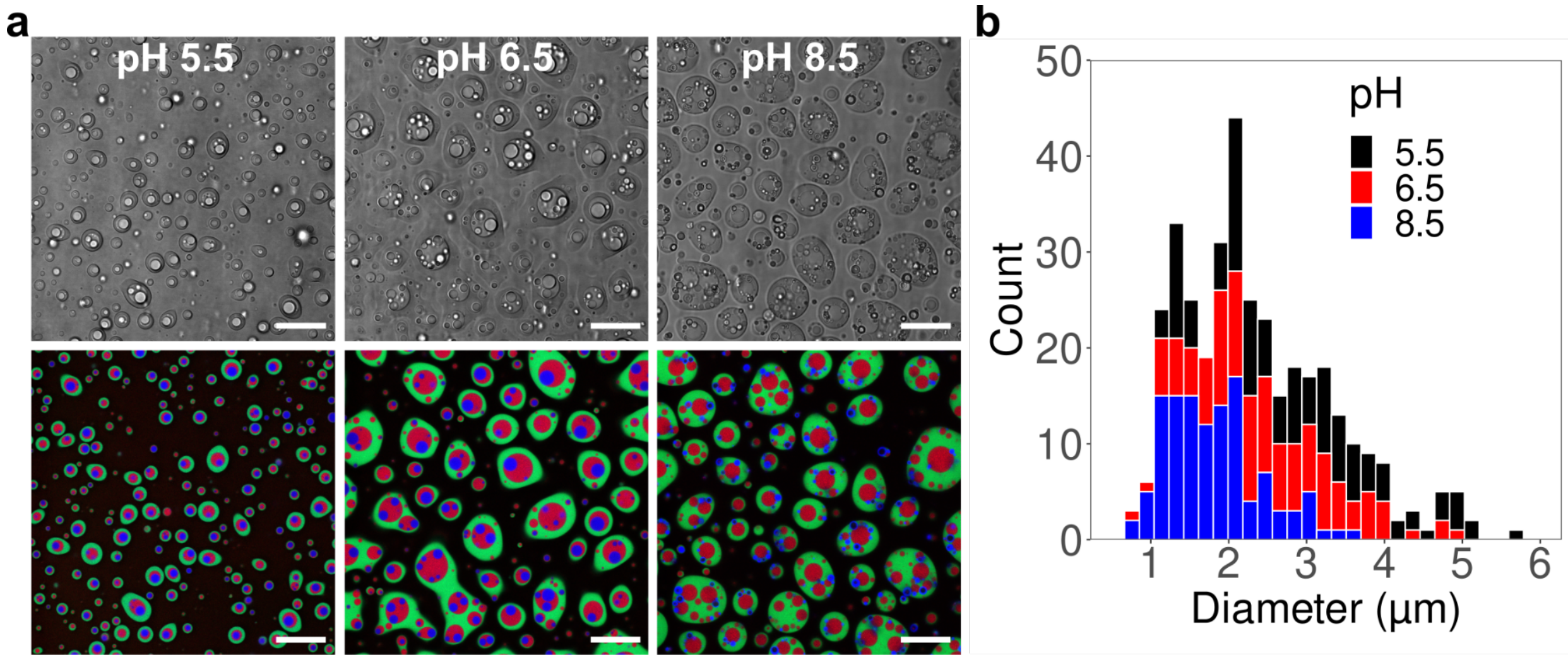

Supplementary Figure 9. Effect of pH on ATP-containing multiphase droplets. **(a)** Confocal microscopy images of DEAE-dextran/CM-dextran, pLys/pAsp, and pHis/ATP coacervates at pH 5.5, 6.5, and 8.5. DEAE-dextran was labeled with fluorescein (green), pLys with rhodamine (red), and pHis with Alexa Fluor 633 (blue). Separate fluorescence channels were false-colored for clarity. Scale bars: 10 µm. **(b)** Histograms of the diameters of the blue pHis-rich phase, based on 120 measurements at each pH. pH 5.5: mean = 2.79 µm, median = 2.69 µm; pH 6.5: mean = 2.48 µm, median = 2.35 µm; pH 8.5: mean = 1.81 µm, median = 1.74 µm.

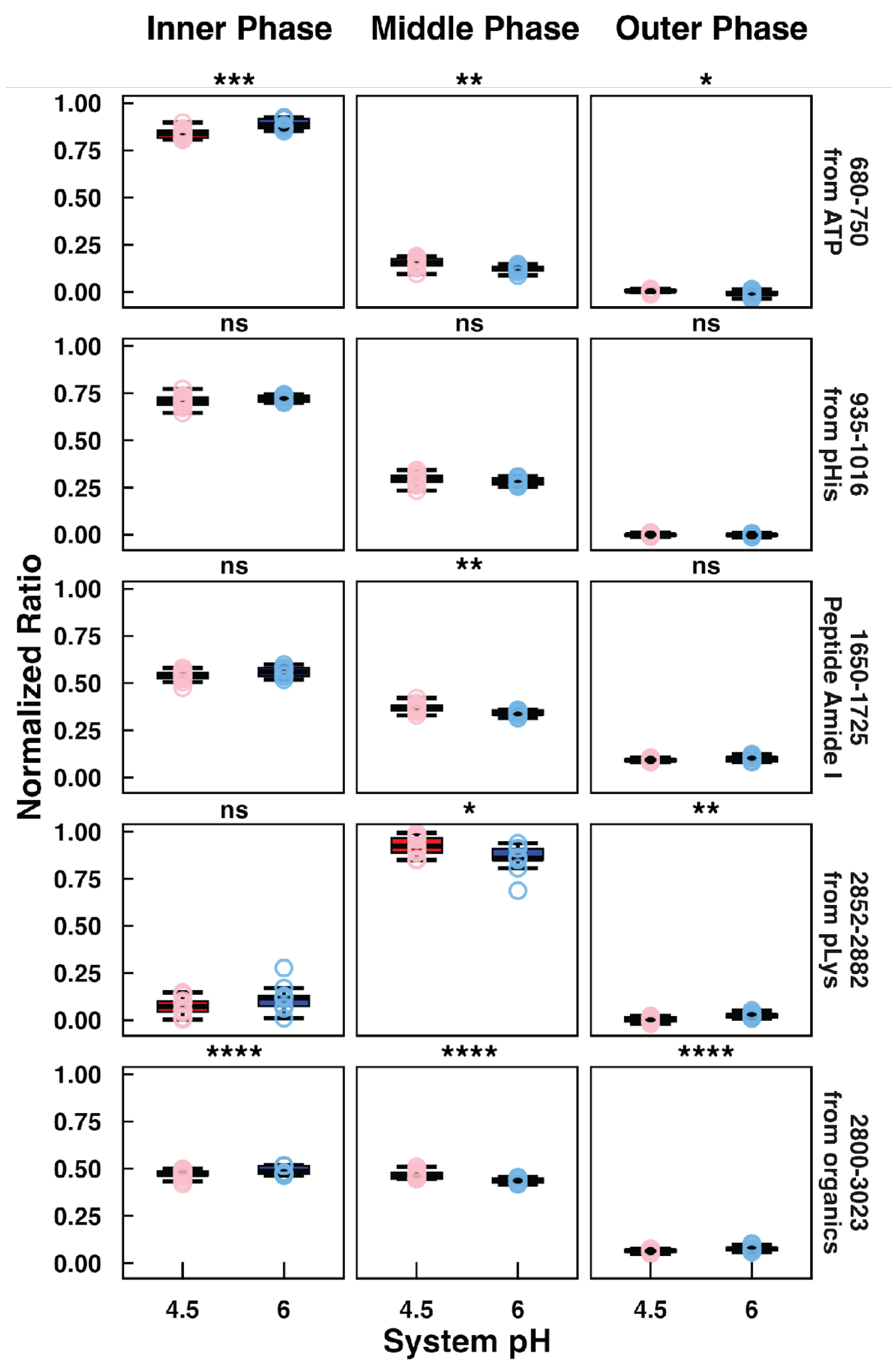

Supplementary Figure 10. Distribution of coacervate-forming components among different phases in pHis/ATP-pLys/pAsp-DEAE Dextran/CM Dextran multiphase coacervates. The spectra were baselined before integration. Within the sample, the area under the curve (AUC) was normalized by the sum of the AUC for the same peak range across different phases. The area of 680-750 cm^{-1} and 2852-2882 cm^{-1} was calculated between the curve and a straight line, which was drawn to connect the left and right borders of the peak. The rest of the AUC was calculated between the curve and the y = 0 line. Error bars represent standard deviations of 10 droplets among triplicates. The microRaman was not able to distinguish between the dextran coacervate phase and the dilute phase (all denoted as outer phases) due to low concentrations of Raman-active functional groups in these phases. T-test results were noted on top of each panel (ns: no statistical difference; *: $0.01 < p \leq 0.05$; **: $0.001 < p \leq 0.01$; ***: $0.0001 < p \leq 0.001$; ****: $p \leq 0.000$).

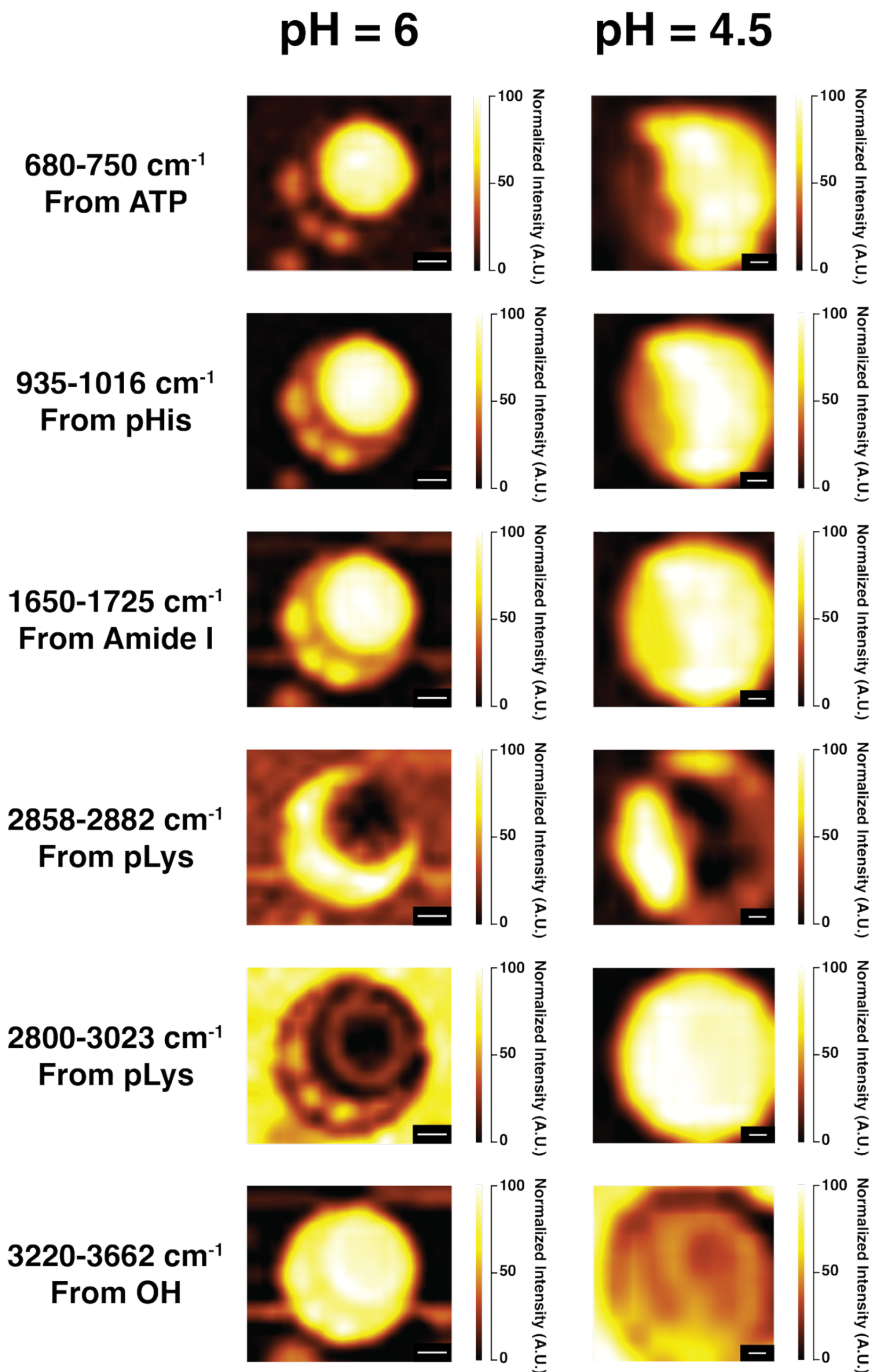

Supplementary Figure 11. MicroRaman maps of representative droplets at pH 4.5 and pH 6.0, plotted by characteristic peak and normalized to peak area. Scale bars: 1 μm (pH 4.5) and 2 μm (pH 6.0).

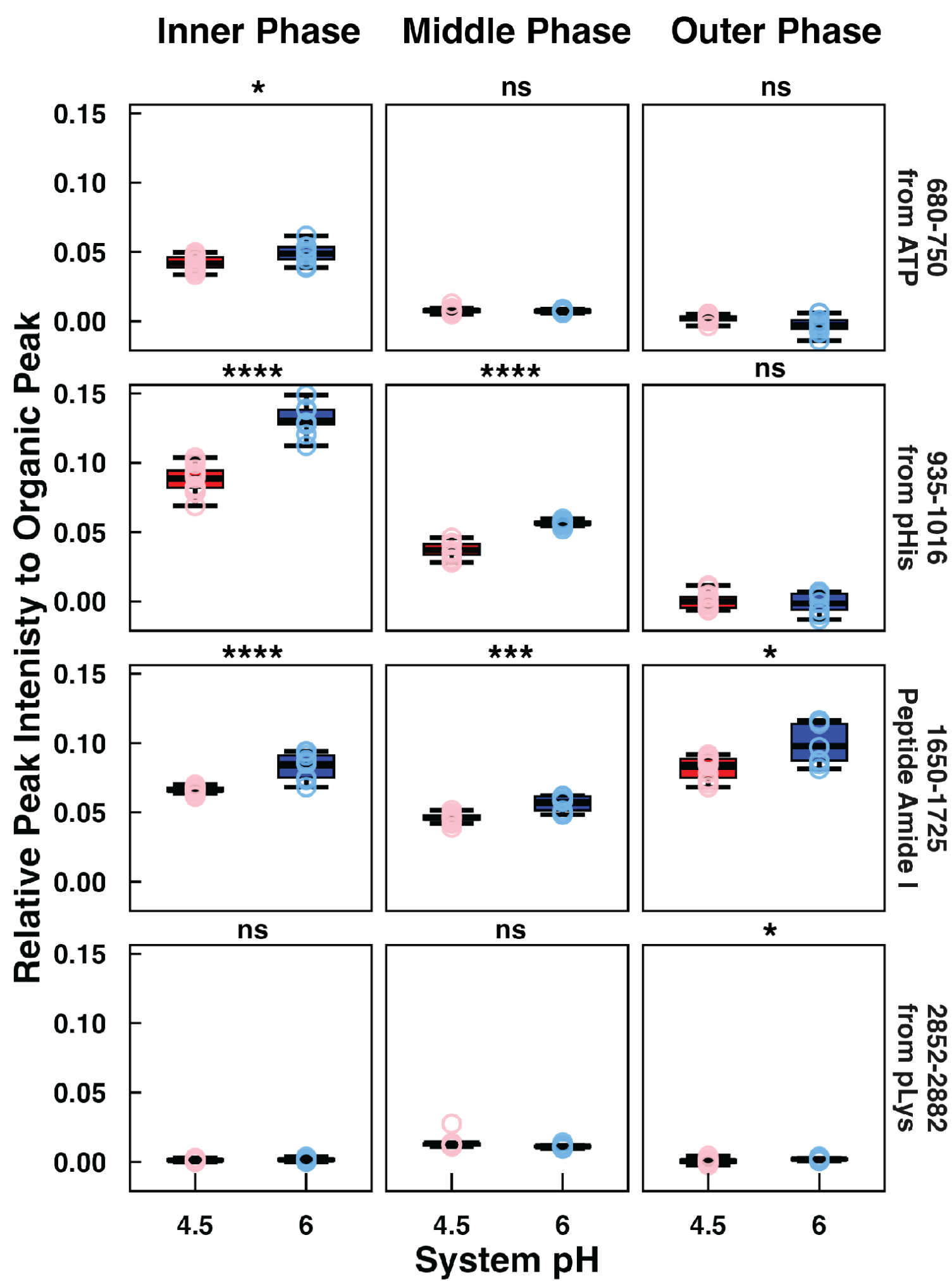

Supplementary Figure 12. Ratio of coacervate-forming component to overall organic matter in each phase of pHis/ATP-pLys/pAsp-DEAE Dextran/CM Dextran multiphase coacervates. The spectra were baselined before integration. Within the spectrum, the AUC was divided by the overall organic C-H stretching peak's AUC (2800-3023 cm^{-1}). The area of 680-750 cm^{-1} and 2852-2882 cm^{-1} was calculated between the curve and a straight line, which was drawn to connect the left and right borders of the peak. The rest of the AUC was calculated between the curve and the y = 0 line. Error bars represent standard deviations of 10 droplets among triplicates. The microRaman was not able to distinguish between the dextran coacervate phase and the dilute phase (all denoted as outer phases) due to low concentrations of Raman-active functional groups in these phases. T-test results were noted on top of each panel (ns: no statistical difference; *: $0.01 < p \leq 0.05$; **: $0.001 < p \leq 0.01$; ***: $0.0001 < p \leq 0.001$; ****: $p \leq 0.000$).

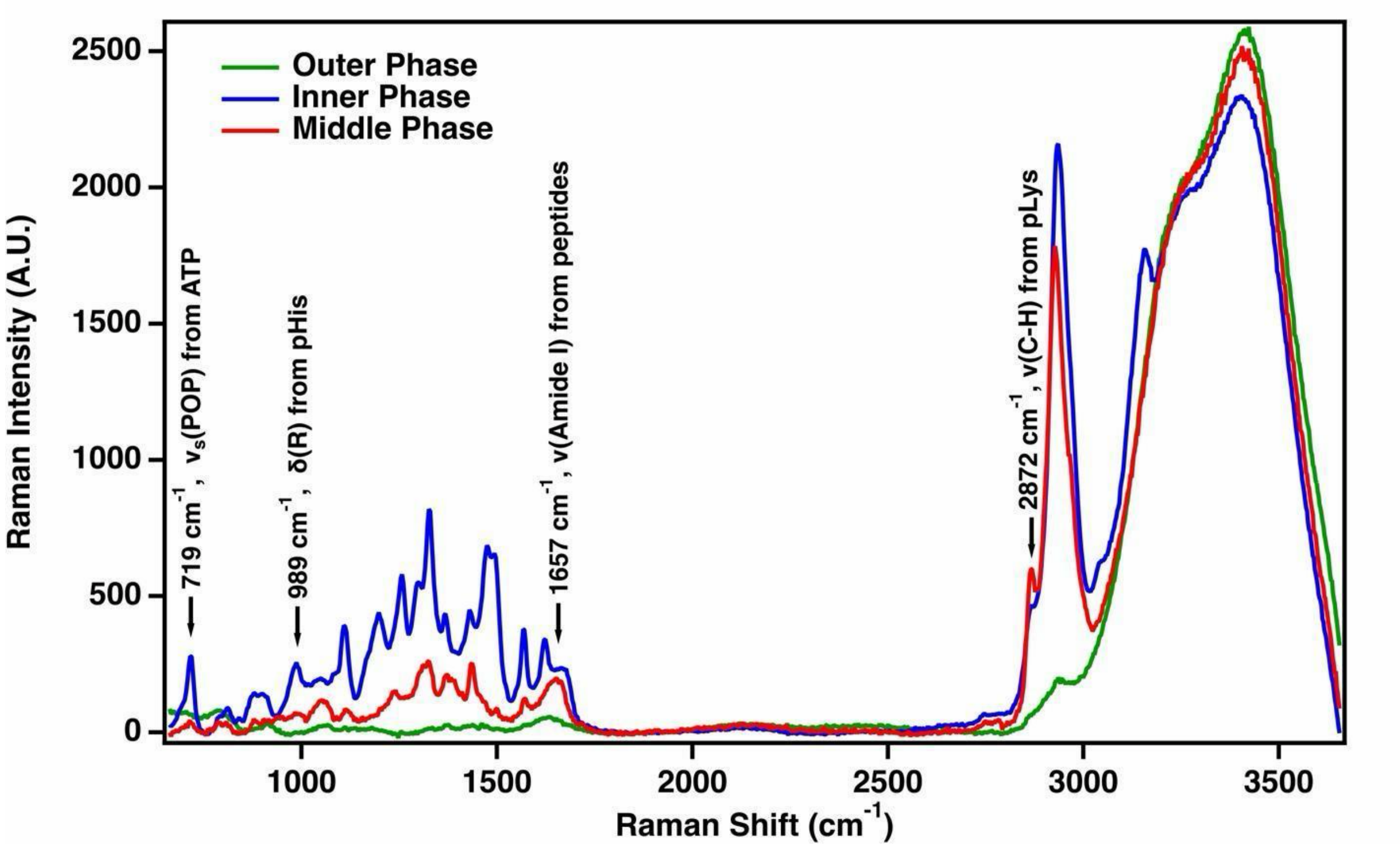

Supplementary Figure 13. Raman spectrum of pHis/ATP multiphase coacervate droplets at pH 4.5. Peaks correspond to vibrational modes of the respective components in the condensed phase. For detailed peak assignments and experimental conditions, see *Supplementary Table 1* and the *Materials and Methods* section.

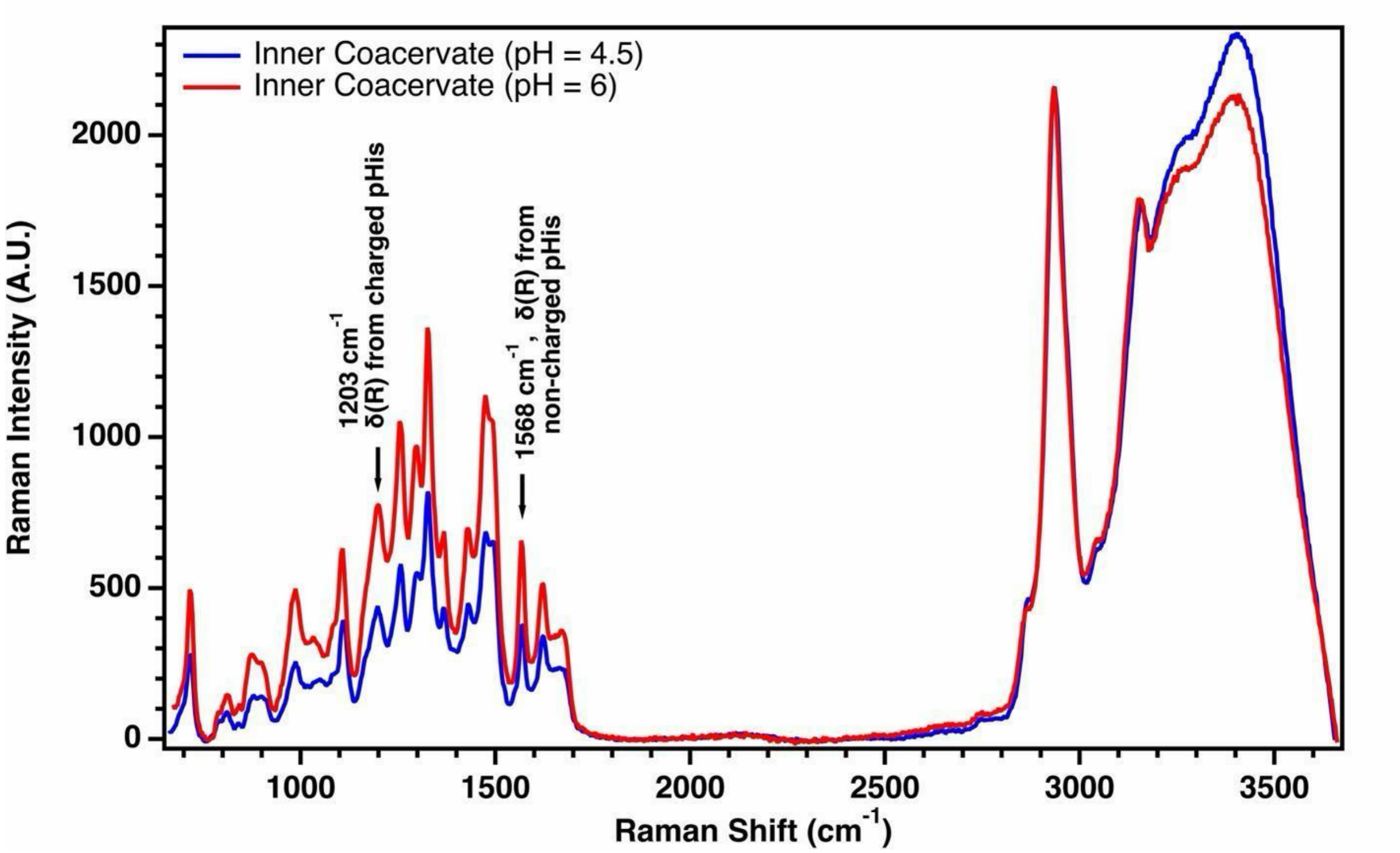

Supplementary Figure 14. Raman spectrum of the inner coacervates of pHis/ATP multiphase coacervate droplets at pH 4.5 (blue) and pH 6 (red). The peaks are normalized to their highest C-H stretching peak. The charged pHis residue (1203 cm^{-1}) and non-charged pHis residue (1568 cm^{-1}) change with the system's pH. Peaks correspond to vibrational modes of the respective components in the condensed phase. For detailed peak assignments and experimental conditions, see *Supplementary Table 1* and the *Materials and Methods* section.

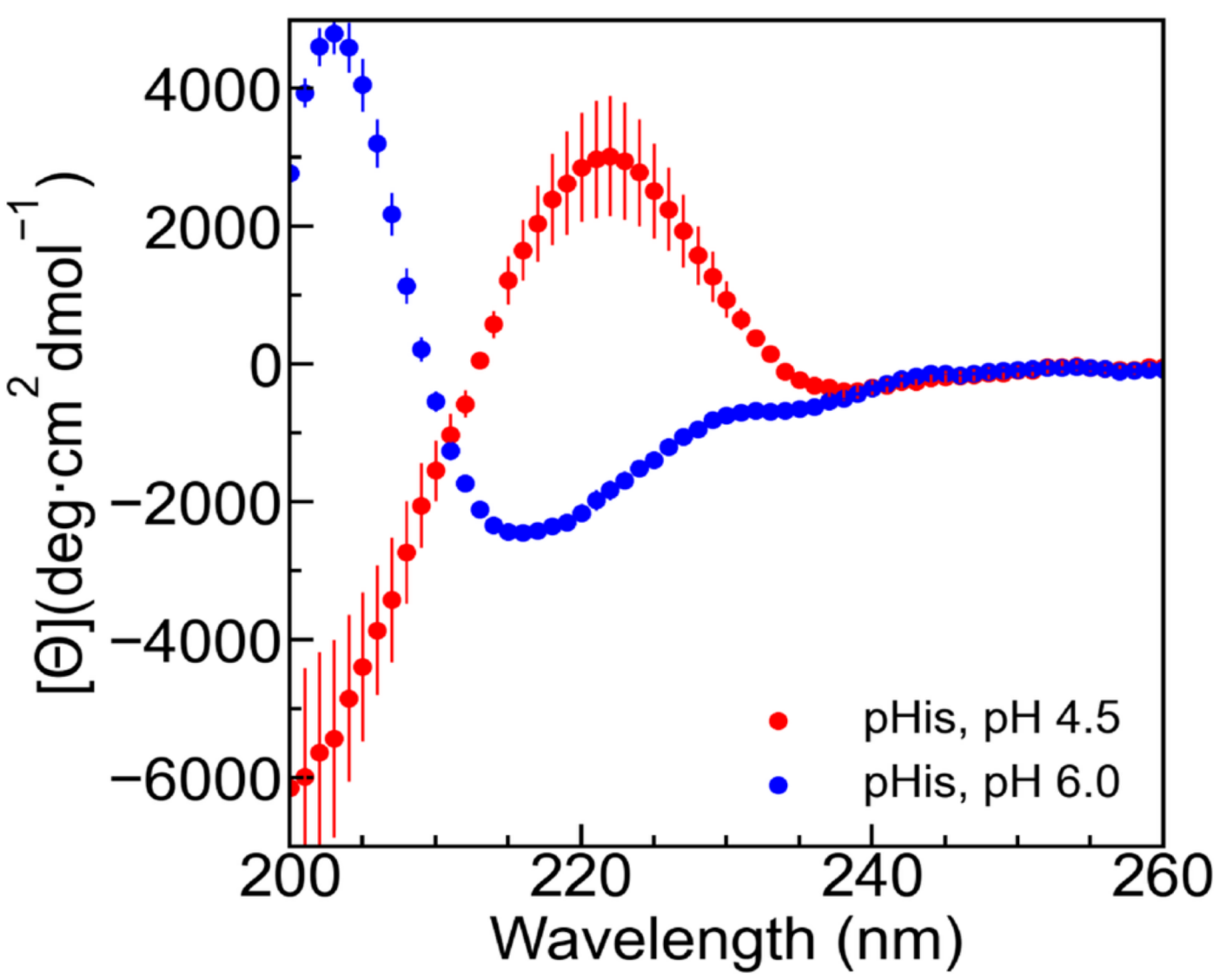

Supplementary Figure 15. Circular dichroism spectra of 2 mM monomer pHis at pH's 4.5 and 6.0, showing coil and β-sheet structures respectively. CD signal normalized to mean residue ellipticity ([Θ]) as described in methods. Stock solutions were pH adjusted using dilute solutions of HCl and NaOH to achieve the desired pH in the absence of added buffer or added salts.

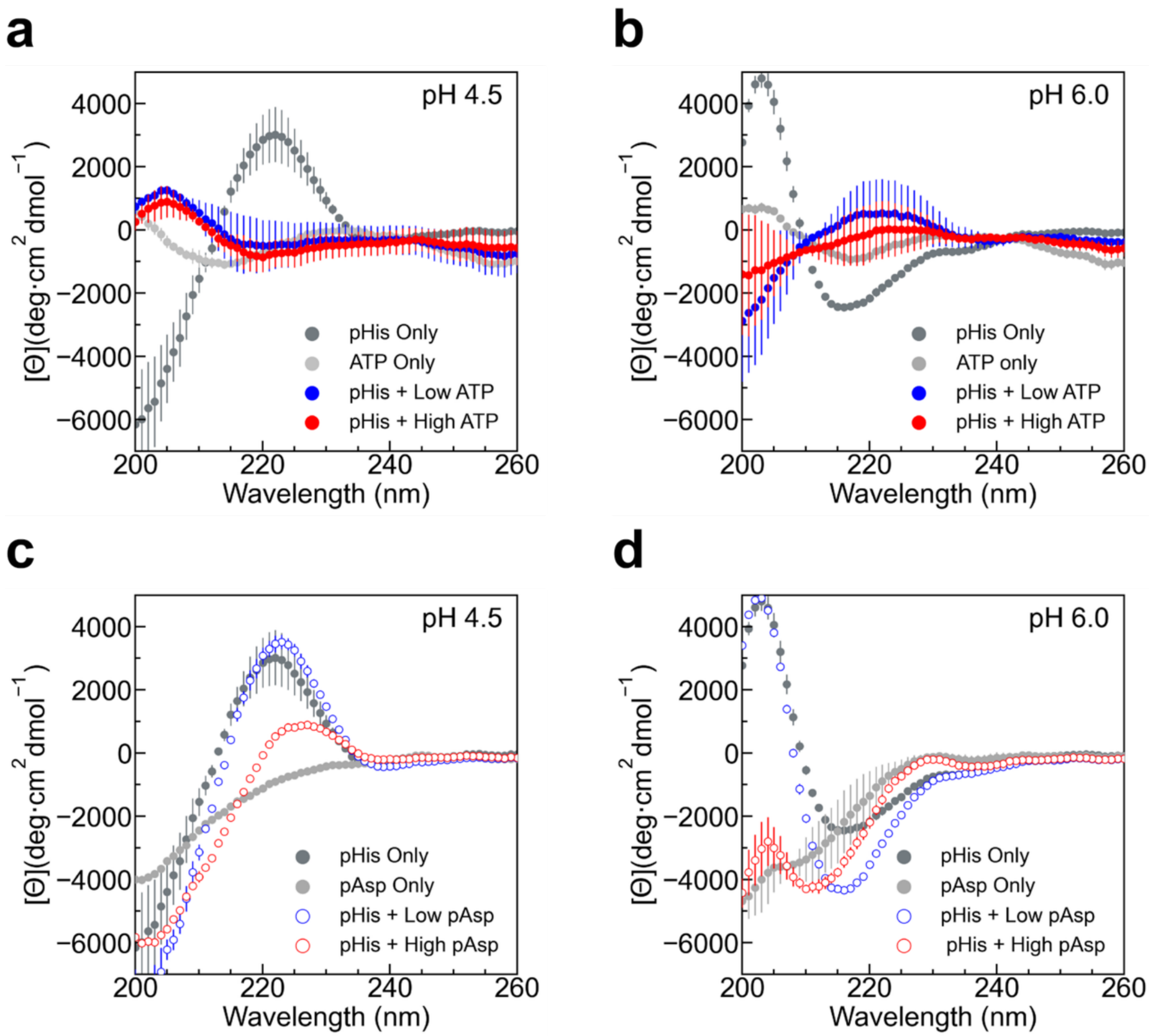

Supplementary Figure 16. Circular dichroism spectra of pHis alone and with ATP **(a, b)** or pAsp **(c, d)** at varying pH. CD spectra normalized to mean residue ellipticity ([Θ]) as described in methods. pHis was fixed at 2 mM monomer concentration. Samples containing ATP (a and b) were prepared at low (0.125 mM, blue curve) or high (0.5 mM ATP, red curve) [ATP]. Samples containing pAsp **(c, d)** were prepared at low (0.5 mM monomer, blue curve) or high (2 mM monomer, red curve) [pAsp]. Control spectra of ATP and pAsp alone at either pH were collected at their respective high concentrations and plotted corresponding to those conditions for comparison (light grey respectively).

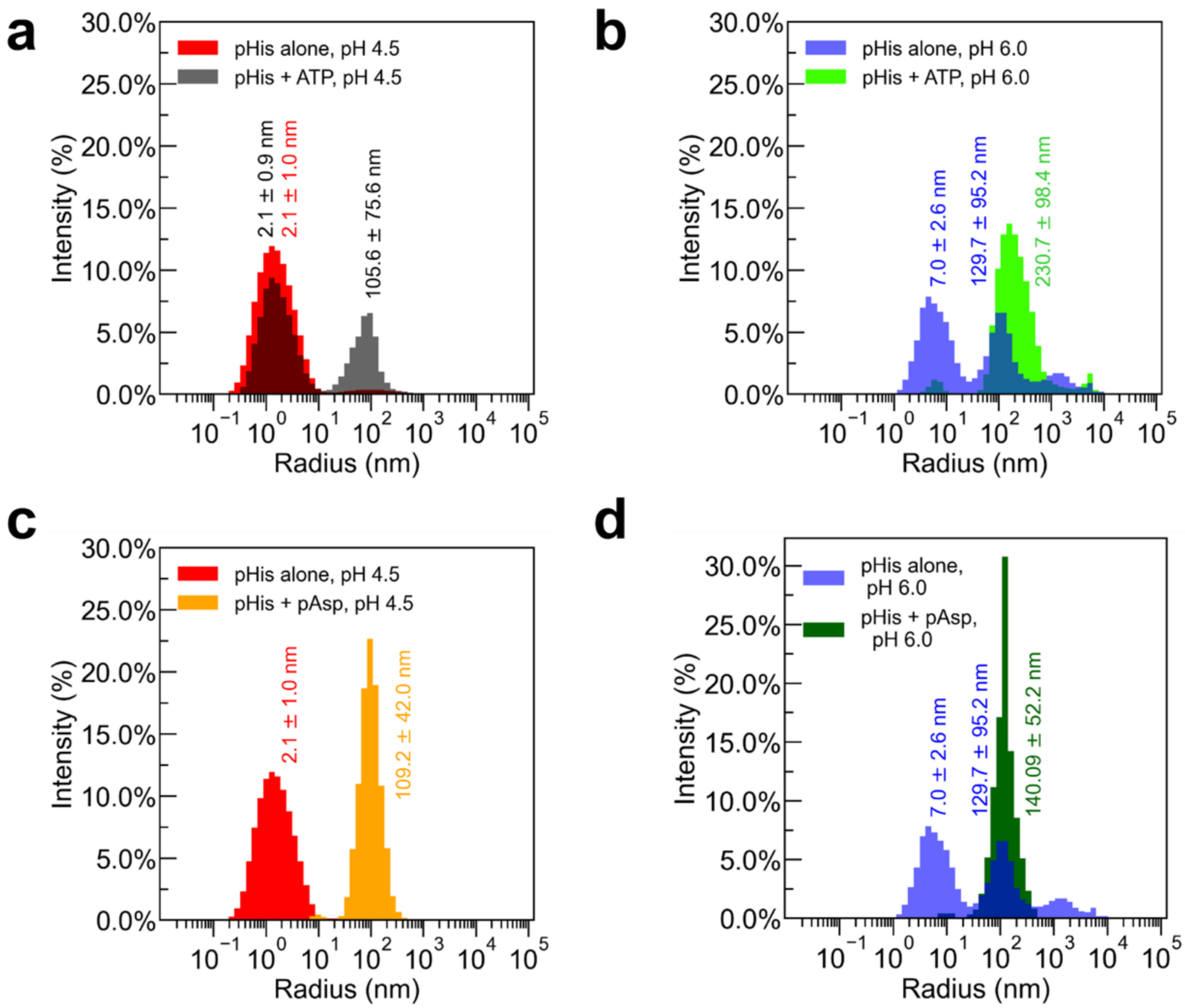

Supplementary Figure 17. Dynamic light scattering of pHis complexed with ATP **(a, b)** or pAsp **(c, d)** at either pH 4.5 or pH 6.0. pHis was fixed at 10 mM monomer for all trials. ATP was fixed at 33 μM final concentration and pAsp was fixed at 100 μM monomer as applicable. Attempts to collect ATP and pAsp only measurements at these concentrations yielded poor data quality, presumably because of the low concentration. Intensity values were used in analysis to mitigate assumptions necessary for mass % conversion.

System	PDI (Cumulant)	PDI ($0<Z_{avg}<100nm$)	PDI ($100<Z_{avg}<300$)
pHis alone pH 4.5	0.33	0.22	-
pHis alone pH 6.0	0.57	0.14	0.54
pHis – pAsp pH 4.5	0.26	-	0.15
pHis – pAsp pH 6.0	0.34	-	0.14
pHis – ATP pH 4.5	0.57	0.17	0.51
pHis – ATP pH 6.0	0.57	-	0.18

Supplementary Table 2.

Table showing the PDI of the various pHis assemblies alone, with pAsp, or ATP at pH 4.5 and 6.0.

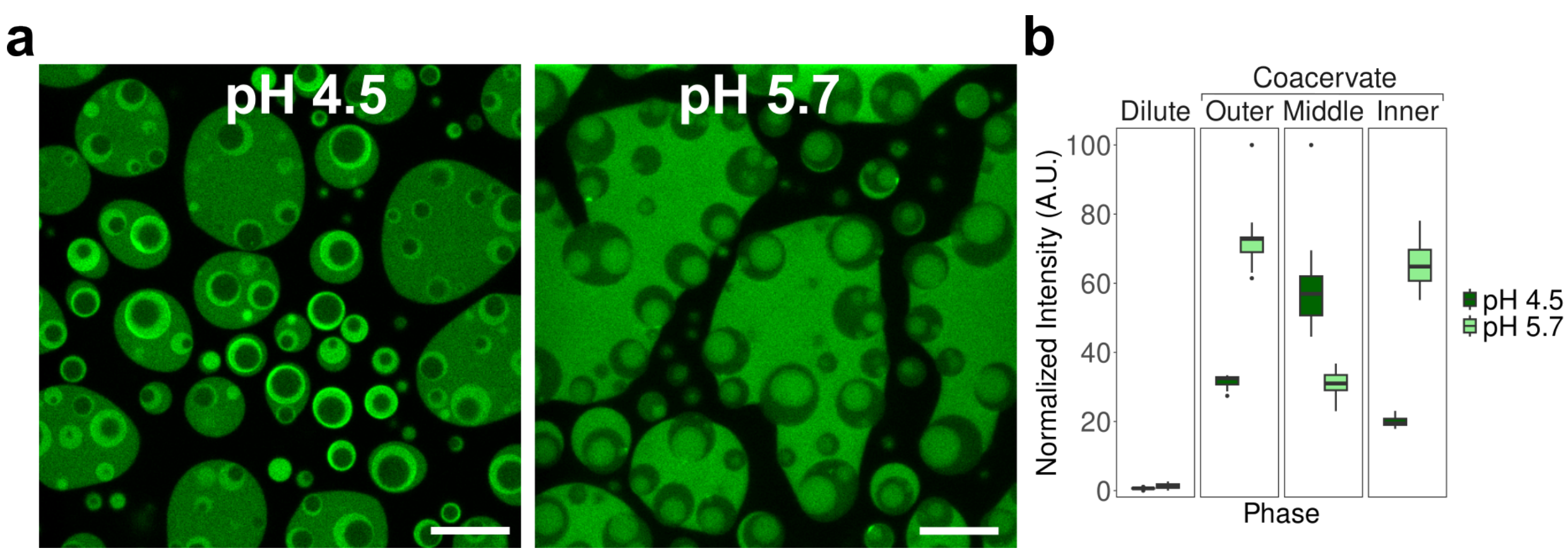

Supplementary Figure 18. Distribution of labeled pAsp in the multiphase coacervate system. **(a)** Confocal microscopy images of DEAE-dextran/CM-dextran, pLys/pAsp, and pHis/pAsp coacervates at pH 4.5 and 5.7. pAsp was labeled with Alexa Fluor 488 (green). **(b)** Box plots show the normalized fluorescence intensity (min–max scaling) of pAsp in each phase at both pH values; each dataset was normalized independently. A minimum of 20 intensity measurements were collected per phase. All scale bars: 10 µm.

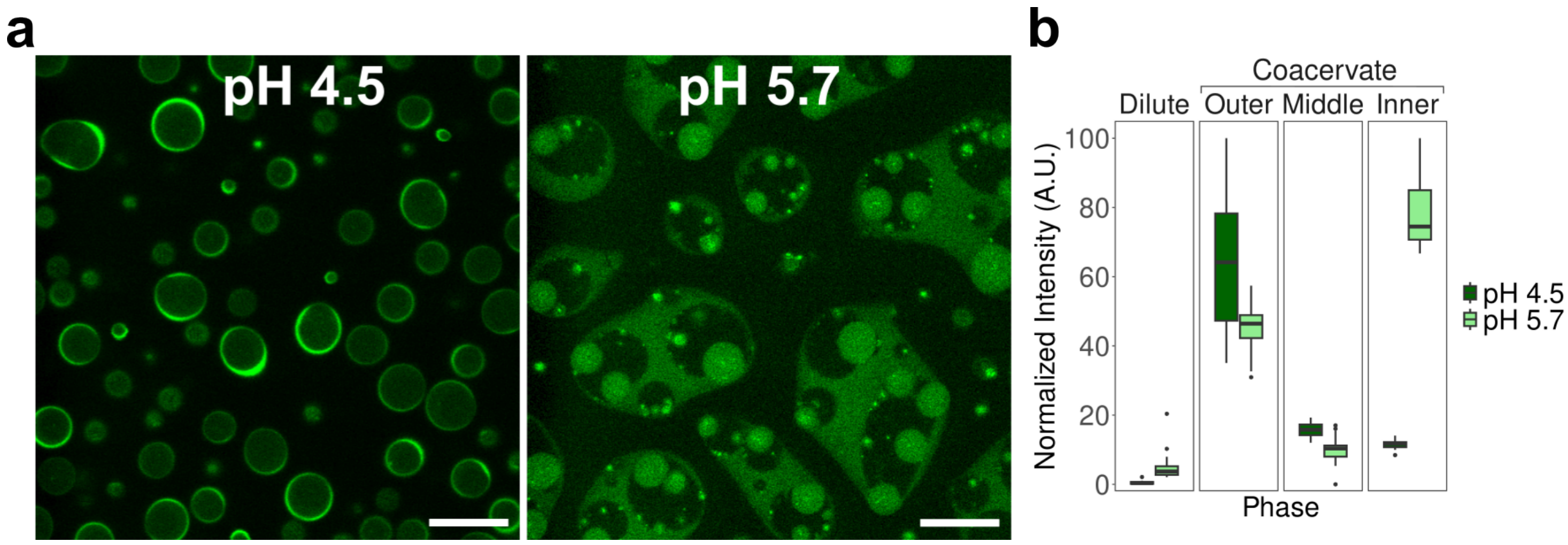

Supplementary Figure 19. Distribution of labeled CM-dextran in the multiphase coacervate system. **(a)** Confocal microscopy images of DEAE-dextran/CM-dextran, pLys/pAsp, and pHis/pAsp coacervates at pH 4.5 and 5.7. CM-dextran was labeled with Alexa Fluor 488 (green). **(b)** Box plots show the normalized fluorescence intensity (min–max scaling) of CM-dextran in each phase at both pH values; each dataset was normalized independently. A minimum of 20 intensity measurements were collected per phase. All scale bars: 10 µm.

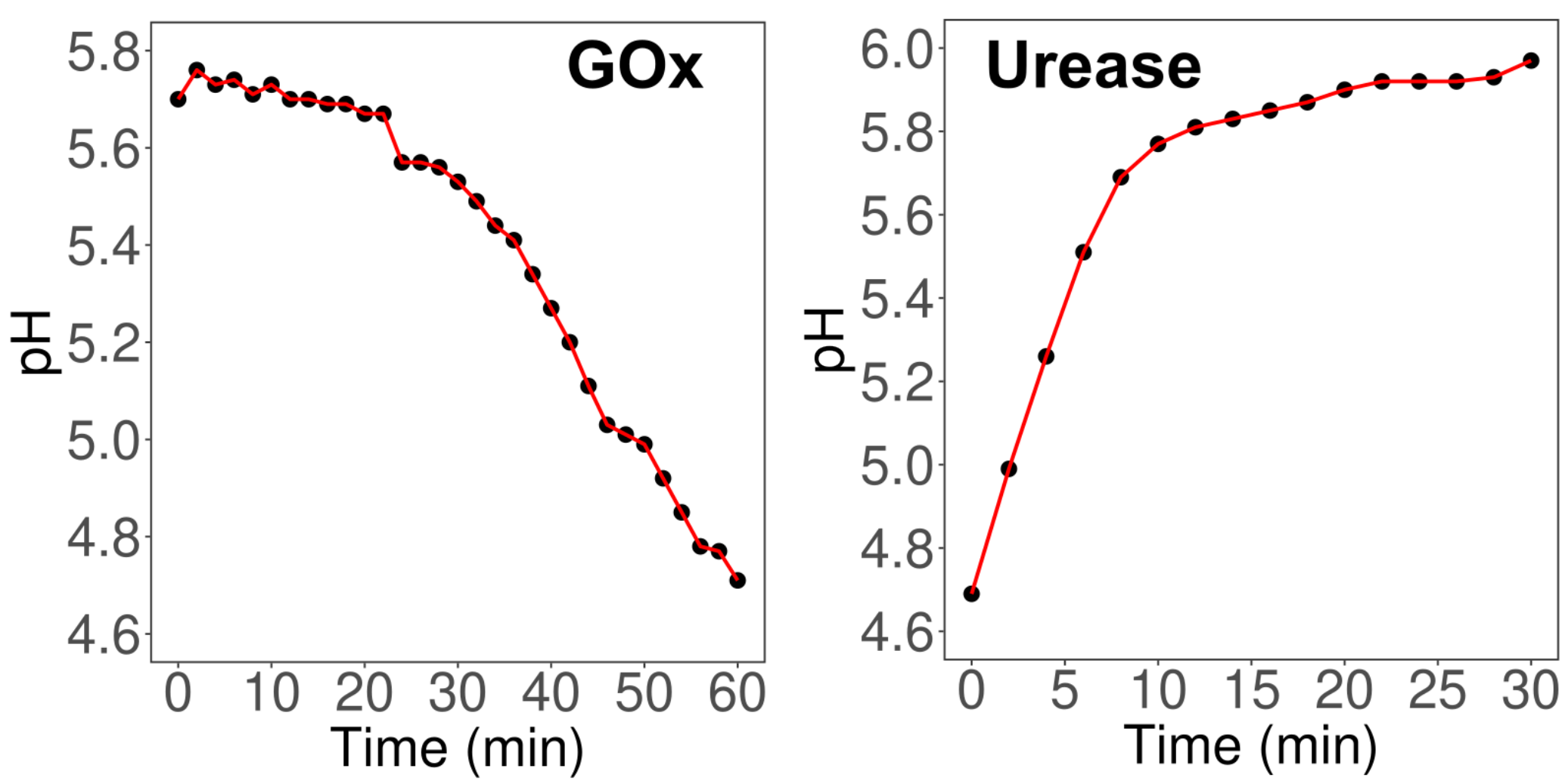

Supplementary Figure 20. Enzyme-mediated pH changes over time for the DEAE-dextran/CM-dextran, pLys/pAsp, and pHis/pAsp multiphase coacervate system containing either 0.2 mg/mL glucose oxidase (GOx) or 0.2 mg/mL urease. The GOx reaction was initiated by the addition of 15 mM glucose, and the urease reaction was initiated by the addition of 5 mM urea. Buffer concentrations were 10 mM MES for the GOx experiment and 10 mM acetate for the urease experiment. Both experiments were performed as bulk measurements, with the pH probe inserted directly into the coacervate suspension. Figure labels indicate the corresponding enzyme used in each condition.

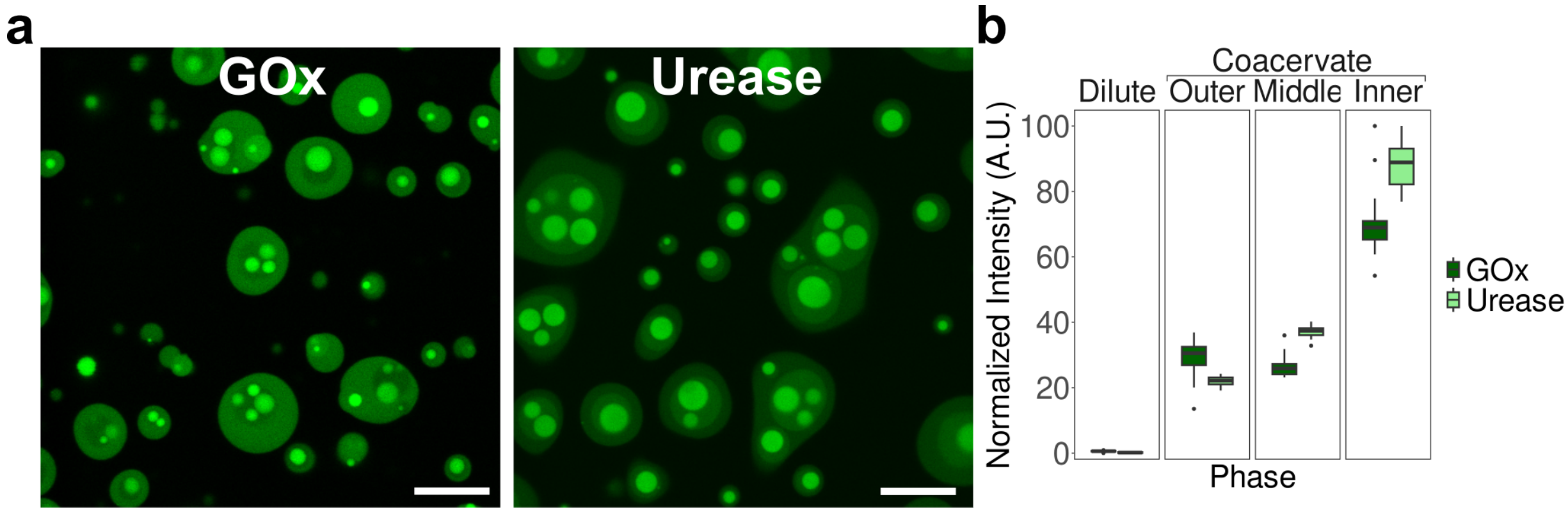

Supplementary Figure 21. Distribution of labeled enzymes in a multiphase coacervate system. **(a)** Confocal microscopy images of DEAE-dextran/CM-dextran, pLys/pAsp, and pHis/pAsp coacervates at pH 4.5 containing either urease or glucose oxidase (GOx), each labeled with Alexa Fluor 488 (green). **(b)** Box plots show the normalized fluorescence intensity (min–max scaling) of urease or GOx within each phase; each enzyme system was normalized independently. A minimum of 20 intensity measurements were collected per phase. All scale bars: 10 µm.

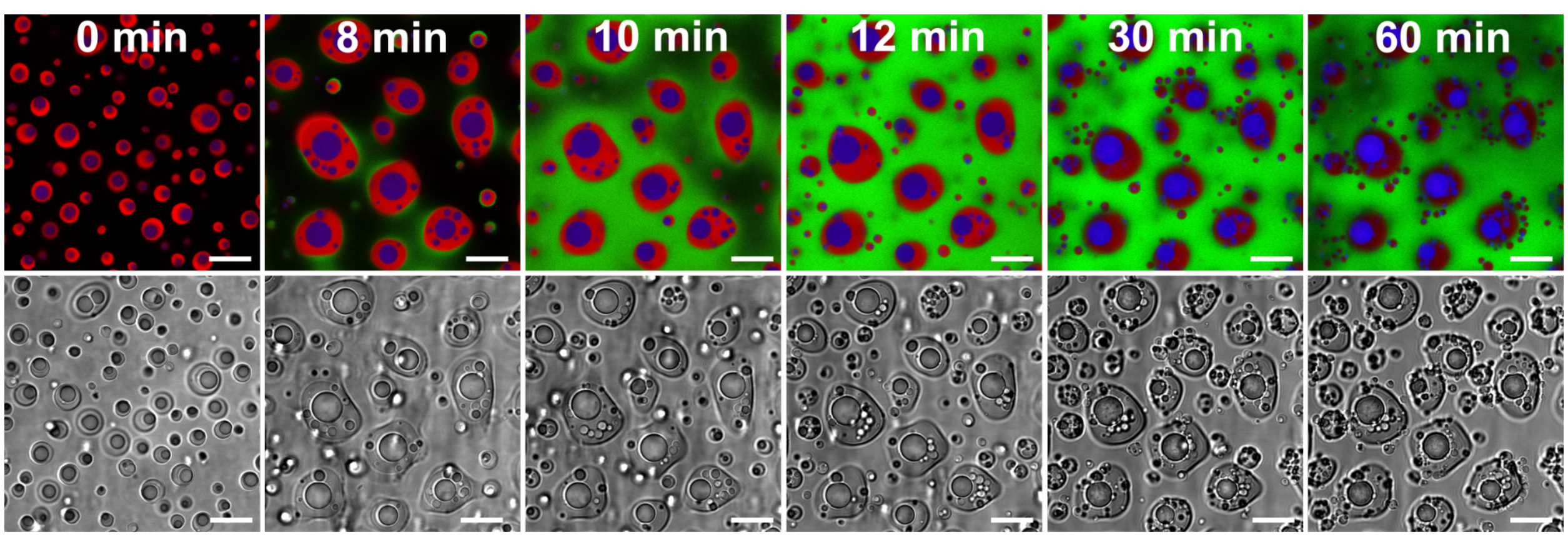

Supplementary Figure 22. Phase reorganization driven by urease-mediated pH increase. Confocal microscopy images of DEAE-dextran/CM-dextran, pLys/pAsp, and pHis/pAsp coacervates containing 0.2 mg.mL^{-1} urease, imaged at pH 4.7 before urea addition (0 min, left). The reaction was initiated by the addition of 5 mM urea, resulting in a pH increase to approximately 6.2 after 60 minutes. DEAE-dextran was labeled with fluorescein (green), pLys with rhodamine (red), and pHis with Alexa Fluor 633 (blue). Separate fluorescence channels were false-colored and merged for clarity. Scale bars: 10 µm.

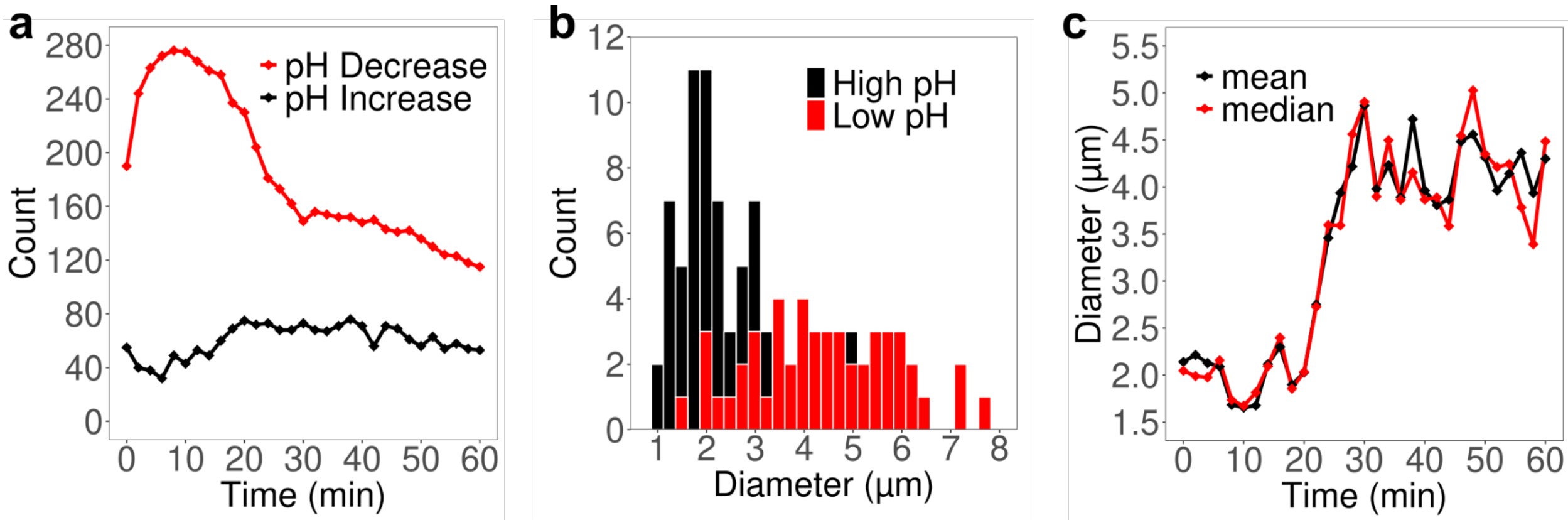

Supplementary Figure 23. Plots showing changes in the physical characteristics of the multiphase coacervate system as a function of pH. **(a)** Number of pHis coacervate droplets observed as the pH decreases (GOx and glucose) or increases (urease and urea). **(b)** Histograms of pHis droplet diameters at high (pH 6.2) and low (pH 4.7) conditions. **(c)** Mean and median diameters of pHis droplets as the pH is decreased using GOx and glucose.

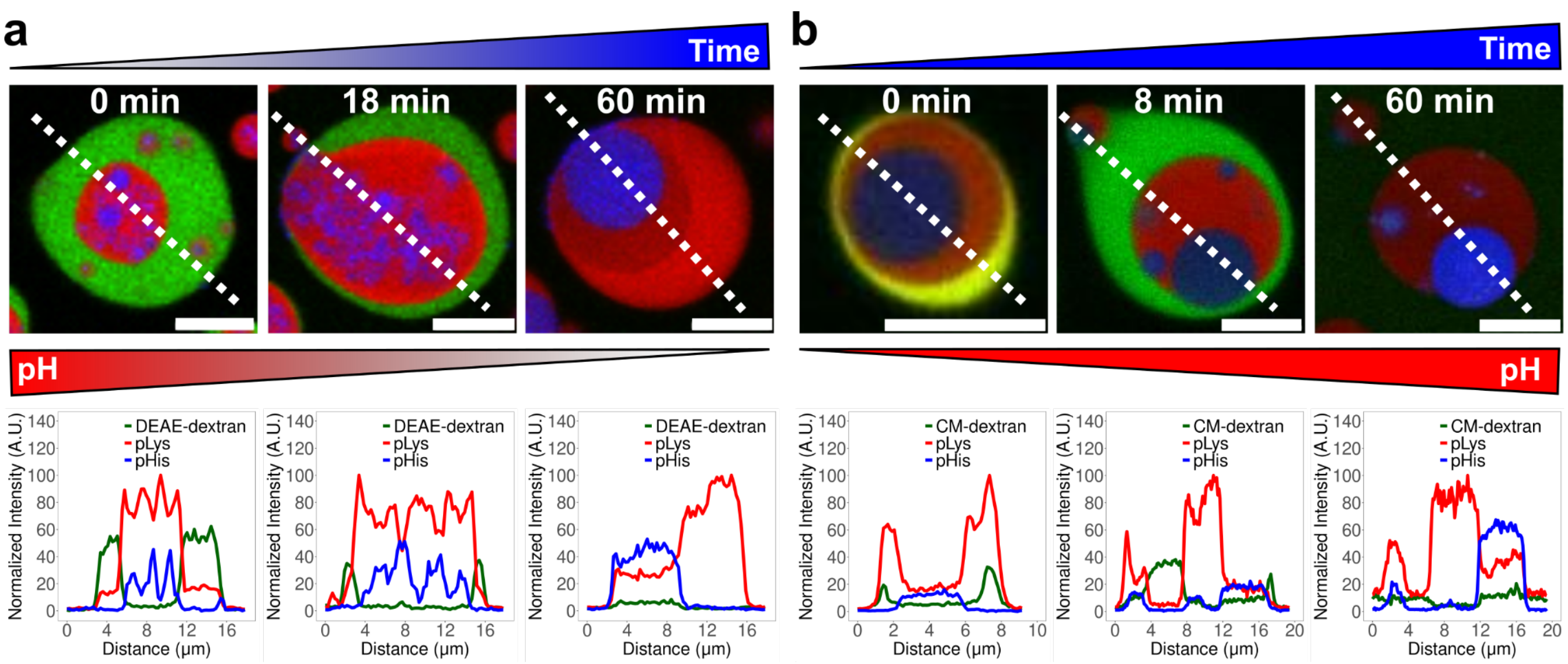

Supplementary Figure 24. (a, b) Confocal microscopy images and corresponding line profiles of DEAE-dextran/CM-dextran, pLys/pAsp, and pHis/pAsp coacervates containing 0.2 mg.mL^{-1} of either GOx **(a)** or urease **(b)**. Reactions were initiated by the addition of 15 mM glucose for GOx **(a)** or 5 mM urea for urease (b). In **(a)**, DEAE-dextran was labeled with fluorescein (green), pLys with rhodamine (red), and pHis with Alexa Fluor 633 (blue). In **(b)**, CM-dextran was labeled with Alexa Fluor 488, pLys with rhodamine, and pHis with Alexa Fluor 633. Scale bars: 10 µm.

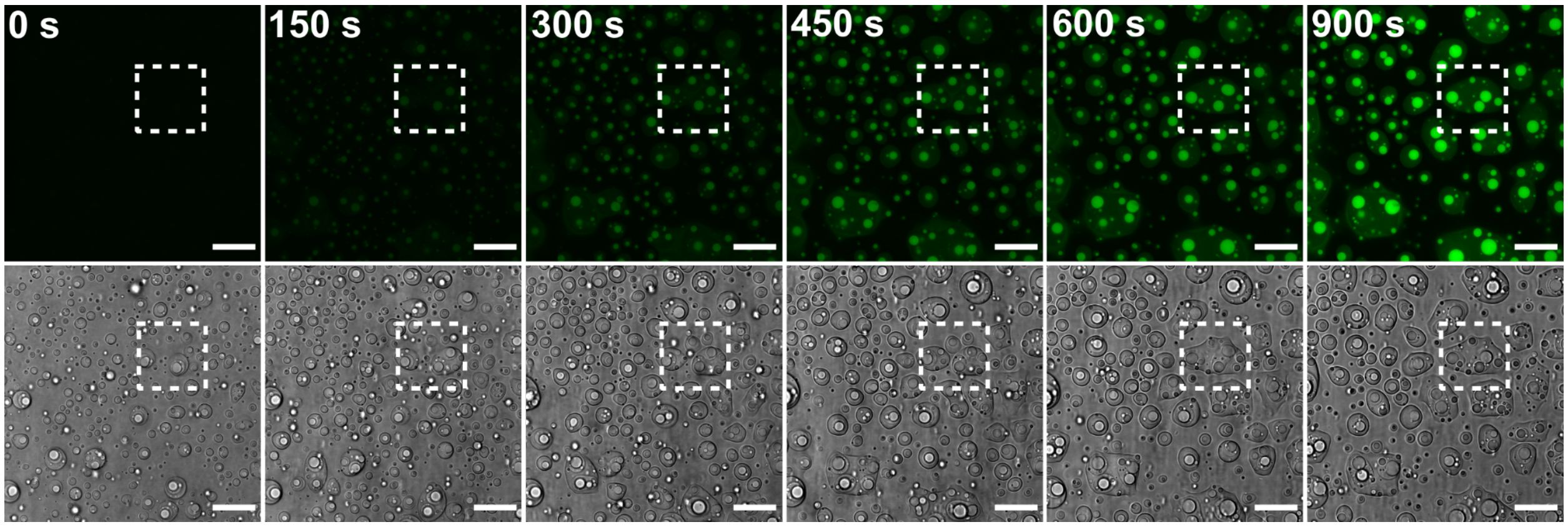

Supplementary Figure 25. Confocal microscopy images of DEAE-dextran/CM-dextran, pLys/pAsp and pHis/pAsp coacervates showing 5(6)-Carboxy-2′,7′-dichlorofluorescein diacetate (CDFDA) hydrolysis over time at pH 5.7 to 5(6)-Carboxy-2′,7′-dichlorofluorescein (green). Dashed line box corresponds to region depicted in Fig. 5d in main text. Scale bars = 20 µm.

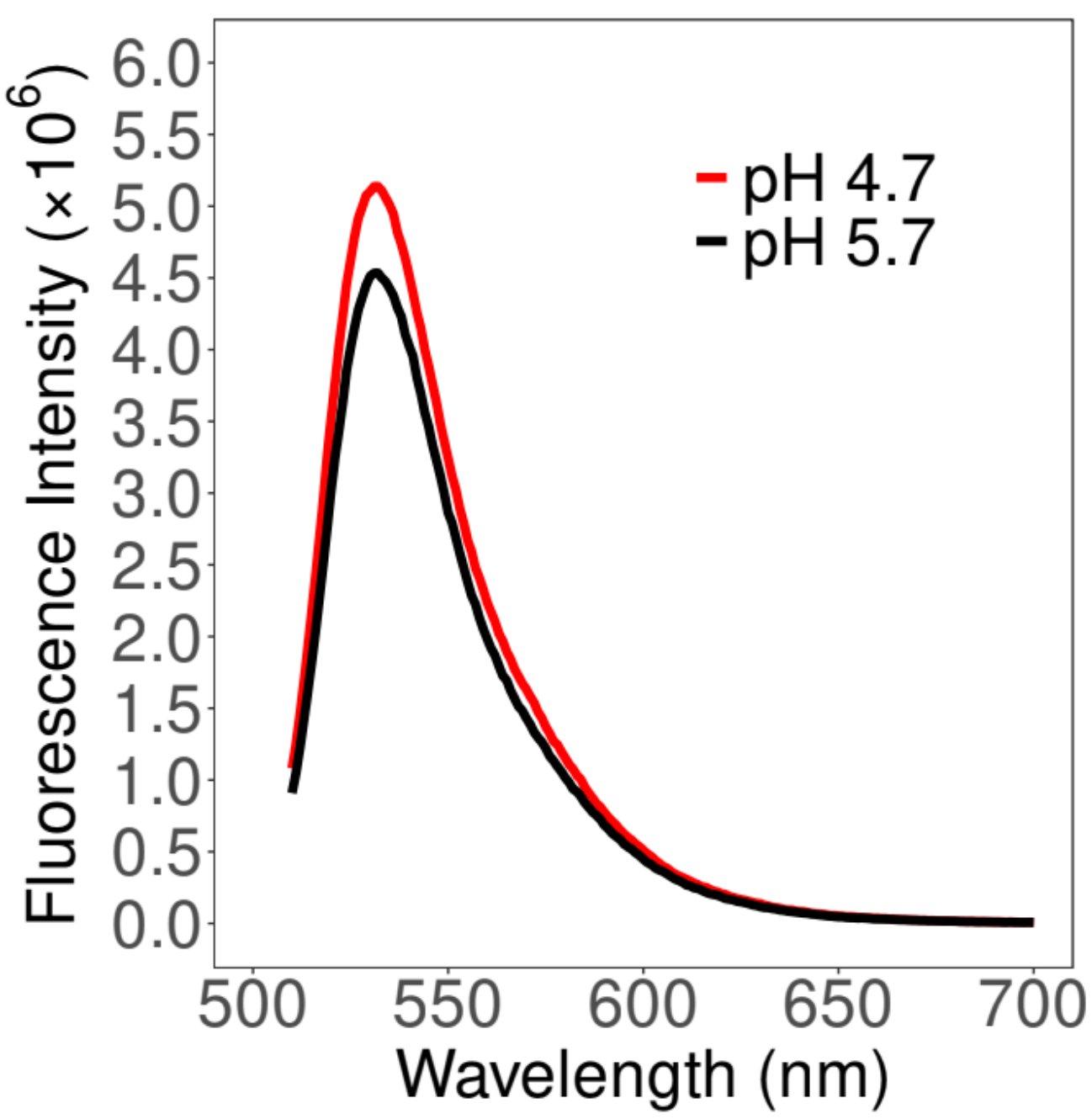

Supplementary Figure 26. Fluorescence spectra of 0.4 µM 5(6)-carboxy-2′,7′-dichlorofluorescein (CDF) in DEAE-dextran/CM-dextran, pLys/pAsp, and pHis/pAsp multiphase coacervates at pH 4.7 and 5.7. The fluorescence intensity at 529 nm was 1.14-fold higher at pH 5.7 than at pH 4.7, and this ratio was used as a correction factor in all subsequent fluorescence spectroscopy kinetics experiments (Fig. 5f–h, Fig. 6d, and Supplementary Figs. 27, 28 and Supplementary Table 3). This spectrum was also used to convert fluorescence units to concentration values in kinetic fluorescence spectroscopy.

Coacervate	pH	Rate (nMs^{-1})	Estimated % Complete
pAsp Multiphase	pH 4.7	0.46	3.5
pAsp Multiphase	pH 5.7	3.4	21
ATP Multiphase	pH 4.7	0.020	0.15
ATP Multiphase	pH 5.7	0.040	1.0
pAsp Single-phase	pH 4.7	0.52	6.3
pAsp Single-phase	pH 5.7	0.080	0.92
ATP Single-phase	pH 4.7	0.00	0.15
ATP Single-phase	pH 5.7	0.00	1.6
No pHis	pH 4.7	0.010	0.030
No pHis	pH 5.7	0.010	0.11
No Polymers	pH 4.7	0.010	0.15
No Polymers	pH 5.7	0.050	0.33
pHis Only	pH 4.7	9.2	40
pHis Only	pH 5.7	9.3	31
Adenosine	pH 5.7	2.6	17
AMP	pH 5.7	6.6	22
ADP	pH 5.7	1.9	9.6
Urease	N/A	3.8	21

Supplementary Table 3.

Conditions of the fluorescence spectroscopy kinetics experiments and percentage completion of CDFDA hydrolysis to CDF in the coacervate system. The relationship between fluorescence intensity and CDF concentration (Supplementary Fig. 26) was used to convert fluorescence signals to concentration, enabling calculation of hydrolysis rates over the first 30 s. Percent completion was determined by comparing the final measured fluorescence intensity to the theoretical maximum, assuming complete conversion of CDFDA to CDF.

Table definitions:

- **pAsp multiphase**: DEAE-dextran/CM-dextran, pLys/pAsp, and pHis/pAsp multiphase system.
- **ATP multiphase**: Same as pAsp multiphase, except ATP in pHis/pAsp is replaced with ATP.
- **pAsp single-phase** / **ATP single-phase**: pHis/pAsp or pHis/ATP single-phase systems, respectively.
- **No pHis**: CM-dextran/DEAE-dextran and pLys/pAsp multiphase coacervate without pHis.
- **No polymers**: Buffer only, no coacervates.
- **pHis only**: pHis in solution without partner ions or coacervates.

- **Adenosine**, **AMP**, **ADP**: ATP in a CM-dextran/DEAE-dextran, pLys/pAsp, and pHis/ATP multiphase system replaced with an equal concentration of the indicated compound.
- **Urease**: Urease reaction (5 mM urea) in a DEAE-dextran/CM-dextran, pLys/pAsp, and pHis/pAsp multiphase system.

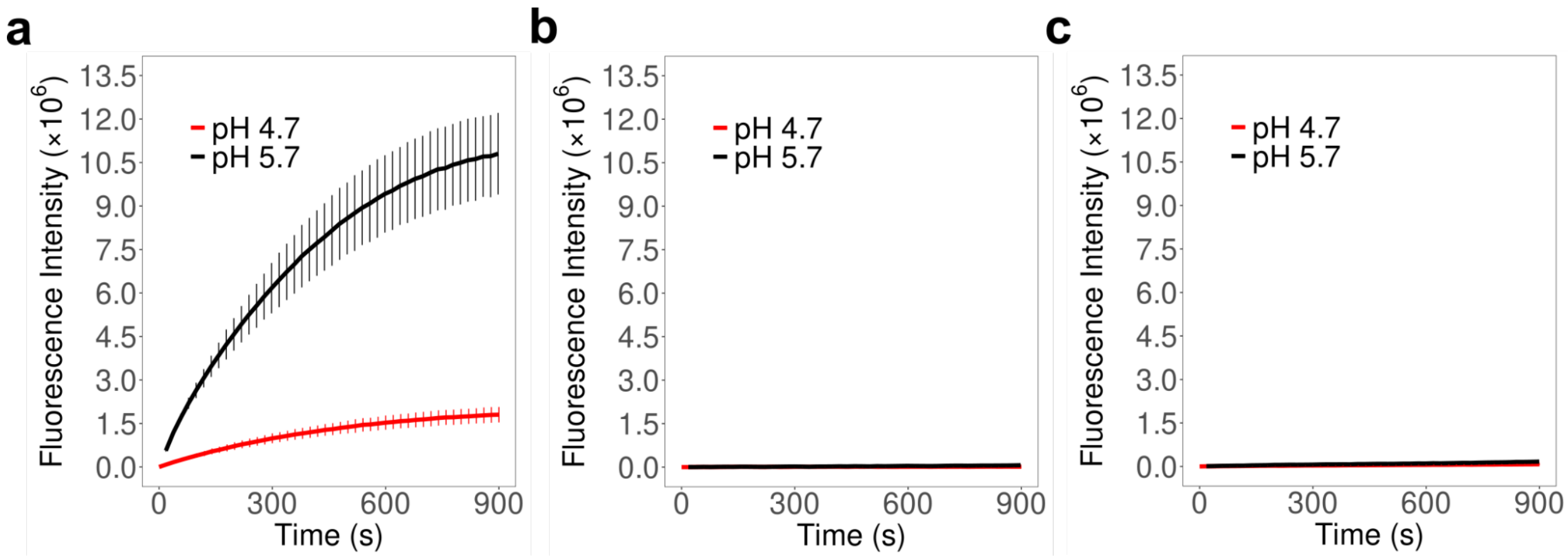

Supplementary Figure 27. (a–c) Fluorescence spectroscopy kinetics showing the hydrolysis of 5 μM CDFDA in: **(a)** DEAE-dextran/CM-dextran, pLys/pAsp, and pHis/pAsp multiphase coacervates (without ATP); **(b)** DEAE-dextran/CM-dextran and pLys/pAsp multiphase coacervates (without pHis); and **(c)** buffer only (no coacervates).

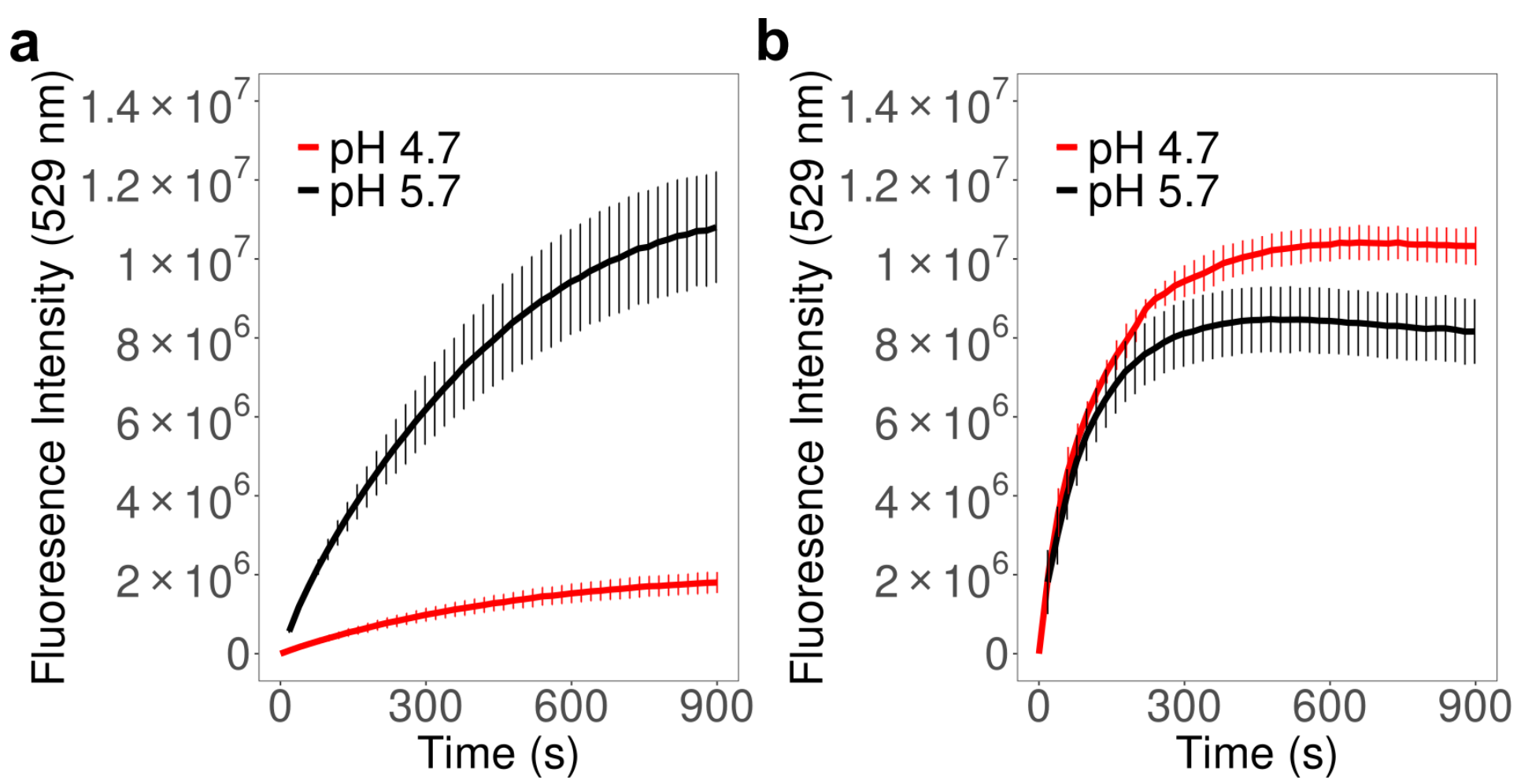

Supplementary Figure 28. (a, b) Fluorescence spectroscopy kinetics showing the hydrolysis of CDFDA at pH 4.7 and 5.7 in: **(a)** DEAE-dextran/CM-dextran, pLys/pAsp, and pHis/pAsp multiphase coacervates (without ATP); and **(b)** pHis (10 μM) alone, without coacervates. To keep fluorescence within the detector's linear range, experiments in **(a)** were performed with 5 μM CDFDA, and those in **(b)** with 2.5 μM CDFDA.

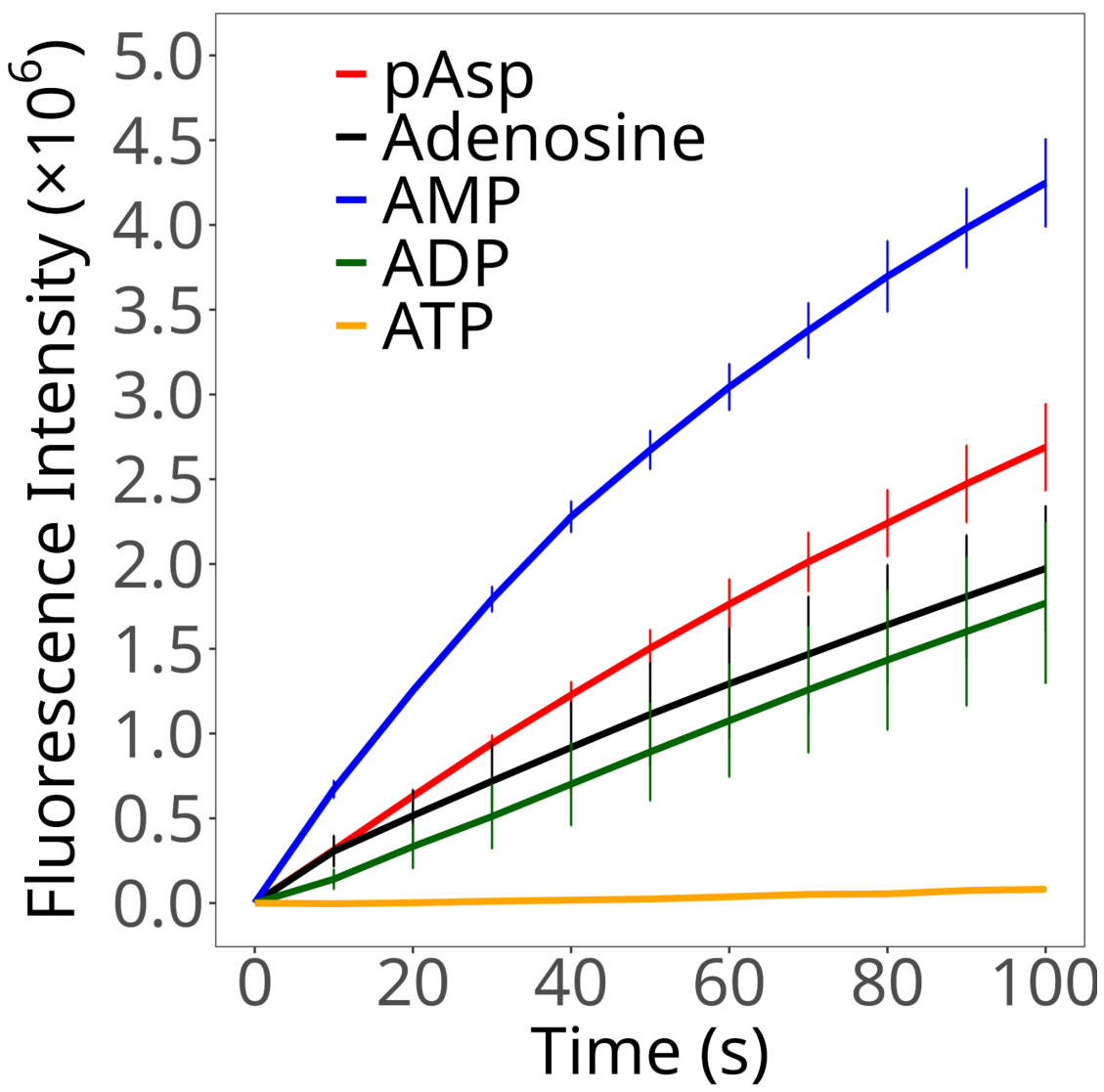

Supplementary Figure 29. Fluorescence spectroscopy kinetics of CDFDA hydrolysis over the initial 100 seconds of the reaction in the multiphase system in the presence of pAsp, adenosine, AMP, ADP, and ATP at pH 5.7. This expanded early timepoint view highlights the differences in initial hydrolysis rates between nucleotide analogues with differing numbers of phosphate groups. Error bars represent standard deviations from a minimum of 3 independent measurements per condition.

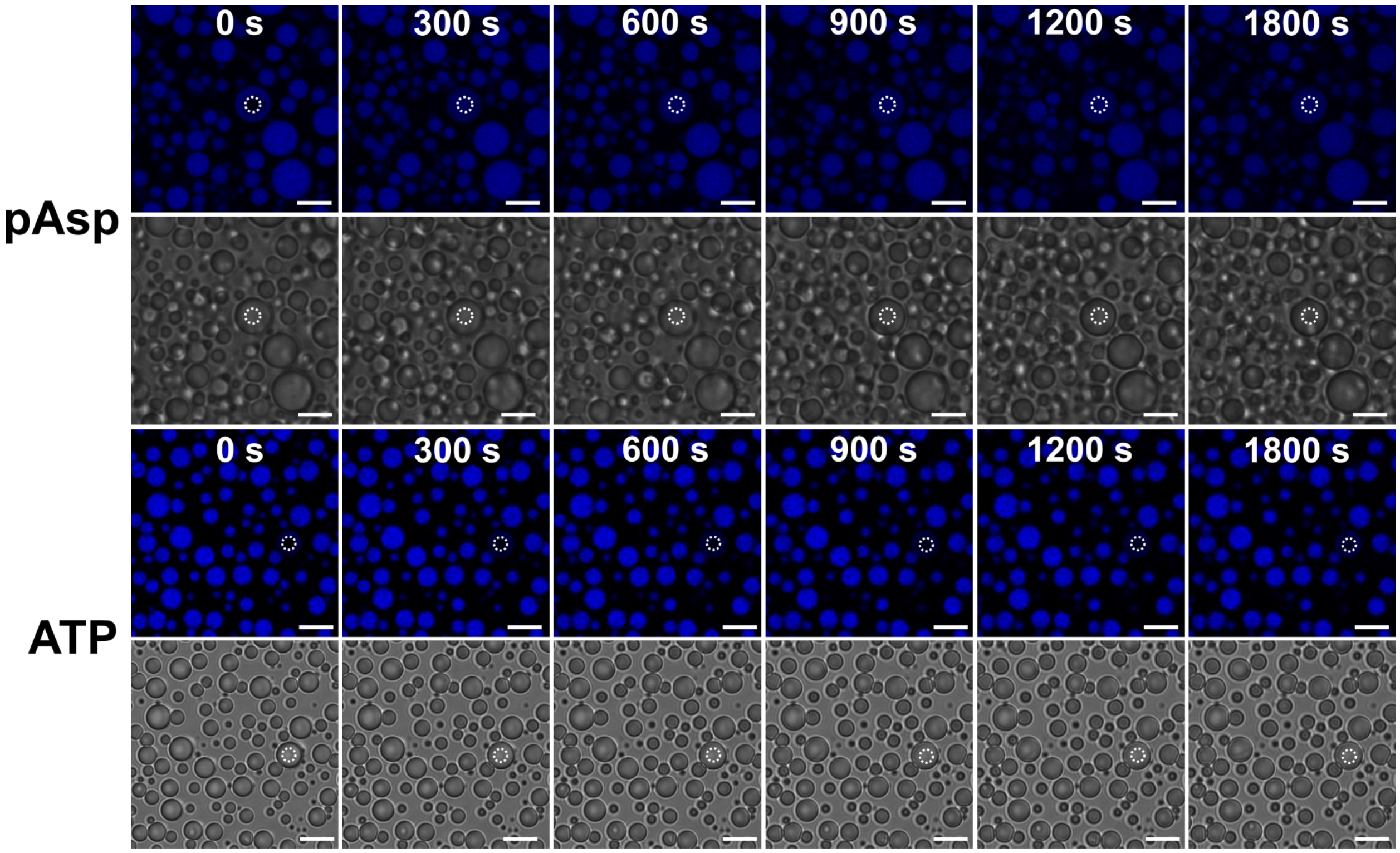

Supplementary Figure 30. Confocal microscopy images showing the fluorescence recovery after photobleaching of pHis/pAsp or pHis/ATP single-phase coacervates at pH 5.7 (pHis is labeled with Alexa Fluor 633, which is blue). All images are false-colored for clarity. All scale bars = 10 μm.

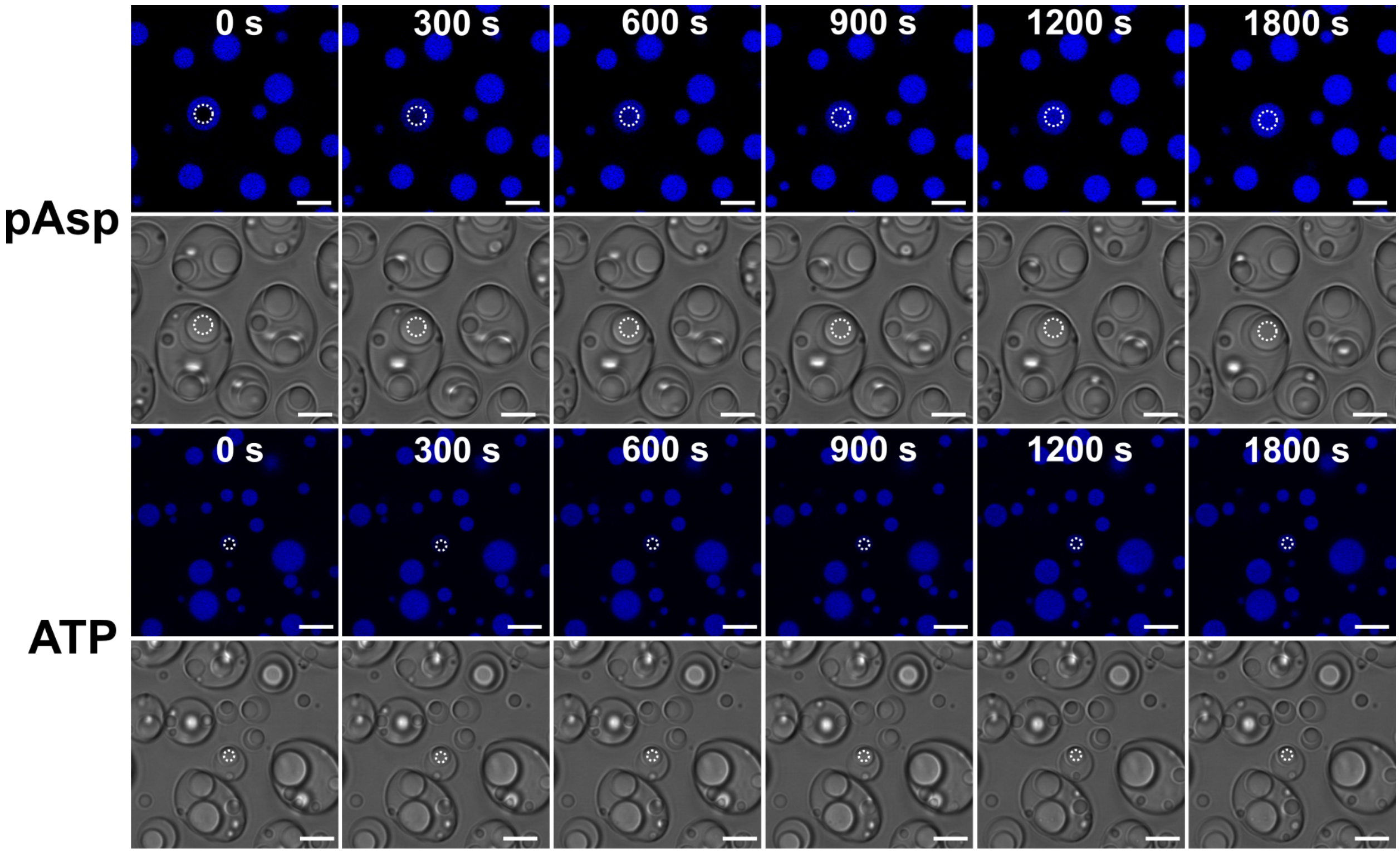

Supplementary Figure 31. Confocal microscopy images showing the fluorescence recovery after photobleaching of pHis rich coacervate phase (labeled with Alexa Fluor 633) contained within a multiphase coacervate system. Top two rows consist of DEAE-dextran/CM-dextran, pLys/pAsp and pHis/pAsp coacervates at pH 5.7. Bottom two rows consist of DEAE-dextran/CM-dextran, pLys/pAsp and pHis/ATP coacervates at pH 5.7. In both systems, pHis was labeled with Alexa Fluor 633 (blue) and images are false-colored for clarity. All scale bars = 10 µm.

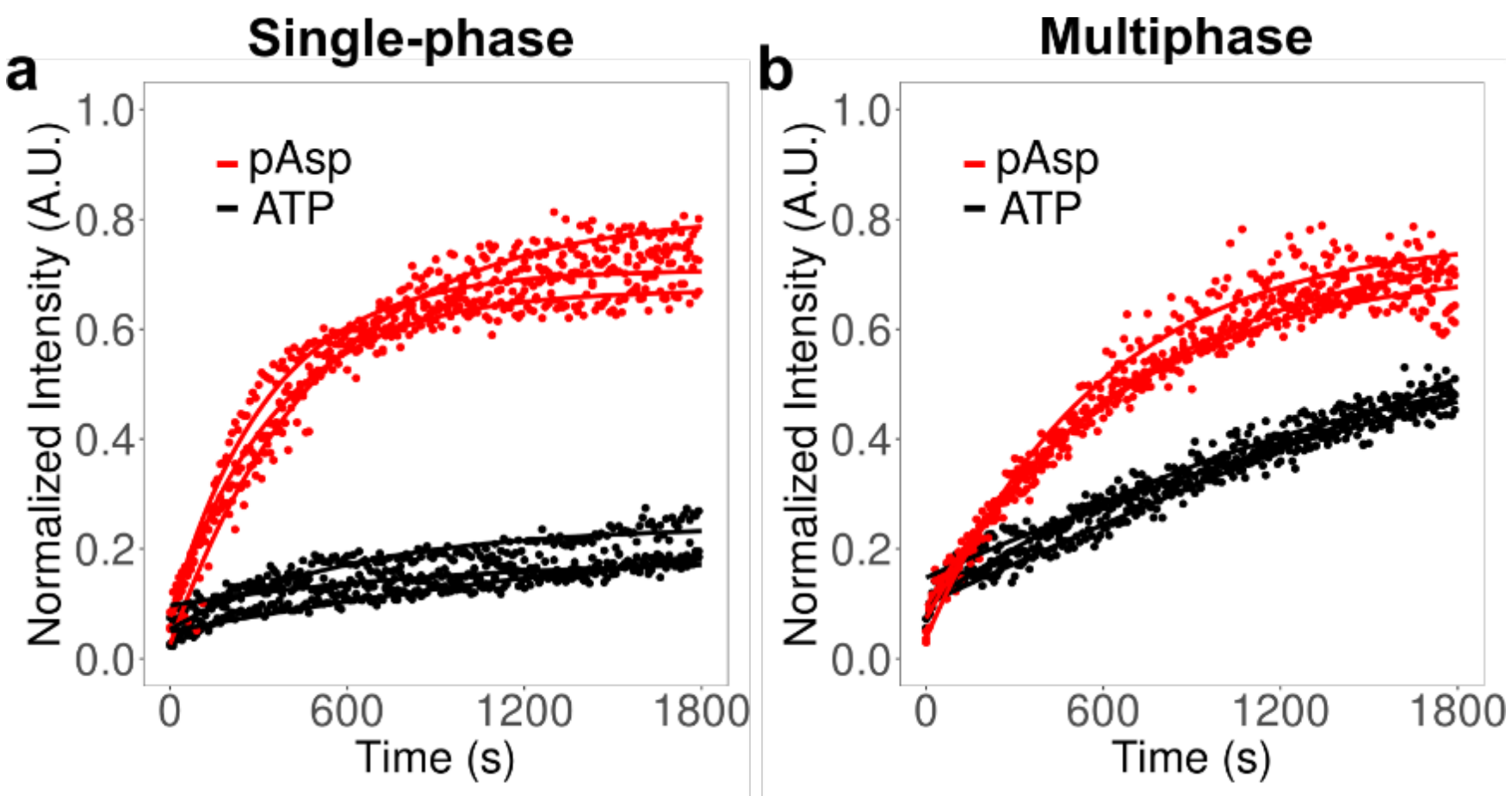

Supplementary Figure 32. (a, b) Fluorescence recovery after photobleaching (FRAP) of the pHis-rich phase (labeled with Alexa Fluor 633) in **(a)** pHis/pAsp and pHis/ATP single-phase systems, and **(b)** DEAE-dextran/CM-dextran, pLys/pAsp, and either pHis/pAsp or pHis/ATP multiphase systems, all at pH 5.7. In both cases, the pAsp-complexed systems showed faster recovery, with the difference being more pronounced in the single-phase system than in the multiphase system. Panel **(a)** corresponds to microscopy images in Supplementary Fig. 26, and panel **(b)** to those in Supplementary Fig. 27.

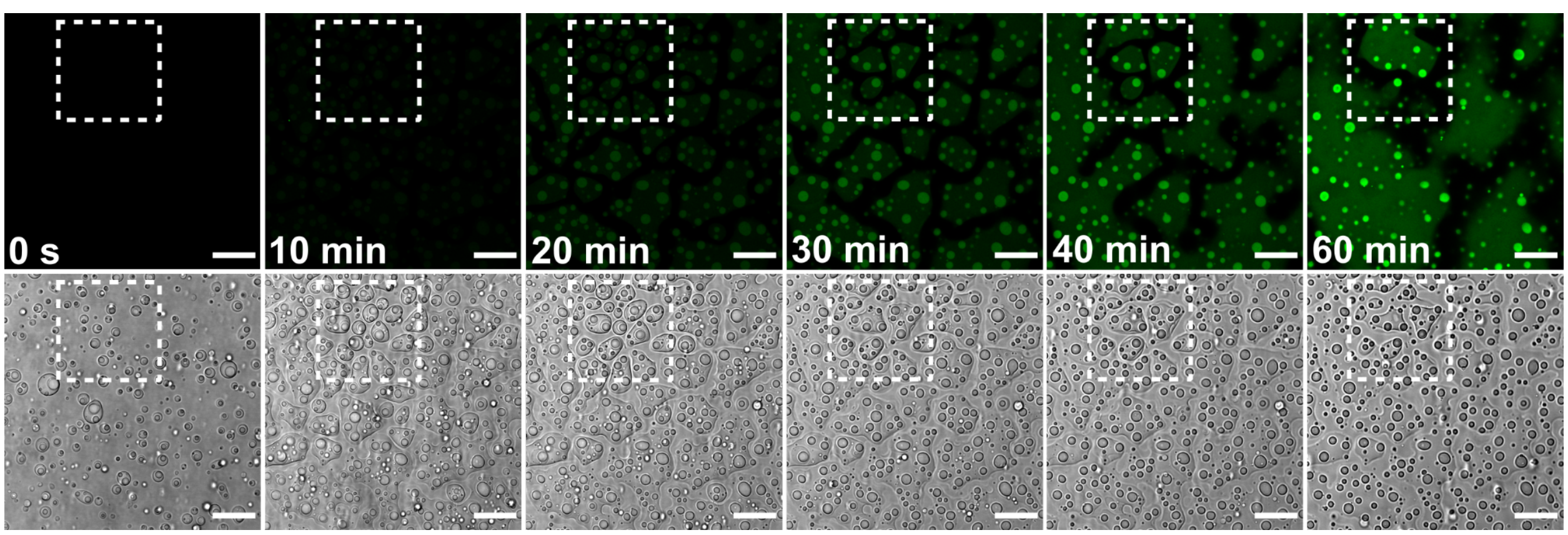

Supplementary Figure 33. Confocal microscopy images showing CDFDA hydrolysis in DEAE-dextran/CM-dextran, pLys/pAsp, and pHis/pAsp coacervates. The system was initially at pH 4.7, which increased to pH 6.5 over the course of the experiment through urease-mediated hydrolysis of urea. The dashed box indicates the region shown in Fig. 6b of the main text. Scale bars: 20 µm.

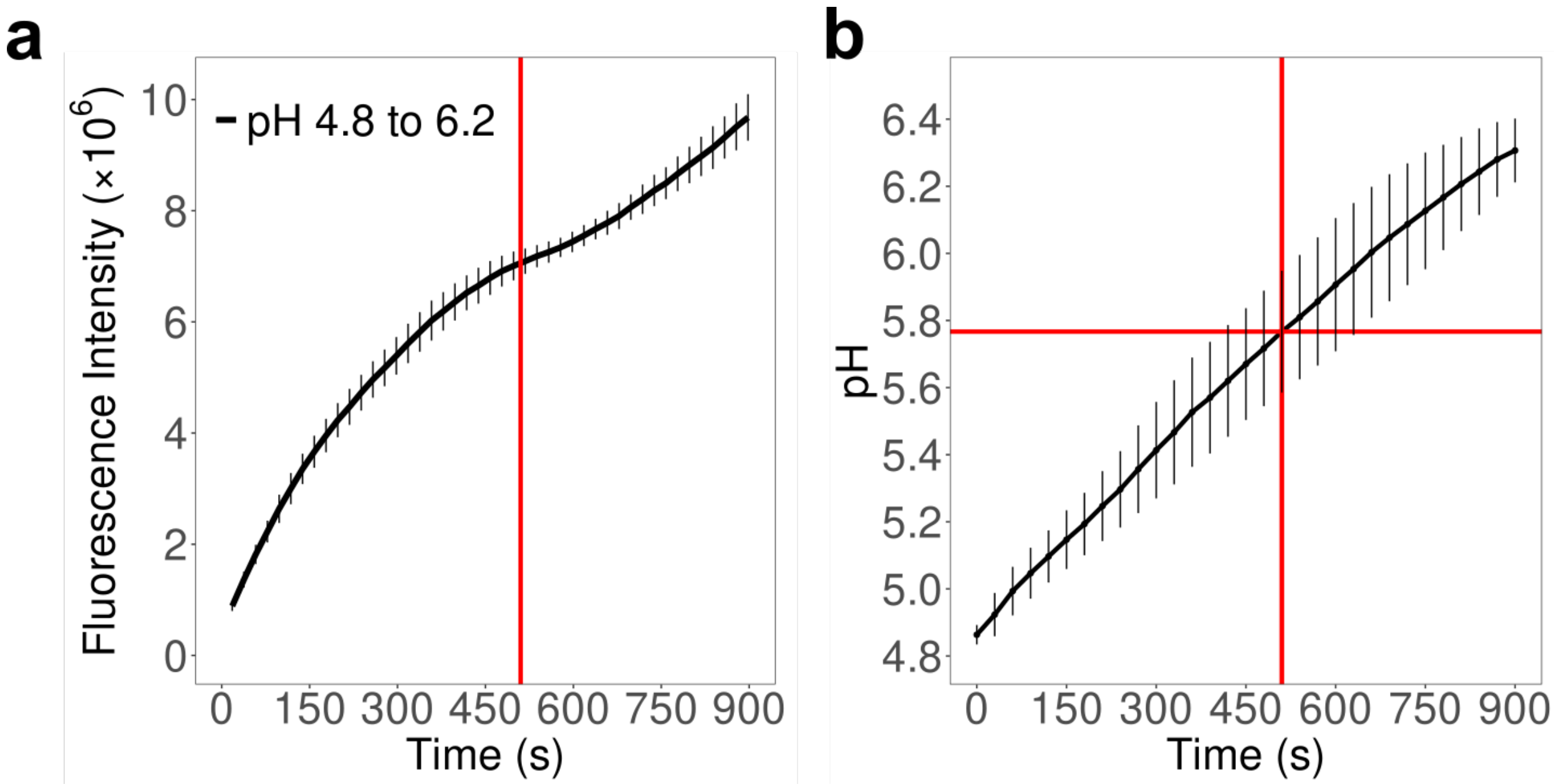

Supplementary Figure 34. (a) Fluorescence spectroscopy kinetics showing the hydrolysis of CDFDA in the DEAE-dextran/CM-dextran, pLys/pAsp, and pHis/pAsp multiphase system, in which the pH increases from 4.8 to 6.2 over 900 seconds due to urease-mediated hydrolysis of 5 mM urea (λ = 529 nm). The corresponding pH change (**b**) was measured by inserting a pH electrode directly into the multiphase coacervate suspension. The red line indicates the time point (510 s) at which the rate of fluorescence intensity change begins to level off in the S-shaped plot (**a**); this corresponds to a measured pH of 5.76 (**b**).

Supplementary References

1. Thermo Fisher Scientific. *Bioconjugation and Crosslinking Technical Handbook.* Thermo Fisher Scientific, Waltham, MA, USA. Available at: https://assets.thermofisher.com/TFS-Assets/BID/Handbooks/bioconjugation-technical-handbook.pdf (accessed 2025).
2. Razunguzwa, T. T.; Warrier, M.; Timperman, A. T. ESI-MS Compatible Permanent Coating of Glass Surfaces Using Poly(ethylene glycol)-Terminated Alkoxysilanes for Capillary Zone Electrophoretic Protein Separations. *Anal. Chem.* **2006**, *78*, 4326–4333.
3. Haidekker, M. A.; Theodorakis, E. A. Environment-Sensitive Behavior of Fluorescent Molecular Rotors. *J. Biol. Eng.* **2010**, *4*, 11.
4. Eysel, H. H.; Lim, K. T. Raman Intensities of Phosphate and Diphosphate Ions in Aqueous Solution. *J. Raman Spectrosc.* **1988**, *19*, 535–539.
5. Rimai, L.; Cole, T.; Parsons, J. L.; Hickmott, J. T.; Carew, E. B. Studies of Raman Spectra of Water Solutions of Adenosine Tri-, Di-, and Monophosphate and Some Related Compounds. *Biophys. J.* **1969**, *9*, 320–329.

6. Majoube, M. Adenine Residue: A Normal-Coordinate Analysis of the Vibrational Spectra. *Biopolymers* **1985**, *24*, 2357–2369.
7. Taylor, N. O.; Wei, M.-T.; Brangwynne, C. P. Quantifying Dynamics in Phase-Separated Condensates Using Fluorescence Recovery after Photobleaching. *Biophys. J.* **2019**, *117*, 1285–1300.
8. Pflüger, F.; Hernández, B.; Ghomi, M. Vibrational Analysis of Amino Acids and Short Peptides in Hydrated Media. VII. Energy Landscapes, Energetic and Geometrical Features of L-Histidine with Protonated and Neutral Side Chains. *J. Phys. Chem. B* **2010**, *114*, 9072–9083.
9. Aylward, N.; Koenig, J. Raman Spectra of Poly(adenylic acid). *Macromolecules* **1970**, *3* (5), 590–596.
10. Mathlouthi, M.; Seuvre, A.-M.; Koenig, J. L. F.T.-I.R. and Laser-Raman Spectra of Adenine and Adenosine. *Carbohydr. Res.* **1986**, *146*, 15–27.
11. Ashikawa, I.; Itoh, K. Raman Spectra of Polypeptides Containing L-Histidine Residues and Tautomerism of Imidazole Side Chain. *Biopolymers* **1979**, *18*, 1859–1876.
12. Ramírez, F. J.; Tuñón, I.; Collado, J. A.; Silla, E. Structural and Vibrational Study of the Tautomerism of Histamine Free-Base in Solution. *J. Am. Chem. Soc.* **2003**, *125*, 2328–2340.
13. Mesu, J. G.; Visser, T.; Soulimani, F.; Weckhuysen, B. M. Infrared and Raman Spectroscopic Study of pH-Induced Structural Changes of L-Histidine in Aqueous Environment. *Vib. Spectrosc.* **2005**, *39* (1), 114–125.
14. Chinsky, L.; Turpin, P. Y.; Duquesne, M.; Brahms, J. Nucleic Acid Derivatives Studied by Preresonance and Resonance Raman Spectroscopy in the Ultraviolet Region. *Biopolymers* **1978**, *17*, 1347–1359.
15. Tsuboi, M.; Hirakawa, A. Y.; Nishimura, Y.; Harada, I. On the Excited-State Geometry of the Adenine Residue as Revealed by a Preresonance Raman Effect. *J. Raman Spectrosc.* **1974**, *2*, 609–620.
